# Prognostic Impacts of Angiotensin-Converting Enzyme Inhibitors and Angiotensin Receptor Blockers in Acute Coronary Syndrome Patients Without Heart Failure

**DOI:** 10.3389/fphar.2022.663811

**Published:** 2022-04-05

**Authors:** Runzhen Chen, Chen Liu, Peng Zhou, Jiannan Li, Jinying Zhou, Ying Wang, Xiaoxiao Zhao, Yi Chen, Shaodi Yan, Li Song, Hanjun Zhao, Hongbing Yan

**Affiliations:** ^1^ Department of Cardiology, Fuwai Hospital, National Center for Cardiovascular Diseases, Peking Union Medical College and Chinese Academy of Medical Sciences, Beijing, China; ^2^ Fuwai Hospital, Chinese Academy of Medical Sciences, Shenzhen, China; ^3^ Coronary Heart Disease Center, Fuwai Hospital, Chinese Academy of Medical Sciences, Beijing, China

**Keywords:** acute coronary syndrome (ACS), ACEI (angiotensin-converting enzyme inhibitor), ARB (angiotensin II receptor blocker), heart failure, percutaneous coroanry intervention (PCI)

## Abstract

**Background:** Despite the recommendations from mainstream guidelines, the use of angiotensin-converting enzyme inhibitors (ACEI) and angiotensin receptor blockers (ARB) for acute coronary syndrome (ACS) patients without heart failure (HF) is controversial, as its evidence is lacking in the era of reperfusion and intensive secondary preventions. This study aimed to investigate the impacts of ACEI/ARB on outcomes of ACS patients without HF treated by percutaneous coronary intervention (PCI).

**Methods:** A total of 2,397 non-HF ACS patients treated by PCI were retrospectively recruited. Prognostic impacts of ACEI/ARB were assessed by unadjusted analysis, followed by propensity score matching (PSM) and propensity score matching weight (PSMW) analysis to control the between-group differences. The primary outcome was a composite of all-cause death and recurrent myocardial infarction (MI).

**Results:** Among the included patients, 1,805 (75.3%) were prescribed with ACEI/ARB at discharge. The median follow-up time was 727 (433–2016) days, with 129 (5.4%) primary endpoint events, consisting of 55 (2.3%) cases of all-cause death and 74 (3.1%) cases of recurrent MI. The use of ACEI/ARB was not associated with significant risk reduction of primary endpoint events in unadjusted analysis (hazard ratio [HR]: 0.95, 95% confidence interval [CI]: 0.64–1.39, *p* = 0.779), PSM analysis (HR: 0.94, 95% CI: 0.60–1.47, *p* = 0.784), and PSMW analysis (HR: 0.91, 95% CI: 0.55–1.49, *p* = 0.704). Similar results were observed for secondary outcomes of all-cause death, cardiac death, and recurrent MI.

**Conclusion:** For ACS patients without HF, the use of ACEI/ARB was not associated with lower risk of death or recurrent MI after PCI.

## Introduction

According to most of the mainstream guidelines, angiotensin-converting enzyme inhibitors (ACEI) and angiotensin receptor blockers (ARB) are generally recommended for all patients with acute coronary syndrome (ACS) unless contraindicated as they could effectively reduce the mortality and risk of recurrent myocardial infarction (MI), which has been demonstrated in many randomized clinical trials (Amsterdam et al., 2014; Ibanez et al., 2018; O'Gara et al., 2013; Collet et al., 2021; Bangalore et al., 2017). However, most of the evidence supporting these recommendations comes from studies of patients with substantially impaired cardiac function (i.e., ejection fraction [EF] < 35–40%) who were not treated by modernized secondary preventive measures, including percutaneous coronary intervention (PCI), dual antiplatelet therapy (DAPT), and lipid-lowering medications (Pfeffer et al., 1992; Yusuf et al., 1992; Ball et al., 1993; Rutherford et al., 1994). Accumulating evidence shows that ACEI/ARB is not effective for reducing mortality in ACS patients with a baseline EF > 40%, casting doubts on whether these agents should be routinely used as one of the long-term medications in every patient after the index coronary event (Parashar et al., 2015; Bangalore et al., 2017; Park et al., 2018; Cespón-Fernández et al., 2019; Raposeiras-Roubín et al., 2020). Meanwhile, over half of the patients acquire generally normal cardiac function with standard care after ACS, which could possibly attenuate the clinical significance for the blockade of renin-angiotensin system (RAS) with ACEI/ARB medications (Sutton et al., 2016; Chen et al., 2020). As the evidence is scarce regarding the routine use of ACEI/ARB in patients with normal left ventricular function in this context, the current study aimed to evaluate the impacts of ACEI/ARB on outcomes of ACS patients without heart failure (HF) after PCI.

## Methods

### Study Cohort

This observational study was conducted in a large-volume PCI center at a national tertiary care institute (Fuwai Hospital, Beijing) specializing in cardiovascular diseases, which has enrolled all patients undergoing emergent coronary angiography and PCI procedures from January 2010 to June 2017. ACS consisted of ST-segment elevation MI (STEMI) and non-ST-elevation ACS, while the diagnosis and classification of ACS is made according to guidelines and universal definitions up to date (Ibanez et al., 2018; Thygesen et al., 2019; Collet et al., 2021), including criteria of clinical presentations, typical characteristics on electrocardiogram, dynamical changes of cardiac enzymes, and imaging evidence. The current study included all patients diagnosed with ACS and subsequently undergoing emergent coronary angiography and PCI. The exclusion criteria were: 1) patients without available EF measurements, 2) patients who died during the index hospitalization, 3) patients with HF (definitions see below), and 4) patients having no follow-up records. The study was performed in accordance with principles set forth in the Declaration of Helsinki, and was approved by the ethics committee of the institute. All patients had signed the written informed consents during hospitalizations regarding the use of clinical data for the purpose of scientific research by the institute.

### Definitions of Heart Failure and Examinations of Cardiac Function

Heart failure, in this study, was defined by the presence of any one of the following conditions: 1) a Killip II classification or above, 2) a measurement of EF < 50%, or 3) a presentation of HF symptoms in need of diuretics during the index hospitalization. After the emergent PCI procedure, patients were subsequently admitted to the coronary care unit. EF was measured by experienced technicians with transthoracic echocardiography on the first day after the index PCI procedure. The EF value, Killip classifications, clinical symptoms, and usage of diuretics were retrieved from the electronic medical record system to allow a comprehensive evaluation of patients’ cardiac function. A group of physicians (R.Z. Chen, J.Y. Zhou, and C. Liu) assessed the patient records and decided the classification of HF according to the definitions, and consensus was achieved through discussions in case of a dispute.

### Outcomes and Follow-Up

The primary outcome for the current analysis was a composite endpoint of all-cause death and recurrent MI. The secondary outcomes included all-cause death, cardiac death, and recurrent MI. Patients were routinely followed up at 1, 6, and 12 months after discharge. The follow-up was completed independently by staffs of the information center at the institute using standardized questionnaires through phone-call interview, and the outcome data was then transferred to the research group on a monthly basis. Follow-up was also performed during rehospitalizations and outpatient visits at the institute due to adverse events or re-examinations. For those who survived more than a year, the subsequent follow-up would be made annually. A group of physicians (R.Z. Chen, J.Y. Zhou, and C. Liu) routinely assessed the reported adverse events. In case of a dispute, a consensus was reached through discussions.

### Statistical Analysis

All the statistical analyses were performed using R 3.6.0 (R Core Team, Vienna, Austria) and Stata 15.0 (StataCorp, College Station, TX, United States). Multiple imputations were performed for missing values of lab test results using the *mi* command of Stata. The propensity score matching (PSM) was performed to control the between-group differences. Briefly, a logistic model was built to generate propensity score (PS) using all the collected baseline variables, indicating the probability of each patient being prescribed with ACEI/ARB before discharge (Austin, 2011). After that, patients not receiving ACEI/ARB were matched to those treated with ACEI/ARB by one-to-one matching using the nearest available pair matching method, with a caliper of 0.2×logit (PS). Receiver operating curve analysis was performed, and the area under the curve (AUC) was calculated to assess the performance of the PSM model. A matching weight was assigned to each patient based on the PS, and the propensity score matching weight (PSMW) analysis was performed to further balance the differences between two groups (Li and Greene, 2013). Density plots were drawn to compare the distribution of PS between ACEI/ARB users and non-users after matching and weighing. An absolute standardized mean difference (SMD) of 10% or less was considered to indicate an appropriate balance for between-group differences. Kaplan-Meier survival curve analysis was performed for the original dataset. Bivariable Cox regression was performed to assess the prognostic impacts of ACEI/ARB on various clinical outcomes in the original, PSM and PSMW datasets, respectively. Subgroup analysis for the primary outcome was performed across high-risk indications of ACEI/ARB (i.e., diabetes, hypertension, anterior infarction) and types of RAS inhibitors. Categorical variables are presented as numbers (%). Continuous variables are presented using mean ± SD if they follow the normal distribution. Otherwise, they are presented as medians with the 25th and 75th percentiles. A two-tailed *p*-value < 0.05 was considered statistically significant.

## Results

### Patient Cohort and Baseline Characteristics

From January 2010 to June 2017, a total of 4,151 patients underwent emergent coronary angiography and PCI due to ACS at the institute. Among these patients, five patients did not have available EF measurements, and 55 patients died during the index hospitalization. For the remaining 4,091 patients, 1,647 patients were excluded due to HF according to definitions, and 47 patients did not have follow-up records of any forms (i.e., phone-call interview, outpatient visits or re-hospitalizations at the institute). Finally, a total of 2,397 patients were included in the final analysis.

Baseline characteristics stratified by ACEI/ARB medication was shown in Table 1. Overall, ACEI/ARB was prescribed to 1805 (75.3%) patients, among whom 1,591 (88.1%) were prescribed with ACEI and 214 (11.9%) patients were prescribed with ARB (Supplementary Tables S1). Captopril (40.9%) was the most frequently prescribed ACEI, followed by imidapril (18.3%), ramipril (10.0%), perindopril (9.6%), and benazepril (7.1%), while fosinopril (1.7%) and enalapril (0.5%) were least frequently used. For ARB users, telmisartan (5.3%) and losartan (3.2%) were the major types being prescribed, while other types of ARB (i.e., candesartan, irbesartan, olmesartan and valsartan) were less frequently used. According to the equivalent dosages (Houston et al., 2017; Healthcare I, 2021), most of the ACEI/ARB users (91.6%) were on low-dose regimes. Compared with non-users, patients prescribed with ACEI/ARB at discharge had higher prevalence of hypertension, more stable hemodynamic status (i.e., lower heart rate, higher blood pressure), lower level of systemic inflammation, and less cardiac damage. Slight but significant difference in EF was observed between the two groups. Distributions of culprit lesions were also different, mainly due to more culprit lesions at left anterior descending arteries (32.6% vs. 24.0%), but less at right coronary arteries (RCA, 44.8% vs. 54.6%) in ACEI/ARB users compared with non-users. Moreover, patients not treated by ACEI/ARB medications acquired worse pre-intervention TIMI blood flow and longer door-to-balloon time. For discharge medications, ACEI/ARB users were more often prescribed with β-blockers, while small but significant difference in the use P2Y12 inhibitors was observed between two groups. Substantial between-group differences (SMD >0.1) were detected for many baseline variables, and therefore, analysis by PSM and PSMW was necessary.

**TABLE 1:** Baseline characteristics of patients in the original dataset stratified by ACEI/ARB medication.

Variables	All patients (*N* = 2,397)	No ACEI/ARB (*N* = 592)	ACEI/ARB (*N* = 1,805)	SMD	*p* Value
Age, years	57.8 ± 11.6	58.2 ± 11.4	57.6 ± 11.6	0.046	0.33
Male sex, n (%)	1,945 (81.1)	474 (80.1)	1,471 (81.5)	0.036	0.44
Hypertension, n (%)	1,426 (59.5)	237 (40.0)	1,189 (65.9)	0.536	<0.001
Diabetes, n (%)	749 (31.2)	181 (30.6)	568 (31.5)	0.019	0.680
History of PCI or CABG, n (%)	319 (13.3)	66 (11.1)	253 (14.0)	0.087	0.075
Peripheral artery diseases, n (%)	95 (4.0)	21 (3.5)	74 (4.1)	0.029	0.55
STEMI, n (%)	2,049 (85.5)	503 (85.0)	1,546 (85.7)	0.019	0.68
Hemodynamics					
Heart rate, bpm	74.6 ± 13.5	76.2 ± 14.8	74.0 ± 13.0	0.155	<0.001
SBP, mmHg	125.2 ± 17.6	119.1 ± 16.8	127.2 ± 17.5	0.471	<0.001
DBP, mmHg	74.7 ± 12.3	71.5 ± 11.4	75.8 ± 12.4	0.363	<0.001
EF, %	57.8 ± 4.1	57.4 ± 4.1	57.9 ± 4.1	0.114	0.016
Cardiac arrest, n (%)	58 (2.4)	19 (3.2)	39 (2.2)	0.065	0.15
Lab tests					
Creatinine, μmoI/L	80.1 ± 21.1	80.5 ± 25.0	79.9 ± 19.7	0.023	0.60
eGFR, ml/min	86.5 (73.1–99.6)	86.0 (73.7–100.4)	86.7 (72.8–99.4)	0.046	0.93
LDL-C, mmol/L	2.7 ± 0.9	2.7 ± 0.9	2.7 ± 0.9	0.041	0.39
hsCRP, mg/L	5.45 (2.41–11.35)	6.43 (2.50–11.79)	5.28 (2.38–11.21)	0.101	0.016
D-dimer, ng/mL	300 (220–461)	310 (220–490)	300 (220–460)	0.044	0.32
Peak cTnI, ng/mL	2.15 (0.55–7.01)	2.45 (0.70–8.46)	2.04 (0.51–6.49)	0.115	0.015
Coronary angiography findings					
Culprit lesion, n (%)					
LM	35 (1.5)	12 (2.0)	23 (1.3)	0.227	<0.001
LAD	730 (30.5)	142 (24.0)	588 (32.6)		
LCX	494 (20.6)	114 (19.3)	380 (21.1)		
RCA	1,132 (47.2)	323 (54.6)	809 (44.8)		
Bypass grafts	6 (0.3)	1 (0.2)	5 (0.3)		
Multivessel diseases, n (%)	1,796 (74.9)	446 (75.3)	1,350 (74.8)	0.013	0.79
Pre-PCI TIMI 0 flow, n (%)	1,504 (62.7)	394 (66.6)	1,110 (61.5)	0.106	0.027
Post-PCI TIMI 3 flow, n (%)	2,332 (97.3)	577 (97.5)	1,755 (97.2)	0.015	0.76
D2B time ≥120 min, n (%)	1,372 (57.2)	320 (54.1)	1,052 (58.3)	0.085	0.071
CR before discharge, n (%)	1,083 (45.2)	259 (43.8)	824 (45.6)	0.038	0.42
Medications at discharge					
Aspirin, n (%)	2,383 (99.4)	587 (99.2)	1,796 (99.5)	0.042	0.34
P2Y_12_ inhibitors, n (%)	2,384 (99.5)	583 (98.5)	1,801 (99.8)	0.140	<0.001
Statins, n (%)	2,255 (94.1)	554 (93.6)	1,701 (94.2)	0.027	0.56
β blockers, n (%)	2,060 (85.9)	465 (78.5)	1,595 (88.4)	0.267	<0.001

ACEI = angiotensin-converting enzyme inhibitor, ARB = angiotensin receptor blocker, SMD = standardized mean difference, PCI = percutaneous coronary intervention, CABG = coronary artery bypass grafting, STEMI = ST-segment elevation myocardial infarction, SBP = systolic blood pressure, DBP = diastolic blood pressure, EF = ejection fraction, eGFR = estimated glomerular filtration rate, LDL-C = low-density lipoprotein cholesterol, hsCRP = high-sensitivity C-reactive protein, cTnI = cardiac troponin I, LM = left main, LAD = left anterior descending artery, LCX = left circumflex, RCA = right coronary artery, TIMI flow = Thrombolysis In Myocardial Infarction grade flow, D2B time = door-to-balloon time, CR = complete revascularization.

### Post-Discharge Clinical Outcomes

**FIGURE 1:**
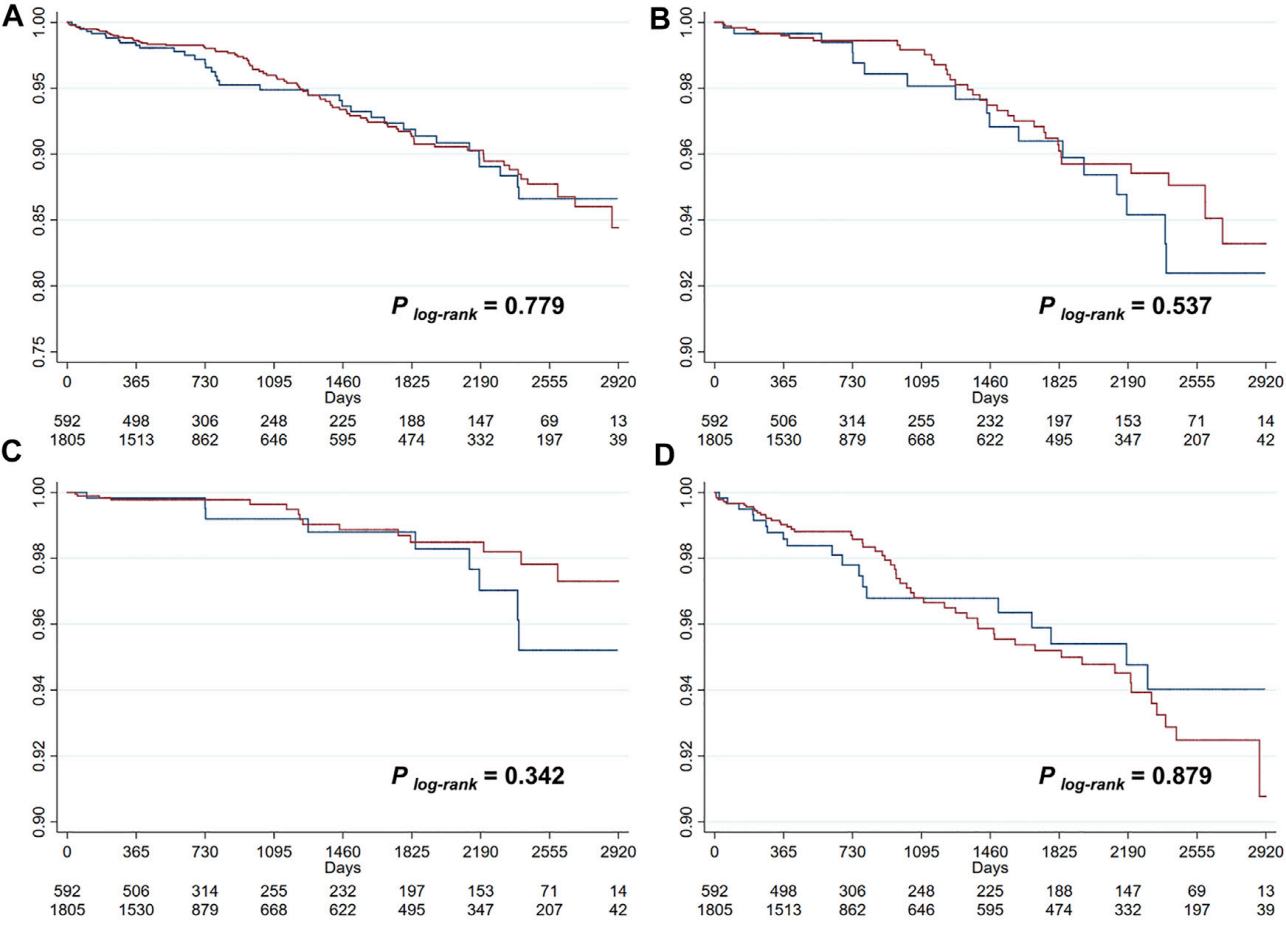
Kaplan-Meier survival curve analysis for the primary outcome **(A)**, all-cause death **(B)**, cardiac death **(C)**, and recurrent myocardial infarction **(D)**.

**FIGURE 2:**
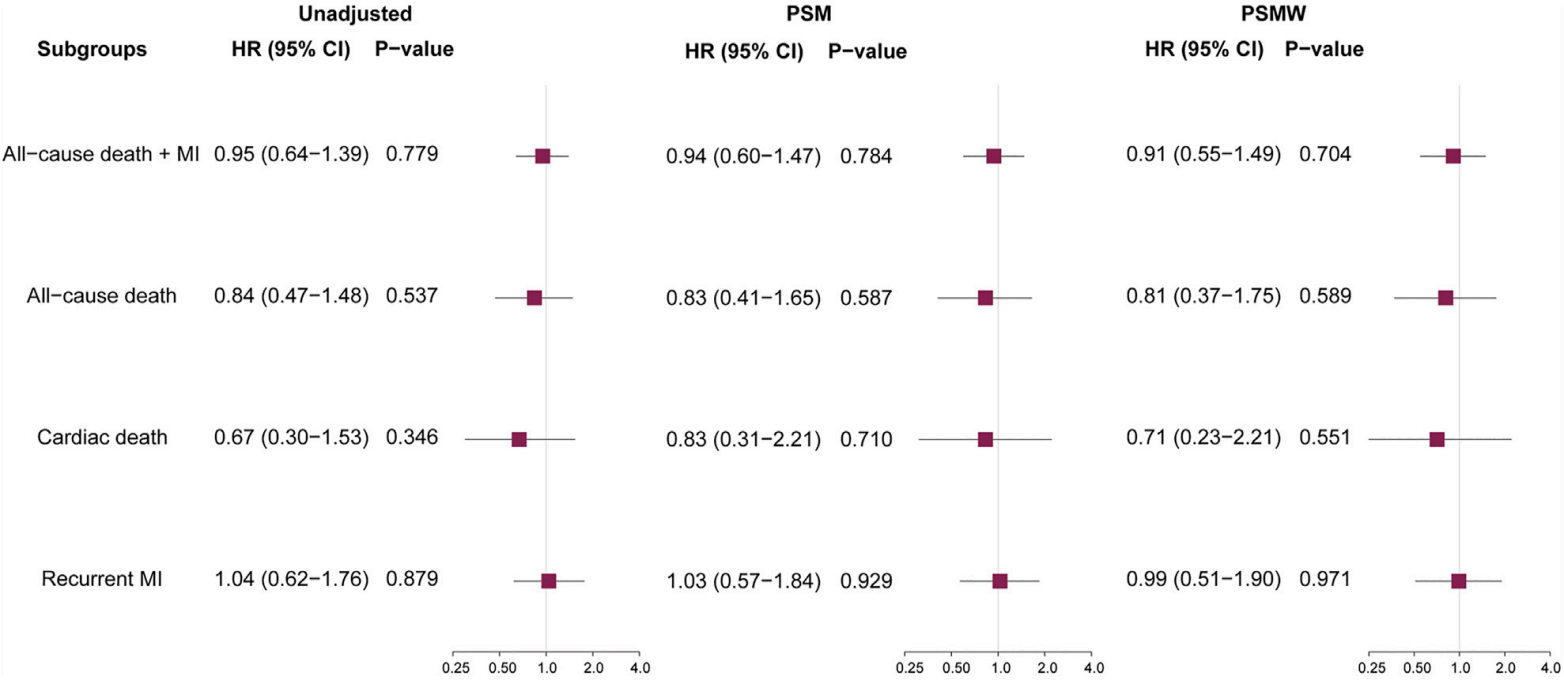
Prognostic impacts of ACEI/ARB on various outcomes. ACEI = angiotensin converting enzyme inhibitors, ARB = angiotensin receptor blockers, HR = hazard ratio, CI = confidence interval, MI = myocardial infarction, PSM = propensity score matching, PSMW = propensity score matching weight.

During a median follow-up of 727 (433–2016) days, there were 129 (5.4%, incidence rate [IR]: 16.79/1000-person-year) primary endpoint events, composed of 55 (2.3%, IR: 7.16/1000-person-year [PY]) cases of all-cause death, 25 (1.0%, IR: 3.25/1000PY) cases of cardiac death, and 74 (3.1%, IR: 9.63/1000PY) cases of recurrent MI. Unadjusted Kaplan-Meier survival estimates did not demonstrate significant reduction in primary or secondary endpoint events for patients on ACEI/ARB treatments (Figure 1). Univariable Cox regression (Figure 2) also showed that the use of ACEI/ARB was not associated with lower risk of primary endpoint events (hazard ratio [HR]: 0.95, 95% confidence interval [CI]: 0.64–1.39, *p* = 0.779), with similar results for all-cause death (HR: 0.84, 95% CI: 0.47–1.48, *p* = 0.537), cardiac death (HR: 0.67, 95% CI: 0.30–1.53, *p* = 0.346), and recurrent MI (HR: 1.04, 95% CI: 0.62–1.76, *p* = 0.879).

**FIGURE 3:**
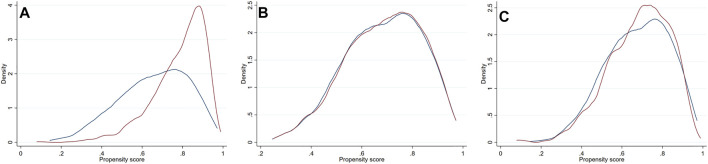
Density plots for the distribution of propensity score stratified by usage of ACEI/ARB in the original dataset **(A)**, the PSM dataset **(B)** and PSMW dataset **(C)**. Red line = ACEI/ARB users, blue line = ACEI/ARB non-users. ACEI = angiotensin converting enzyme inhibitors, ARB = angiotensin receptor blockers, PSM = propensity score matching, PSMW = propensity score matching weight.

As significant differences (SMD >0.1) were detected for many baseline variables (Table 1), PSM was used to control for between-group imbalances. Before the PSM, the median propensity to be prescribed with ACEI/ARB was substantially higher for ACEI/ARB users (0.816 [0.717–0.882]) compared with non-users (0.676 [0.539–0.790], *p* < 0.001), and the distribution of PS was apparently different for two groups (Figure 3A). After the PSM procedure, a total of 543 pairs of ACEI/ARB users and non-users were matched. The between-group balances were achieved for most variables (Table 2), but residual differences remained for peripheral artery disease (SMD: 0.108), STEMI (SMD: 0.112), diastolic blood pressure (SMD: 0.142), EF (SMD: 0.110), estimated glomerular filtration rate (SMD: 0.112), high-sensitivity C-reactive protein (SMD: 0.158), and pre-PCI TIMI 0 flow (SMD: 0.131). No significant difference in propensity was detected for the two groups after PSM (0.698 [0.583–0.801] vs. 0.696 [0.578–0.801], *p* = 0.720), which was further confirmed by the similar distribution curves of propensity in the density plot (Figure 3B). The AUC for the PSM model was 0.72 (0.70–0.75), suggesting an adequate discrimination to differentiate ACEI/ARB users from non-users (Supplementary Figure S1). According to the PSM bivariable analysis (Figure 2), the use of ACEI/ARB was not associated with significant risk reduction in death or MI (HR: 0.94, 95% CI: 0.60–1.47, *p* = 0.784). Similar findings were observed for all-cause death (HR: 0.83, 95% CI: 0.41–1.65, *p* = 0.587), cardiac death (HR: 0.83, 95% CI: 0.31–2.21, *p* = 0.710), and recurrent MI (HR: 1.03, 95% CI: 0.57–1.84, *p* = 0.929).

**TABLE 2:** Baseline characteristics of patients in the propensity score matching dataset stratified by ACEI/ARB medication.

Variables	All patients (N = 1,086)	No ACEI/ARB (N = 543)	ACEI/ARB (N = 543)	SMD	*p*-value
Age, years	58.1 ± 11.4	58.1 ± 11.4	58.1 ± 11.4	0.002	0.97
Male sex, n (%)	868 (79.9)	434 (79.9)	434 (79.9)	<0.001	1.00
Hypertension, n (%)	480 (44.2)	233 (42.9)	247 (45.5)	0.052	0.39
Diabetes, n (%)	337 (31.0)	166 (30.6)	171 (31.5)	0.020	0.74
Peripheral artery diseases, n (%)	101 (9.3)	59 (10.9)	42 (7.7)	0.108	0.076
History of PCI or CABG, n (%)	45 (4.1)	21 (3.9)	24 (4.4)	0.028	0.65
STEMI, n (%)	952 (87.7)	466 (85.8)	486 (89.5)	0.112	0.065
Hemodynamics					
Heart rate, bpm	75.6 ± 13.9	75.4 ± 14.0	75.8 ± 13.8	0.027	0.65
SBP, mmHg	119.9 ± 16.3	119.9 ± 16.6	119.9 ± 16.0	0.002	0.98
DBP, mmHg	71.0 ± 11.5	71.8 ± 11.4	70.2 ± 11.5	0.142	0.020
EF, %	57.3 ± 4.1	57.5 ± 4.1	57.1 ± 4.2	0.110	0.071
Cardiac arrest, n (%)	34 (3.1)	18 (3.3)	16 (2.9)	0.021	0.73
Lab tests					
Creatinine, μmoI/L	80.6 ± 22.8	79.8 ± 23.2	81.4 ± 22.4	0.071	0.24
eGFR, ml/min	85.4 (71.5–99.3)	86.5 (74.2–100.8)	84.3 (68.9–98.2)	0.112	0.014
LDL-C, mmol/L	2.7 ± 0.9	2.7 ± 0.9	2.7 ± 0.9	0.021	0.73
hsCRP, mg/L	6.74 (2.73–11.83)	6.42 (2.40–11.69)	7.23 (3.08–11.97)	0.158	0.050
D-dimer, ng/mL	308 (210–470)	310 (220–470)	300 (200–467)	0.020	0.50
Peak cTnI, ng/mL	2.34 (0.80–6.45)	2.45 (0.69–8.37)	2.24 (0.90–5.21)	0.008	0.36
Coronary angiography findings					
Culprit lesion, n (%)					
LM	14 (1.3)	8 (1.5)	6 (1.1)	0.071	0.85
LAD	270 (24.9)	140 (25.8)	130 (23.9)		
LCX	210 (19.3)	108 (19.9)	102 (18.8)		
RCA	590 (54.3)	286 (52.7)	304 (56.0)		
Bypass grafts	2 (0.2)	1 (0.2)	1 (0.2)		
Multivessel diseases, n (%)	815 (75.0)	406 (74.8)	409 (75.3)	0.013	0.83
Pre-PCI TIMI 0 flow, n (%)	743 (68.4)	355 (65.4)	388 (71.5)	0.131	0.031
Post-PCI TIMI 3 flow, n (%)	1,057 (97.3)	530 (97.6)	527 (97.1)	0.034	0.57
D2B time ≥120 min, n (%)	592 (54.5)	296 (54.5)	296 (54.5)	<0.001	1.00
CR before discharge, n (%)	488 (44.9)	239 (44.0)	249 (45.9)	0.037	0.54
Medications at discharge					
Aspirin, n (%)	1,079 (99.4)	539 (99.3)	540 (99.4)	0.023	0.70
P2Y_12_ inhibitors, n (%)	1,082 (99.6)	541 (99.6)	541 (99.6)	<0.001	1.00
Statins, n (%)	1,021 (94.0)	513 (94.5)	508 (93.6)	0.039	0.52
β blockers, n (%)	885 (81.5)	439 (80.8)	446 (82.1)	0.033	0.58

ACEI = angiotensin-converting enzyme inhibitor, ARB = angiotensin receptor blocker, SMD = standardized mean difference, PCI = percutaneous coronary intervention, CABG = coronary artery bypass grafting, STEMI = ST-segment elevation myocardial infarction, SBP = systolic blood pressure, DBP = diastolic blood pressure, EF = ejection fraction, eGFR = estimated glomerular filtration rate, LDL-C = low-density lipoprotein cholesterol, hsCRP = high-sensitivity C-reactive protein, cTnI = cardiac troponin I, LM = left main, LAD = left anterior descending artery, LCX = left circumflex, RCA = right coronary artery, TIMI flow = Thrombolysis In Myocardial Infarction grade flow, D2B time = door-to-balloon time, CR = complete revascularization.

As there were still residual between-group differences in the PSM dataset, we performed PSMW analysis to further reduce the systematic differences in baseline characteristics. Subsequently, all variables achieved an SMD below 0.1 (Table 3), and similar distribution of propensity for both groups was affirmed in the density plot (Figure 3C). Still, ACEI/ARB users did not acquire lower risk of death or MI (HR: 0.91, 95% CI: 0.55–1.49, *p* = 0.704), and the results remained similar for all-cause death (HR: 0.81, 95% CI: 0.37–1.75, *p* = 0.589, Figure 2), cardiac death (HR: 0.71, 95% CI: 0.23–2.21, *p* = 0.551) and recurrent MI (HR: 0.99, 95% CI: 0.51–1.90, *p* = 0.971).

**TABLE 3:** Baseline characteristics of patients in the propensity matching weight dataset stratified by ACEI/ARB medication.

Variables	All patients (N = 1,107.40)	No ACEI/ARB (N = 556.02)	ACEI/ARB (N = 551.38)	SMD
Age, years	58.1 ± 11.5	58.1 ± 11.4	58.1 ± 11.7	0.005
Male sex, n (%)	884.1 (79.8)	445.3 (80.1)	438.8 (79.6)	0.013
Hypertension, n (%)	469.7 (42.4)	234.7 (42.2)	235.0 (42.6)	0.008
Diabetes, n (%)	344.9 (31.1)	172.4 (31.0)	172.5 (31.3)	0.006
History of PCI or CABG, n (%)	122.3 (11.0)	61.2 (11.0)	61.2 (11.1)	0.003
Peripheral artery diseases, n (%)	42.0 (3.8)	20.2 (3.6)	21.8 (4.0)	0.017
STEMI, n (%)	948.2 (85.6)	474.4 (85.3)	473.8 (85.9)	0.018
Hemodynamics				
Heart rate, bpm	75.6 ± 14.1	75.7 ± 14.1	75.6 ± 14.0	0.011
SBP, mmHg	119.9 ± 16.2	119.9 ± 16.6	120.0 ± 15.8	0.007
DBP, mmHg	71.9 ± 11.5	71.8 ± 11.4	72.0 ± 11.7	0.012
EF, %	57.5 ± 4.0	57.5 ± 4.1	57.5 ± 4.0	0.009
Cardiac arrest, n (%)	33.0 (3.0)	16.8 (3.0)	16.2 (2.9)	0.004
Lab tests				
Creatinine, μmoI/L	80.0 ± 21.7	80.0 ± 23.6	79.9 ± 19.6	0.005
eGFR, ml/min	86.2 (73.3–99.9)	86.2 (74.0–100.5)	86.2 (72.2–99.0)	0.027
LDL-C, mmol/L	2.7 ± 0.9	2.7 ± 0.9	2.7 ± 0.9	0.005
hsCRP, mg/L	6.07 (2.53–11.69)	6.54 (2.50–11.89)	5.96 (2.74–11.65)	0.010
D-dimer, ng/mL	310 (210–480)	310 (220–490)	310 (220–480)	0.011
Peak cTnI, ng/mL	2.53 (0.70–8.16)	2.56 (0.70–8.85)	2.60 (0.72–7.92)	0.008
Coronary angiography findings				
Culprit lesion, *n* (%)				
LM	17.7 (1.6)	9.0 (1.6)	8.7 (1.6)	0.017
LAD	279.1 (25.2)	140.0 (25.2)	139.1 (25.2)	
LCX	221.2 (20.0)	110.4 (19.9)	110.8 (20.1)	
RCA	586.9 (53.0)	295.6 (53.2)	291.4 (52.8)	
Bypass grafts	2.4 (0.2)	1.0 (0.2)	1.4 (0.2)	
Multivessel diseases, n (%)	835.0 (75.4)	417.5 (75.1)	417.5 (75.7)	0.015
Pre-PCI TIMI 0 flow, n (%)	729.7 (65.9)	365.0 (65.6)	364.7 (66.1)	0.011
Post-PCI TIMI 3 flow, n (%)	1,079.2 (97.5)	541.9 (97.5)	537.4 (97.5)	< 0.001
D2B time ≥120 min, n (%)	603.2 (54.5)	303.0 (54.5)	300.2 (54.4)	0.001
CR before discharge, n (%)	487.4 (44.0)	245.1 (44.1)	242.3 (43.9)	0.003
Medications at discharge				
Aspirin, n (%)	1,099.9 (99.3)	552.1 (99.3)	547.8 (99.4)	0.008
P2Y_12_ inhibitors, n (%)	1,098.7 (99.2)	551.3 (99.2)	547.4 (99.3)	0.014
Statins, n (%)	1,045.3 (94.4)	524.3 (94.3)	521.0 (94.5)	0.009
β blockers, n (%)	897.0 (81.0)	448.7 (80.7)	448.4 (81.3)	0.016

ACEI = angiotensin-converting enzyme inhibitor, ARB = angiotensin receptor blocker, SMD = standardized mean difference, PCI = percutaneous coronary intervention, CABG = coronary artery bypass grafting, STEMI = ST-segment elevation myocardial infarction, SBP = systolic blood pressure, DBP = diastolic blood pressure, EF = ejection fraction, eGFR = estimated glomerular filtration rate, LDL-C = low-density lipoprotein cholesterol, hsCRP = high-sensitivity C-reactive protein, cTnI = cardiac troponin I, LM = left main, LAD = left anterior descending artery, LCX = left circumflex, RCA = right coronary artery, TIMI flow = Thrombolysis In Myocardial Infarction grade flow, D2B time = door-to-balloon time, CR = complete revascularization.

**FIGURE 4:**
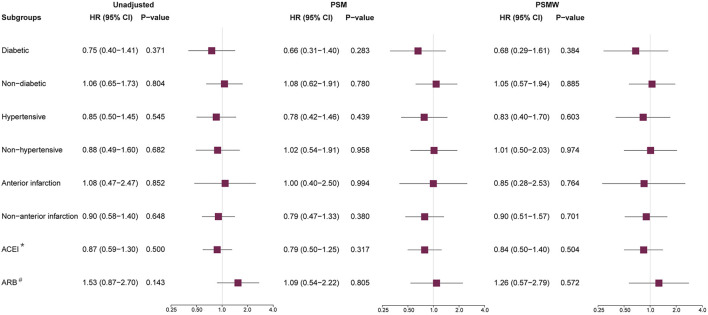
Subgroup analysis for the primary outcome. ACEI = angiotensin converting enzyme inhibitors, ARB = angiotensin receptor blockers, PSM = propensity score matching, PSMW = propensity score matching weight, HR = hazard ratio, CI = confidence interval. *: ACEI users vs. ACEI/ARB non-users. #: ARB users vs. ACEI/ARB non-users.

Subgroup analysis was performed for the primary outcome across high-risk indications for ACEI/ARB (including diabetes, hypertension, and anterior infarction) and types of RAS inhibitors being prescribed. Systematic differences of baseline characteristics were common between ACEI/ARB users and non-users in various subgroups (Supplementary Tables S2–S9). PSM analysis was performed, and the AUC for PSM models in various subgroups were generally above 0.7, suggesting a good discrimination for patients with or without ACEI/ARB medications (Supplementary Figure S2). Although there were residual differences for some variables (Supplementary Tables S10–S17), the propensity was generally well-matched after the PSM procedure (Supplementary Figures S3–S10). With PSMW, both the propensity (Supplementary Figures S3–S10) and baseline differences were well controlled (Supplementary Table S18–S25). Still, the risk reduction by ACEI/ARB was not significant across the unadjusted, PSM and PSMW analyses, despite the complication of diabetes, hypertension, or anterior infarction (Figure 4). Moreover, neither the usage of ACEI nor ARB was associated with lower risk of all-cause death or recurrent MI as compared with patients without RAS blockade.

## Discussions

In this observational study of ACS patients without HF treated by PCI, the use of ACEI/ARB was not associated with significant risk reduction of all-cause death, cardiac death, or recurrent MI. The neutral effect of ACEI/ARB was not altered in the PSM analysis, PSMW analysis, or subgroup analysis.

### Gaps of Evidence for ACEI/ARB in Non-HF ACS Patients

Based on evidences from the era of thrombolysis (Pfeffer et al., 1992; Yusuf et al., 1992; Ball et al., 1993; Rutherford et al., 1994), ACEI/ARB is currently indicated for nearly all patients having experienced an acute coronary event (Amsterdam et al., 2014; Ibanez et al., 2018; O'Gara et al., 2013; Collet et al., 2021), with the expectation to control blood pressure, inhibit the left ventricular remodeling, suppress cardiovascular impacts of RAS system, and therefore bring about anti-atherosclerotic and cardioprotective effects (Thind, 1990; Lamas and Pfeffer, 1991; Nissen et al., 2004). However, the long-term benefit of ACEI/ARB has been questioned with the rapid development of revascularization techniques and the application of more intensive antiplatelet and lipid-lowering treatment (Braunwald et al., 2004; Raposeiras-Roubín et al., 2020). Meanwhile, the incidence of cardiac failure after ACS is also declining in recent years (Chen et al., 2020). Notably, studies in the field of HF suggest that traditionally recommended treatments (e.g., ACEI/ARB, β-blockers) might not be effective for HF patients with preserved or mid-range EF (Ponikowski et al., 2016; Ambrosy et al., 2018; Lund et al., 2018). It is therefore necessary to reassess the actual benefit for long-term use of ACEI/ARB after discharge, and reconsider the recommendations for its routine prescription among patients with generally normal cardiac function after ACS.

Previously, Roubin et al. demonstrated in the BleeMACS study that RAS blockers could not reduce 1-year mortality of ACS patients treated by PCI (HR: 0.84, 95% CI: 0.65–1.08) (Raposeiras-Roubín et al., 2020). Similarly, Parashar et al. suggest a limited reduction of mortality by ACEI/ARB for STEMI patients with EF > 40% (HR: 0.88, 95% CI: 0.57–1.36), with 714 patients needed to treat for preventing a case of death at 1 year (Parashar et al., 2015). Considering the new classification of HF, the current study used stricter inclusion criteria, which only incorporated patients with EF ≥ 50% and no clinical signs of HF, in order to minimize the chance of incorrect recruitment of HF patients with preserved (≥50%) or mid-range EF (40–49%). Nearly 60% of ACS-PCI patients in the original cohort were not complicated with clinical HF according to current definitions, among whom ACEI/ARB was not associated with the reduction in long-term risk of death and recurrent MI, which remained the same after matching and weighted analysis. The point estimates of HR for various outcomes in the current study generally fall within 0.8–1.0, which is in line with previous studies reporting ineffectiveness of ACEI/ARB (Parashar et al., 2015; Raposeiras-Roubín et al., 2020). Although the estimated HR values were lower in several high-risk subgroups (i.e., diabetic, hypertensive, anterior infarction), the wide confidence interval did not support a positive effect of ACEI/ARB for reducing mortality and MI. Moreover, neither the treatment with ACEI nor ARB was associated with fewer adverse events. Taken together, the routine use of ACEI/ARB medications in ACS patients with preserved or normal cardiac function result in limited risk reduction, for which the recommendation should be reconsidered in the era of reperfusion.

### Impacts of More Intensive Secondary Preventions on the Efficacy of ACEI/ARB

Although the interpretation could be challenging, the observed ineffectiveness of ACEI/ARB could be due to the comprehensive advances of ACS treatment in the past decades. In studies demonstrating the reduction of MI by ACEI/ARB, most of patients have been treated noninvasively while not receiving DAPT or statins medications, suggesting an incomplete resolution of ischemia and higher risk of future thrombosis due to inadequate suppression of platelet function and progression of coronary plaques (Pfeffer et al., 1992; Yusuf et al., 1992; Ball et al., 1993; Rutherford et al., 1994; Fox, 2003). In this scenario, ACEI/ARB could have backed up the treatments through its anti-atherosclerotic effects secondary to the blood pressure control and the suppression of neural-hormonal system (Lamas and Pfeffer, 1991; Nissen et al., 2004). However, in the era of PCI, the coronary obstruction due to thrombus at the culprit lesion was instantly resolved by angioplasty or stenting, and the following risk of stent thrombosis, restenosis or progression of atherosclerotic plaques were comprehensively tackled by DAPT and lipid-lowering treatments (Amsterdam et al., 2014; Ibanez et al., 2018; O'Gara et al., 2013; Hoang et al., 2016; Robinson et al., 2014). In the PEACE trial, the addition of trandolapril did not reduce the risk of cardiovascular deaths, MI, or coronary revascularizations (HR: 0.96, 95% CI: 0.88–1.06) in a cohort of patients with stable coronary artery disease (CAD) (Braunwald et al., 2004). However, over 70% of the recruited patients have been treated by revascularizations and lipid-lowering medications. In the BleeMACS study (Raposeiras-Roubín et al., 2020), all the included ACS patients have undergone PCI, while over 90% of them receive DAPT and statins. A recent meta-analysis including 24 randomized trials in stable CAD patients without HF also shows that RAS blockade could effectively reduce mortality, MI, angina, HF, and revascularization when compared with placebos, but not with active controls (Bangalore et al., 2017). For the current cohort, over 90% of patients were on DAPT at discharge, while over 80% of them were prescribed with β-blockers, suggesting a prevalent use of intensive secondary preventions. Consequently, the vasoprotective effects of RAS inhibitors could be attenuated, as medications targeted for platelet inhibition, lipid lowering and plaque stabilization might have provided more specific and overwhelming protection than that of ACEI/ARB. Taken together, routine prescription of ACEI/ARB to ACS patients without HF might not be indispensable under the condition of active secondary preventions, and new evidence is needed to reassess the prognostic impacts of these medications.

### Impacts of Low-Risk Profile on the Efficacy of ACEI/ARB

Aside from intensive secondary preventions, the baseline risk of the targeted patient group is also a decisive factor for the observed risk reduction with ACEI/ARB medications. In a recent meta-analysis, Sripal et al. have reported that the use of RAS inhibitors is only beneficial when the IR of all-cause death and cardiovascular death is higher than 14.10 and 7.65 per 1000-PY in the control group, respectively (Bangalore et al., 2017). Similar trends are observed in studies of non-HF ACS patients. In studies showing significant risk reduction for patients treated by ACEI/ARB, the 1-year mortality of control groups is generally over 10% (Milonas et al., 2010; Grall et al., 2015; Liu et al., 2019). On the contrary, the 1-year mortality of patients without RAS blockade could be as low as 1–3% in studies acquiring neutral findings (Pitt et al., 2001; Parashar et al., 2015; Park et al., 2018; Raposeiras-Roubín et al., 2020). In the current study, the all-cause mortality (2.1 vs. 2.8%) and cardiac mortality (0.9 vs. 1.5%) was both very low for patients with or without ACEI/ARB medication over a median follow-up of nearly 2 years. Besides, the IR of all-cause death (6.72 vs. 8.39 per 1000-PY) and cardiac death (2.83 vs. 4.44 per 1000-PY) was far below the aforementioned thresholds to detect the benefit of RAS inhibitors, which could be a major interpretation for the neutral findings in the current study. Several factors might have contributed the low-risk profile of the current cohort, including the generally preserved cardiac function, the exclusion of patients who failed to survive the hospitalizations, and the prevalent use of antiplatelet, lipid-lowering and anti-ischemic medications. Besides, the risk of left-ventricular remodeling was theoretically lower for the current cohort, since most of the patients had a culprit lesion at left circumflex or RCA (67.8%), while 97.3% of patients reached a post-PCI TIMI 3 grade flow. The median peak cardiac troponin I value was only 2.15 (0.55–7.01) ng/ml, suggesting an adequate resolution of emergent ischemia and limited myocardial damage. The favorable outcomes of reperfusion could have led to a lower risk of post-infarction left ventricular remodeling, for which the RAS blockade brings limited improvement to cardiac function and long-term outcomes even at very high dosages (Ganame et al., 2011; Masci et al., 2011; Park et al., 2018). In sum, non-HF ACS patients treated by PCI possessed an intrinsically lower risk profile, and ACEI/ARB might not be able to further improve patient outcomes in this occasion.

### Impacts of ACEI/ARB Dosages for Outcome Improvements

According to previous research, differences in regimes and dosages of ACEI/ARB prescribed to patients could have affected the clinical efficacy being observed by researchers (Grall et al., 2015; Liu et al., 2019). With most of patients in the treatment group reaching high dosages, results from the HOPE trial (ramipril 10 mg daily) and EUROPA trial (perindopril 8 mg daily) have demonstrated that RAS blockade could reduce adverse events in patients with preserved cardiac function (Yusuf et al., 2000; Fox, 2003). Comparatively, trials assigning lower dosages of RAS inhibitors to the treatment group show limited clinical benefits (Pitt et al., 2001; Nissen et al., 2004; Al-Mallah et al., 2006), like the QUIET trial (quinapril 20 mg daily) and the CAMELOT trial (enalapril 20 mg daily). It seems patients need to be on high-dose regimes to maximize the benefits from ACEI/ARB treatment. In the current study, the majority of patients taking ACEI/ARB were on low-dose regime, which might have posed limited impacts on the outcomes. However, the PEACE trial fails to show benefits for stable CAD patients with preserved left ventricular function to take trandolapril 4 mg daily, a dosage previously demonstrated to improve the survival of hypertensive patients (Braunwald et al., 2004). In recent studies showing ineffectiveness of ACEI/ARB, details of daily regime are generally not available. It remained to be investigated whether improving dosages of ACEI/ARB could increase the benefit for patients on these medications. (Milonas et al., 2010; Parashar et al., 2015; Raposeiras-Roubín et al., 2020).

In sum, the current study called into questions regarding the necessity of routine use of ACEI/ARB for ACS patients without HF after PCI. Physicians could consider not prescribing ACEI/ARB for these low-risk patients if other risk factors could be well-controlled without the RAS blockade. Clinical guidelines should reconsider the recommendations of routine use of ACEI/ARB to all ACS patients, especially those patients with normal cardiac function.

## Limitations

The major limitations of this study are as follow. Firstly, the current study was retrospective, and patients were not randomly assigned to receive ACEI/ARB treatment. Although matching methods of PSM and PSMW were used to control the between-group differences for all the collected baseline variables, unmeasured confounders could still affect the results. Moreover, the current study has no available data regarding the patient adherence or changes of ACEI/ARB medications after discharge. Discontinuation and modification of the treating regime could have attenuated the risk differences between ACEI/ARB users and non-users. Besides, the median 2-year follow-up might not be adequate to fully determine the prognostic impacts of ACEI/ARB in the long run. Finally, this study was accomplished in a single center. Although the sample size was large enough, only Chinese patients were included in this study. The extrapolation of current conclusions still requires further validation. Future multicenter randomized clinical trials are warranted to assess the efficacy of ACEI/ARB in non-HF ACS patients treated by PCI from different regions and populations.

## Conclusion

For ACS patients without HF, the use of ACEI/ARB was not associated with lower risk of death or recurrent MI after PCI.

## Data Availability

The datasets presented in this article are not readily available because; The data used to support the findings of this study are available from the corresponding authors upon request. The institution (Fuwai Hospital) requires all requests for accessing any data of patients be applicated and processed in a case by case manner. Requests to access the datasets should be directed to;hbyanfuwai2018@163.com.
