# Supplementary material for: Prognostic Impacts of Angiotensin-Converting Enzyme Inhibitors and Angiotensin Receptor Blockers in Acute Coronary Syndrome Patients Without Heart Failure
Source: Front Pharmacol. 2022 Apr 5;13:663811. doi: 10.3389/fphar.2022.663811 (PMC9037138; doi:10.3389/fphar.2022.663811)

**Supplementary files**

**Supplementary table 1.** Types and daily dosages of angiotensin converting enzyme inhibitors (ACEI) and angiotensin receptor blockers (ARB) prescribed at discharge.

| **ACEI** | | **Daily dosage (mg)** | | | | |
| --- | --- | --- | --- | --- | --- | --- |
| **Benazepril hydrochloride** | **Subtotal** | 2.5 | 5 | 10 | 20 |  |
|  | **129 (7.1)** | 7 (5.4) | 51 (39.5) | 65 (50.4) | 6 (4.7) |  |
| **Captopril** | **Subtotal** | <12.5 | 12.5-25 | 25-50 | ≥50 |  |
|  | **739 (40.9)** | 308 (41.7) | 308 (41.7) | 107 (14.5) | 16 (2.2) |  |
| **Enalapril maleate** | **Subtotal** | 5 | 10 | 20 |  |  |
|  | **9 (0.5)** | 2 (22.2) | 5 (55.6) | 2 (22.2) |  |  |
| **Fosinopril sodium** | **Subtotal** | 2.5 | 5 | 10 | 15 |  |
|  | **30 (1.7)** | 4 (13.3) | 14 (46.7) | 11 (36.7) | 1 (3.3) |  |
| **Imidapril hydrochloride** | **Subtotal** | 1.25 | 2.5 | 5 | 7.5 | 10 |
|  | **331 (18.3)** | 1 (0.3) | 98 (29.6) | 171 (51.7) | 1 (0.3) | 60 (18.1) |
| **Perindopril tert-butylamine** | **Subtotal** | 1 | 2 | 4 | 8 |  |
|  | **173 (9.6)** | 15 (8.7) | 93 (53.8) | 64 (37.0) | 1 (0.6) |  |
| **Ramipril** | **Subtotal** | 1.25 | 2.5 | 5 | 7.5 | 10 |
|  | **180 (10.0)** | 26 (14.4) | 97 (53.9) | 54 (30.0) | 2 (1.1) | 1 (0.6) |
| **ARB** | | **Daily dosage (mg)** | | | | |
| **Candesartan cilexetil** | **Subtotal** | 4 | 8 |  |  |  |
|  | **8 (0.4)** | 1 (12.5) | 7 (87.5) |  |  |  |
| **Irbesartan** | **Subtotal** | 37.5 | 75 | 150 |  |  |
|  | **29 (1.6)** | 1 (3.4) | 6 (20.7) | 22 (75.9) |  |  |
| **Losartan potassium** | **Subtotal** | 12.5 | 25 | 50 | 100 |  |
|  | **57 (3.2)** | 6 (10.5) | 30 (52.6) | 18 (31.6) | 3 (5.3) |  |
| **Olmesartan Medoxomil** | **Subtotal** | 20 |  |  |  |  |
|  | **2 (0.1)** | 2 (100) |  |  |  |  |
| **Valsartan** | **Subtotal** | 40 | 80 |  |  |  |
|  | **23 (1.3)** | 1 (4.3) | 22 (95.7) |  |  |  |
| **Telmisartan** | **Subtotal** | 20 | 40 | 80 |  |  |
|  | **95 (5.3)** | 10 (10.5) | 38 (40.0) | 47 (49.5) |  |  |

**Supplementary table 2.** Baseline characteristics of diabetic patients in the original dataset stratified by ACEI/ARB medication.

| Variables | All patients (N=749) | No ACEI/ARB (N=181) | ACEI/ARB (N=568) | SMD | P value |
| --- | --- | --- | --- | --- | --- |
| Age, years | 59.4 ± 11.2 | 60.3 ± 11.5 | 59.1 ± 11.1 | 0.101 | 0.23 |
| Male sex, n (%) | 566 (75.6) | 140 (77.3) | 426 (75.0) | 0.055 | 0.52 |
| Hypertension, n (%) | 473 (63.2) | 81 (44.8) | 392 (69.0) | 0.505 | <0.001 |
| Diabetes, n (%) | 749 (100) | 181 (100) | 568 (100) | <0.001 | - |
| History of PCI or CABG, n (%) | 137 (18.3) | 30 (16.6) | 107 (18.8) | 0.059 | 0.49 |
| Peripheral artery diseases, n (%) | 29 (3.9) | 5 (2.8) | 24 (4.2) | 0.080 | 0.37 |
| STEMI, n (%) | 623 (83.2) | 154 (85.1) | 469 (82.6) | 0.068 | 0.43 |
| Hemodynamics |  |  |  |  |  |
| Heart rate, bpm | 75.1 ± 13.0 | 76.8 ± 14.6 | 74.6 ± 12.5 | 0.164 | 0.045 |
| SBP, mmHg | 126.6 ± 17.9 | 119.1 ± 16.5 | 128.9 ± 17.6 | 0.577 | <0.001 |
| DBP, mmHg | 74.2 ± 11.8 | 70.9 ± 11.3 | 75.2 ± 11.8 | 0.368 | <0.001 |
| EF, % | 57.8 ± 4.1 | 57.6 ± 4.0 | 57.8 ± 4.2 | 0.052 | 0.55 |
| Cardiac arrest, n (%) | 13 (1.7) | 4 (2.2) | 9 (1.6) | 0.046 | 0.57 |
| Lab tests |  |  |  |  |  |
| Creatinine, μmoI/L | 78.7 ± 22.9 | 78.5 ± 27.2 | 78.7 ± 21.4 | 0.010 | 0.90 |
| eGFR, ml/min | 87.3 (74.4-101.2) | 87.2 (76.5-102.2) | 87.3 (74.0-100.9) | 0.012 | 0.35 |
| LDL-C, mmol/L | 2.7 ± 0.9 | 2.5 ± 0.8 | 2.7 ± 0.9 | 0.164 | 0.059 |
| hsCRP, mg/L | 5.94 (2.51-11.41) | 6.88 (2.43-11.89) | 5.64 (2.53-11.35) | 0.120 | 0.20 |
| D-dimer, ng/mL | 296 (220-450) | 280 (210-450) | 300 (220-450) | 0.034 | 0.41 |
| Peak cTnI, ng/mL | 1.89 (0.55-6.10) | 1.85 (0.44-6.47) | 1.97 (0.58-5.93) | 0.042 | 0.56 |
| Coronary angiography findings |  |  |  |  |  |
| Culprit lesion, n (%) |  |  |  |  |  |
| LM | 11 (1.5) | 1 (0.6) | 10 (1.8) | 0.190 | 0.39 |
| LAD | 224 (29.9) | 50 (27.6) | 174 (30.6) |  |  |
| LCX | 146 (19.5) | 33 (18.2) | 113 (19.9) |  |  |
| RCA | 365 (48.7) | 97 (53.6) | 268 (47.2) |  |  |
| Bypass grafts | 3 (0.4) | 0 (0) | 3 (0.5) |  |  |
| Multivessel diseases, n (%) | 592 (79.0) | 143 (79.0) | 449 (79.0) | 0.001 | 0.99 |
| Pre-PCI TIMI 0 flow, n (%) | 448 (59.8) | 116 (64.1) | 332 (58.5) | 0.116 | 0.18 |
| Post-PCI TIMI 3 flow, n (%) | 724 (96.7) | 176 (97.2) | 548 (96.5) | 0.044 | 0.62 |
| D2B time ≥ 120 mins, n (%) | 449 (59.9) | 107 (59.1) | 342 (60.2) | 0.022 | 0.79 |
| CR before discharge, n (%) | 322 (43.0) | 76 (42.0) | 246 (43.3) | 0.027 | 0.75 |
| Medications at discharge |  |  |  |  |  |
| Aspirin, n (%) | 743 (99.2) | 179 (98.9) | 564 (99.3) | 0.042 | 0.60 |
| P2Y_12_ inhibitors, n (%) | 744 (99.3) | 177 (97.8) | 567 (99.8) | 0.188 | 0.003 |
| Statins, n (%) | 713 (95.2) | 170 (93.9) | 543 (95.6) | 0.075 | 0.36 |
| β blockers, n (%) | 660 (88.1) | 156 (86.2) | 504 (88.7) | 0.077 | 0.36 |

ACEI = angiotensin-converting enzyme inhibitor, ARB = angiotensin receptor blocker, SMD = standardized mean difference, PCI = percutaneous coronary intervention, CABG = coronary artery bypass grafting, STEMI = ST-segment elevation myocardial infarction, SBP = systolic blood pressure, DBP = diastolic blood pressure, eGFR, estimated glomerular filtration rate, EF = ejection fraction, LDL-C = low-density lipoprotein cholesterol, hsCRP = high-sensitivity C-reactive protein, cTnI = cardiac troponin I, LM = left main, LAD = left anterior descending artery, LCX = left circumflex, RCA = right coronary artery, TIMI flow = Thrombolysis In Myocardial Infarction grade flow, D2B time = door-to-balloon time, CR = complete revascularization.

**Supplementary table 3.** Baseline characteristics of non-diabetic patients in the original dataset stratified by ACEI/ARB medication.

| Variables | All patients (N=1648) | No ACEI/ARB (N=411) | ACEI/ARB (N=1237) | SMD | P value |
| --- | --- | --- | --- | --- | --- |
| Age, years | 57.0 ± 11.7 | 57.2 ± 11.2 | 57.0 ± 11.8 | 0.025 | 0.67 |
| Male sex, n (%) | 1379 (83.7) | 334 (81.3) | 1045 (84.5) | 0.085 | 0.13 |
| Hypertension, n (%) | 953 (57.8) | 156 (38.0) | 797 (64.4) | 0.549 | <0.001 |
| Diabetes, n (%) | 0 (0) | 0 (0) | 0 (0) | <0.001 | - |
| History of PCI or CABG, n (%) | 182 (11.0) | 36 (8.8) | 146 (11.8) | 0.100 | 0.088 |
| Peripheral artery diseases, n (%) | 66 (4.0) | 16 (3.9) | 50 (4.0) | 0.008 | 0.89 |
| STEMI, n (%) | 1426 (86.5) | 349 (84.9) | 1077 (87.1) | 0.062 | 0.27 |
| Hemodynamics |  |  |  |  |  |
| Heart rate, bpm | 74.3 ± 13.6 | 75.9 ± 14.9 | 73.8 ± 13.2 | 0.151 | 0.006 |
| SBP, mmHg | 124.5 ± 17.5 | 119.1 ± 16.9 | 126.3 ± 17.3 | 0.424 | <0.001 |
| DBP, mmHg | 74.9 ± 12.5 | 71.7 ± 11.5 | 76.0 ± 12.7 | 0.361 | <0.001 |
| EF, % | 57.8 ± 4.1 | 57.3 ± 4.1 | 57.9 ± 4.1 | 0.142 | 0.012 |
| Cardiac arrest, n (%) | 45 (2.7) | 15 (3.6) | 30 (2.4) | 0.071 | 0.19 |
| Lab tests |  |  |  |  |  |
| Creatinine, μmoI/L | 80.7 ± 20.2 | 81.4 ± 24.0 | 80.5 ± 18.8 | 0.039 | 0.47 |
| eGFR, ml/min | 86.1 (72.3-98.6) | 85.5 (72.8-99.1) | 86.5 (72.3-98.6) | 0.066 | 0.61 |
| LDL-C, mmol/L | 2.7 ± 0.9 | 2.8 ± 0.9 | 2.7 ± 0.9 | 0.010 | 0.86 |
| hsCRP, mg/L | 5.23 (2.36-11.31) | 6.23 (2.50-11.75) | 5.16 (2.32-11.16) | 0.093 | 0.040 |
| D-dimer, ng/mL | 308 (220-472) | 320 (220-500) | 300 (220-460) | 0.080 | 0.084 |
| Peak cTnI, ng/mL | 2.32 (0.54-7.37) | 2.81 (0.94-9.00) | 2.07 (0.49-6.80) | 0.183 | 0.001 |
| Coronary angiography findings |  |  |  |  |  |
| Culprit lesion, n (%) |  |  |  |  |  |
| LM | 24 (1.5) | 11 (2.7) | 13 (1.1) | 0.296 | <0.001 |
| LAD | 506 (30.7) | 92 (22.4) | 414 (33.5) |  |  |
| LCX | 348 (21.1) | 81 (19.7) | 267 (21.6) |  |  |
| RCA | 767 (46.5) | 226 (55.0) | 541 (43.7) |  |  |
| Bypass grafts | 3 (0.2) | 1 (0.2) | 2 (0.2) |  |  |
| Multivessel diseases, n (%) | 1204 (73.1) | 303 (73.7) | 901 (72.8) | 0.020 | 0.73 |
| Pre-PCI TIMI 0 flow, n (%) | 1056 (64.1) | 278 (67.6) | 778 (62.9) | 0.100 | 0.082 |
| Post-PCI TIMI 3 flow, n (%) | 1608 (97.6) | 401 (97.6) | 1207 (97.6) | 0.001 | 0.99 |
| D2B time ≥ 120 mins, n (%) | 923 (56.0) | 213 (51.8) | 710 (57.4) | 0.112 | 0.049 |
| CR before discharge, n (%) | 761 (46.2) | 183 (44.5) | 578 (46.7) | 0.044 | 0.44 |
| Medications at discharge |  |  |  |  |  |
| Aspirin, n (%) | 1640 (99.5) | 408 (99.3) | 1232 (99.6) | 0.043 | 0.41 |
| P2Y_12_ inhibitors, n (%) | 1640 (99.5) | 406 (98.8) | 1234 (99.8) | 0.115 | 0.014 |
| Statins, n (%) | 1542 (93.6) | 384 (93.4) | 1158 (93.6) | 0.007 | 0.90 |
| β blockers, n (%) | 1400 (85.0) | 309 (75.2) | 1091 (88.2) | 0.341 | <0.001 |

ACEI = angiotensin-converting enzyme inhibitor, ARB = angiotensin receptor blocker, SMD = standardized mean difference, PCI = percutaneous coronary intervention, CABG = coronary artery bypass grafting, STEMI = ST-segment elevation myocardial infarction, SBP = systolic blood pressure, DBP = diastolic blood pressure, EF = ejection fraction, eGFR, estimated glomerular filtration rate, LDL-C = low-density lipoprotein cholesterol, hsCRP = high-sensitivity C-reactive protein, cTnI = cardiac troponin I, LM = left main, LAD = left anterior descending artery, LCX = left circumflex, RCA = right coronary artery, TIMI flow = Thrombolysis In Myocardial Infarction grade flow, D2B time = door-to-balloon time, CR = complete revascularization.

**Supplementary table 4.** Baseline characteristics of hypertensive patients in original dataset stratified by ACEI/ARB medication.

| Variables | All patients (N=1426) | No ACEI/ARB (N=237) | ACEI/ARB (N=1189) | SMD | P value |
| --- | --- | --- | --- | --- | --- |
| Age, years | 59.2 ± 11.3 | 60.5 ± 11.0 | 59.0 ± 11.3 | 0.135 | 0.061 |
| Male sex, n (%) | 1099 (77.1) | 175 (73.8) | 924 (77.7) | 0.090 | 0.20 |
| Hypertension, n (%) | 1426 (100) | 237 (100) | 1189 (100) | <0.001 | - |
| Diabetes, n (%) | 473 (33.2) | 81 (34.2) | 392 (33.0) | 0.026 | 0.72 |
| History of PCI or CABG, n (%) | 224 (15.7) | 35 (14.8) | 189 (15.9) | 0.031 | 0.66 |
| Peripheral artery diseases, n (%) | 72 (5.0) | 14 (5.9) | 58 (4.9) | 0.046 | 0.51 |
| STEMI, n (%) | 1182 (82.9) | 200 (84.4) | 982 (82.6) | 0.048 | 0.50 |
| Hemodynamics |  |  |  |  |  |
| Heart rate, bpm | 74.3 ± 13.2 | 76.1 ± 14.7 | 74.0 ± 12.9 | 0.154 | 0.024 |
| SBP, mmHg | 128.7 ± 18.2 | 122.4 ± 17.9 | 130.0 ± 18.0 | 0.421 | <0.001 |
| DBP, mmHg | 76.4 ± 12.7 | 73.4 ± 12.2 | 77.0 ± 12.7 | 0.288 | <0.001 |
| EF, % | 57.9 ± 4.1 | 57.5 ± 4.2 | 57.9 ± 4.1 | 0.103 | 0.14 |
| Cardiac arrest, n (%) | 32 (2.2) | 9 (3.8) | 23 (1.9) | 0.112 | 0.077 |
| Lab tests |  |  |  |  |  |
| Creatinine, μmoI/L | 81.1 ± 21.9 | 81.6 ± 24.8 | 81.0 ± 21.3 | 0.022 | 0.74 |
| eGFR, ml/min | 84.9 (70.6-97.6) | 84.5 (70.6- 97.2) | 84.9 (70.6-97.7) | 0.095 | 0.59 |
| LDL-C, mmol/L | 2.6 ± 0.9 | 2.6 ± 0.9 | 2.7 ± 0.9 | 0.086 | 0.23 |
| hsCRP, mg/L | 5.69 (2.53-11.39) | 7.87 (2.95-12.32) | 5.46 (2.45-11.24) | 0.229 | <0.001 |
| D-dimer, ng/mL | 320 (220-490) | 340 (230-550) | 316 (220-480) | 0.149 | 0.071 |
| Peak cTnI, ng/mL | 2.08 (0.54-6.71) | 2.62 (0.79-8.91) | 1.94 (0.51-6.09) | 0.204 | 0.008 |
| Coronary angiography findings |  |  |  |  |  |
| Culprit lesion, n (%) |  |  |  |  |  |
| LM | 23 (1.6) | 4 (1.7) | 19 (1.6) | 0.209 | 0.087 |
| LAD | 411 (28.8) | 51 (21.5) | 360 (30.3) |  |  |
| LCX | 312 (21.9) | 53 (22.4) | 259 (21.8) |  |  |
| RCA | 676 (47.4) | 128 (54.0) | 548 (46.1) |  |  |
| Bypass grafts | 4 (0.3) | 1 (0.4) | 3 (0.3) |  |  |
| Multivessel diseases, n (%) | 1128 (79.1) | 189 (79.7) | 939 (79.0) | 0.019 | 0.79 |
| Pre-PCI TIMI 0 flow, n (%) | 883 (61.9) | 157 (66.2) | 726 (61.1) | 0.108 | 0.13 |
| Post-PCI TIMI 3 flow, n (%) | 1385 (97.1) | 230 (97.0) | 1155 (97.1) | 0.006 | 0.94 |
| D2B time ≥ 120 mins, n (%) | 848 (59.5) | 129 (54.4) | 719 (60.5) | 0.122 | 0.084 |
| CR before discharge, n (%) | 605 (42.4) | 101 (42.6) | 504 (42.4) | 0.005 | 0.95 |
| Medications at discharge |  |  |  |  |  |
| Aspirin, n (%) | 1415 (99.2) | 234 (98.7) | 1181 (99.3) | 0.061 | 0.34 |
| P2Y_12_ inhibitors, n (%) | 1419 (99.5) | 232 (97.9) | 1187 (99.8) | 0.184 | <0.001 |
| Statins, n (%) | 1347 (94.5) | 227 (95.8) | 1120 (94.2) | 0.073 | 0.33 |
| β blockers, n (%) | 1246 (87.4) | 198 (83.5) | 1048 (88.1) | 0.132 | 0.052 |

ACEI = angiotensin-converting enzyme inhibitor, ARB = angiotensin receptor blocker, SMD = standardized mean difference, PCI = percutaneous coronary intervention, CABG = coronary artery bypass grafting, STEMI = ST-segment elevation myocardial infarction, SBP = systolic blood pressure, DBP = diastolic blood pressure, EF = ejection fraction, eGFR = estimated glomerular filtration rate, LDL-C = low-density lipoprotein cholesterol, hsCRP = high-sensitivity C-reactive protein, cTnI = cardiac troponin I, LM = left main, LAD = left anterior descending artery, LCX = left circumflex, RCA = right coronary artery, TIMI flow = Thrombolysis In Myocardial Infarction grade flow, D2B time = door-to-balloon time, CR = complete revascularization.

**Supplementary table 5.** Baseline characteristics of non-hypertensive patients in original dataset stratified by ACEI/ARB medication.

| Variables | All patients (N=971) | No ACEI/ARB (N=355) | ACEI/ARB (N=616) | SMD | P value |
| --- | --- | --- | --- | --- | --- |
| Age, years | 55.6 ± 11.7 | 56.6 ± 11.4 | 55.1 ± 11.8 | 0.136 | 0.043 |
| Male sex, n (%) | 846 (87.1) | 299 (84.2) | 547 (88.8) | 0.134 | 0.040 |
| Hypertension, n (%) | 0 (0) | 0 (0) | 0 (0) | <0.001 | - |
| Diabetes, n (%) | 276 (28.4) | 100 (28.2) | 176 (28.6) | 0.009 | 0.89 |
| History of PCI or CABG, n (%) | 95 (9.8) | 31 (8.7) | 64 (10.4) | 0.056 | 0.40 |
| Peripheral artery diseases, n (%) | 23 (2.4) | 7 (2.0) | 16 (2.6) | 0.042 | 0.54 |
| STEMI, n (%) | 867 (89.3) | 303 (85.4) | 564 (91.6) | 0.195 | 0.003 |
| Hemodynamics |  |  |  |  |  |
| Heart rate, bpm | 74.9 ± 13.8 | 76.2 ± 14.8 | 74.1 ± 13.0 | 0.150 | 0.022 |
| SBP, mmHg | 119.9 ± 15.3 | 116.8 ± 15.6 | 121.7 ± 14.8 | 0.317 | <0.001 |
| DBP, mmHg | 72.2 ± 11.3 | 70.1 ± 10.7 | 73.4 ± 11.5 | 0.295 | <0.001 |
| EF, % | 57.7 ± 4.1 | 57.4 ± 4.1 | 57.8 ± 4.2 | 0.109 | 0.10 |
| Cardiac arrest, n (%) | 26 (2.7) | 10 (2.8) | 16 (2.6) | 0.014 | 0.84 |
| Lab tests |  |  |  |  |  |
| Creatinine, μmoI/L | 78.5 ± 19.9 | 79.7 ± 25.2 | 77.8 ± 16.0 | 0.091 | 0.15 |
| eGFR, ml/min | 89.1 (78.0-102.5) | 87.5 (76.3-101.9) | 89.7 (77.5-103.3) | 0.069 | 0.16 |
| LDL-C, mmol/L | 2.8 ± 0.9 | 2.8 ± 0.9 | 2.9 ± 1.0 | 0.106 | 0.11 |
| hsCRP, mg/L | 5.06 (2.14-11.22) | 5.23 (2.20-11.48) | 5.00 (2.08-11.12) | 0.040 | 0.39 |
| D-dimer, ng/mL | 280 (210-420) | 290 (210-430) | 270 (210-410) | 0.036 | 0.28 |
| Peak cTnI, ng/mL | 2.25 (0.55-7.43) | 2.34 (0.67-8.25) | 2.22 (0.51-7.15) | 0.038 | 0.49 |
| Coronary angiography findings |  |  |  |  |  |
| Culprit lesion, n (%) |  |  |  |  |  |
| LM | 12 (1.2) | 8 (2.3) | 4 (0.6) | 0.322 | <0.001 |
| LAD | 319 (32.9) | 91 (25.6) | 228 (37.0) |  |  |
| LCX | 182 (18.7) | 61 (17.2) | 121 (19.6) |  |  |
| RCA | 456 (47.0) | 195 (54.9) | 261 (42.4) |  |  |
| Bypass grafts | 2 (0.2) | 0 (0.0) | 2 (0.3) |  |  |
| Multivessel diseases, n (%) | 668 (68.8) | 257 (72.4) | 411 (66.7) | 0.124 | 0.066 |
| Pre-PCI TIMI 0 flow, n (%) | 621 (64.0) | 237 (66.8) | 384 (62.3) | 0.093 | 0.17 |
| Post-PCI TIMI 3 flow, n (%) | 947 (97.5) | 347 (97.7) | 600 (97.4) | 0.022 | 0.74 |
| D2B time ≥ 120 mins, n (%) | 524 (54.0) | 191 (53.8) | 333 (54.1) | 0.005 | 0.94 |
| CR before discharge, n (%) | 478 (49.2) | 158 (44.5) | 320 (51.9) | 0.149 | 0.026 |
| Medications at discharge |  |  |  |  |  |
| Aspirin, n (%) | 968 (99.7) | 353 (99.4) | 615 (99.8) | 0.067 | 0.28 |
| P2Y_12_ inhibitors, n (%) | 965 (99.4) | 351 (98.9) | 614 (99.7) | 0.095 | 0.12 |
| Statins, n (%) | 908 (93.5) | 327 (92.1) | 581 (94.3) | 0.088 | 0.18 |
| β blockers, n (%) | 814 (83.8) | 267 (75.2) | 547 (88.8) | 0.359 | <0.001 |

ACEI = angiotensin-converting enzyme inhibitor, ARB = angiotensin receptor blocker, SMD = standardized mean difference, PCI = percutaneous coronary intervention, CABG = coronary artery bypass grafting, STEMI = ST-segment elevation myocardial infarction, SBP = systolic blood pressure, DBP = diastolic blood pressure, EF = ejection fraction, eGFR, estimated glomerular filtration rate, LDL-C = low-density lipoprotein cholesterol, hsCRP = high-sensitivity C-reactive protein, cTnI = cardiac troponin I, LM = left main, LAD = left anterior descending artery, LCX = left circumflex, RCA = right coronary artery, TIMI flow = Thrombolysis In Myocardial Infarction grade flow, D2B time = door-to-balloon time, CR = complete revascularization.

**Supplementary table 6.** Baseline characteristics of anterior infarction patients in original dataset by ACEI/ARB medication.

| Variables | All patients (N=765) | No ACEI/ARB (N=154) | ACEI/ARB (N=611) | SMD | P value |
| --- | --- | --- | --- | --- | --- |
| Age, years | 56.6 ± 11.6 | 56.7 ± 11.0 | 56.6 ± 11.8 | 0.016 | 0.87 |
| Male sex, n (%) | 623 (81.4) | 121 (78.6) | 502 (82.2) | 0.090 | 0.31 |
| Hypertension, n (%) | 434 (56.7) | 55 (35.7) | 379 (62.0) | 0.546 | <0.001 |
| Diabetes, n (%) | 235 (30.7) | 51 (33.1) | 184 (30.1) | 0.065 | 0.47 |
| History of PCI or CABG, n (%) | 101 (13.2) | 18 (11.7) | 83 (13.6) | 0.057 | 0.53 |
| Peripheral artery diseases, n (%) | 29 (3.8) | 9 (5.8) | 20 (3.3) | 0.123 | 0.14 |
| STEMI, n (%) | 619 (80.9) | 122 (79.2) | 497 (81.3) | 0.053 | 0.55 |
| Hemodynamics |  |  |  |  |  |
| Heart rate, bpm | 73.9 ± 11.8 | 74.6 ± 11.7 | 73.7 ± 11.9 | 0.074 | 0.41 |
| SBP, mmHg | 127.4 ± 7.2 | 121.5 ± 16.2 | 128.9 ± 17.1 | 0.447 | <0.001 |
| DBP, mmHg | 77.5 ± 12.5 | 74.1 ± 12.4 | 78.4 ± 12.4 | 0.343 | <0.001 |
| EF, % | 57.1 ± 4.5 | 56.6 ± 4.5 | 57.2 ± 4.4 | 0.146 | 0.10 |
| Cardiac arrest, n (%) | 11 (1.4) | 4 (2.6) | 7 (1.1) | 0.107 | 0.18 |
| Lab tests |  |  |  |  |  |
| Creatinine, μmoI/L | 78.4 ± 19.8 | 77.9 ± 23.9 | 78.5 ± 18.6 | 0.028 | 0.74 |
| eGFR, ml/min | 88.6 (75.3-101.6) | 87.7(77.2-102.6) | 88.8 (75.1-101.1) | 0.089 | 0.68 |
| LDL-C, mmol/L | 2.7 ± 0.9 | 2.6 ± 0.8 | 2.8 ± 1.0 | 0.232 | 0.013 |
| hsCRP, mg/L | 4.94 (2.13-10.92) | 4.81 (2.05-11.69) | 4.96 (2.13-10.67) | 0.056 | 0.49 |
| D-dimer, ng/mL | 280 (220-410) | 274 (200-420) | 280 (220-410) | 0.094 | 0.56 |
| Peak cTnI, ng/mL | 2.05 (0.51-6.08) | 2.45 (0.64-7.89) | 1.95 (0.47-5.88) | 0.116 | 0.14 |
| Coronary angiography findings |  |  |  |  |  |
| Culprit lesion, n (%) |  |  |  |  |  |
| LM | 35 (4.6) | 12 (7.8) | 23 (3.8) | 0.173 | 0.033 |
| LAD | 730 (95.4) | 142 (92.2) | 588 (96.2) |  |  |
| LCX | 0 (0) | 0 (0) | 0 (0) |  |  |
| RCA | 0 (0) | 0 (0) | 0 (0) |  |  |
| Bypass grafts | 0 (0) | 0 (0) | 0 (0) |  |  |
| Multivessel diseases, n (%) | 476 (62.2) | 92 (59.7) | 384 (62.8) | 0.064 | 0.48 |
| Pre-PCI TIMI 0 flow, n (%) | 374 (48.9) | 85 (55.2) | 289 (47.3) | 0.158 | 0.080 |
| Post-PCI TIMI 3 flow, n (%) | 741 (96.9) | 149 (96.8) | 592 (96.9) | 0.008 | 0.93 |
| D2B time ≥ 120 mins, n (%) | 463 (60.5) | 94 (61.0) | 369 (60.4) | 0.013 | 0.88 |
| CR before discharge, n (%) | 416 (54.4) | 88 (57.1) | 328 (53.7) | 0.070 | 0.44 |
| Medications at discharge |  |  |  |  |  |
| Aspirin, n (%) | 761 (99.5) | 153 (99.4) | 608 (99.5) | 0.021 | 0.81 |
| P2Y_12_ inhibitors, n (%) | 762 (99.6) | 152 (98.7) | 610 (99.8) | 0.134 | 0.044 |
| Statins, n (%) | 720 (94.1) | 145 (94.2) | 575 (94.1) | 0.002 | 0.98 |
| β blockers, n (%) | 717 (93.7) | 138 (89.6) | 579 (94.8) | 0.193 | 0.018 |

ACEI = angiotensin-converting enzyme inhibitor, ARB = angiotensin receptor blocker, SMD = standardized mean difference, PCI = percutaneous coronary intervention, CABG = coronary artery bypass grafting, STEMI = ST-segment elevation myocardial infarction, SBP = systolic blood pressure, DBP = diastolic blood pressure, EF = ejection fraction, eGFR, estimated glomerular filtration rate, LDL-C = low-density lipoprotein cholesterol, hsCRP = high-sensitivity C-reactive protein, cTnI = cardiac troponin I, LM = left main, LAD = left anterior descending artery, LCX = left circumflex, RCA = right coronary artery, TIMI flow = Thrombolysis In Myocardial Infarction grade flow, D2B time = door-to-balloon time, CR = complete revascularization.

**Supplementary table 7.** Baseline characteristics of non-anterior infarction patients in original dataset by ACEI/ARB medication.

| Variables | All patients (N=1632) | No ACEI/ARB (N=438) | ACEI/ARB (N=1194) | SMD | P value |
| --- | --- | --- | --- | --- | --- |
| Age, years | 58.3 ± 11.5 | 58.7 ± 11.5 | 58.2 ± 11.5 | 0.042 | 0.46 |
| Male sex, n (%) | 1322 (81.0) | 353 (80.6) | 969 (81.2) | 0.014 | 0.80 |
| Hypertension, n (%) | 992 (60.8) | 182 (41.6) | 810 (67.8) | 0.547 | <0.001 |
| Diabetes, n (%) | 514 (31.5) | 130 (29.7) | 384 (32.2) | 0.054 | 0.34 |
| History of PCI or CABG, n (%) | 218 (13.4) | 48 (11.0) | 170 (14.2) | 0.099 | 0.084 |
| Peripheral artery diseases, n (%) | 66 (4.0) | 12 (2.7) | 54 (4.5) | 0.095 | 0.11 |
| STEMI, n (%) | 1430 (87.6) | 381 (87.0) | 1049 (87.9) | 0.026 | 0.64 |
| Hemodynamics |  |  |  |  |  |
| Heart rate, bpm | 74.9 ± 14.1 | 76.8 ± 15.7 | 74.2 ± 13.5 | 0.174 | 0.001 |
| SBP, mmHg | 124.1 ± 17.8 | 118.2 ± 16.9 | 126.2 ± 17.6 | 0.464 | <0.001 |
| DBP, mmHg | 73.4 ± 12.0 | 70.5 ± 10.9 | 74.4 ± 12.2 | 0.339 | <0.001 |
| EF, % | 58.1 ± 3.9 | 57.7 ± 3.9 | 58.2 ± 3.9 | 0.131 | 0.019 |
| Cardiac arrest, n (%) | 47 (2.9) | 15 (3.4) | 32 (2.7) | 0.043 | 0.43 |
| Lab tests |  |  |  |  |  |
| Creatinine, μmoI/L | 80.9 ± 21.7 | 81.4 ± 25.4 | 80.7 ± 20.1 | 0.030 | 0.57 |
| eGFR, ml/min | 85.5 (72.0-98.6) | 85.5 (72.2-99.5) | 85.5 (71.9-98.3) | 0.014 | 0.92 |
| LDL-C, mmol/L | 2.7 ± 0.9 | 2.7 ± 0.9 | 2.7 ± 0.9 | 0.034 | 0.54 |
| hsCRP, mg/L | 5.69 (2.53-11.51) | 6.93 (2.71-11.81) | 5.45 (2.46-11.41) | 0.103 | 0.032 |
| D-dimer, ng/mL | 316 (220-490) | 321 (220-510) | 310 (220-490) | 0.070 | 0.25 |
| Peak cTnI, ng/mL | 2.24 (0.59-7.44) | 2.46 (0.77-8.49) | 2.13 (0.54-7.12) | 0.111 | 0.068 |
| Coronary angiography findings |  |  |  |  |  |
| Culprit lesion, n (%) |  |  |  |  |  |
| LM | 0 (0) | 0 (0) | 0 (0) | 0.134 | 0.063 |
| LAD | 0 (0) | 0 (0) | 0 (0) |  |  |
| LCX | 494 (30.3) | 114 (26.0) | 380 (31.8) |  |  |
| RCA | 1132 (69.4) | 323 (73.7) | 809 (67.8) |  |  |
| Bypass grafts | 6 (0.4) | 1 (0.2) | 5 (0.4) |  |  |
| Multivessel diseases, n (%) | 1320 (80.9) | 354 (80.8) | 966 (80.9) | 0.002 | 0.97 |
| Pre-PCI TIMI 0 flow, n (%) | 1130 (69.2) | 309 (70.5) | 821 (68.8) | 0.039 | 0.49 |
| Post-PCI TIMI 3 flow, n (%) | 1591 (97.5) | 428 (97.7) | 1163 (97.4) | 0.020 | 0.72 |
| D2B time ≥ 120 mins, n (%) | 909 (55.7) | 226 (51.6) | 683 (57.2) | 0.113 | 0.043 |
| CR before discharge, n (%) | 667 (40.9) | 171 (39.0) | 496 (41.5) | 0.051 | 0.36 |
| Medications at discharge |  |  |  |  |  |
| Aspirin, n (%) | 1622 (99.4) | 434 (99.1) | 1188 (99.5) | 0.049 | 0.35 |
| P2Y_12_ inhibitors, n (%) | 1622 (99.4) | 431 (98.4) | 1191 (99.7) | 0.141 | 0.002 |
| Statins, n (%) | 1535 (94.1) | 409 (93.4) | 1126 (94.3) | 0.039 | 0.48 |
| β blockers, n (%) | 1343 (82.3) | 327 (74.7) | 1016 (85.1) | 0.262 | <0.001 |

ACEI = angiotensin-converting enzyme inhibitor, ARB = angiotensin receptor blocker, SMD = standardized mean difference, PCI = percutaneous coronary intervention, CABG = coronary artery bypass grafting, STEMI = ST-segment elevation myocardial infarction, SBP = systolic blood pressure, DBP = diastolic blood pressure, EF = ejection fraction, eGFR, estimated glomerular filtration rate, LDL-C = low-density lipoprotein cholesterol, hsCRP = high-sensitivity C-reactive protein, cTnI = cardiac troponin I, LM = left main, LAD = left anterior descending artery, LCX = left circumflex, RCA = right coronary artery, TIMI flow = Thrombolysis In Myocardial Infarction grade flow, D2B time = door-to-balloon time, CR = complete revascularization.

**Supplementary table 8.** Baseline characteristics of in the original dataset stratified by the medication of ACEI.

| Variables | All patients (N=2183) | No ACEI (N=592) | ACEI (N=1591) | SMD | P value |
| --- | --- | --- | --- | --- | --- |
| Age, years | 56.7 ± 11.5 | 58.2 ± 11.4 | 57.5 ±11.5 | 0.059 | 0.23 |
| Male sex, n (%) | 1780 (81.5) | 474 (80.1) | 1306 (82.1) | 0.052 | 0.28 |
| Hypertension, n (%) | 1247 (57.1) | 237 (40.0) | 1010 (63.5) | 0.483 | <0.001 |
| Diabetes, n (%) | 671 (30.7) | 181 (30.6) | 490 (30.8) | 0.005 | 0.92 |
| History of PCI or CABG, n (%) | 276 (12.6) | 66 (11.1) | 210 (13.2) | 0.063 | 0.20 |
| Peripheral artery diseases, n (%) | 91 (4.2) | 21 (3.5) | 70 (4.4) | 0.044 | 0.38 |
| STEMI, n (%) | 1884 (86.3) | 503 (85.0) | 1381 (86.8) | 0.053 | 0.27 |
| Hemodynamics |  |  |  |  |  |
| Heart rate, bpm | 74.7 ± 13.5 | 76.2 ± 14.8 | 74.1 ± 12.9 | 0.151 | 0.001 |
| SBP, mmHg | 124.3 ± 17.1 | 119.1 ± 16.8 | 126.3 ± 16.9 | 0.428 | <0.001 |
| DBP, mmHg | 74.5 ± 12.2 | 71.5 ± 11.4 | 75.6 ± 12.2 | 0.349 | <0.001 |
| EF, % | 57.7 ± 4.1 | 57.4 ± 4.1 | 57.8 ± 4.1 | 0.090 | 0.061 |
| Cardiac arrest, n (%) | 55 (2.5) | 19 (3.2) | 36 (2.3) | 0.058 | 0.21 |
| Lab tests |  |  |  |  |  |
| Creatinine, μmoI/L | 79.6 ± 20.9 | 80.5 ± 25.0 | 79.3 ± 19.1 | 0.054 | 0.24 |
| eGFR, ml/min | 86.9 (74.0-100.3) | 86.0 (73.7-100.4) | 87.3 (74.0-100.3) | 0.064 | 0.50 |
| LDL-C, mmol/L | 2.7 ± 0.9 | 2.7 ± 0.9 | 2.7 ± 0.9 | 0.055 | 0.26 |
| hsCRP, mg/L | 5.46 (2.40-11.37) | 6.43 (2.50-11.79) | 5.28 (2.37-11.24) | 0.094 | 0.019 |
| D-dimer, ng/mL | 300 (220-460) | 310 (220-490) | 300 (220-451) | 0.055 | 0.29 |
| Peak cTnI, ng/mL | 2.20 (0.57-7.16) | 2.45 (0.70-8.46) | 2.09 (0.53-6.68) | 0.105 | 0.031 |
| Coronary angiography findings |  |  |  |  |  |
| Culprit lesion, n (%) |  |  |  |  |  |
| LM | 35 (1.6) | 12 (2.0) | 23 (1.4) | 0.221 | <0.001 |
| LAD | 661 (30.3) | 142 (24.0) | 519 (32.6) |  |  |
| LCX | 441 (20.2) | 114 (19.3) | 327 (20.6) |  |  |
| RCA | 1040 (47.6) | 323 (54.6) | 717 (45.1) |  |  |
| Bypass grafts | 6 (0.3) | 1 (0.2) | 5 (0.3) |  |  |
| Multivessel diseases, n (%) | 1638 (75.0) | 446 (75.3) | 1192 (74.9) | 0.010 | 0.84 |
| Pre-PCI TIMI 0 flow, n (%) | 1381 (63.3) | 394 (66.6) | 987 (62.0) | 0.094 | 0.052 |
| Post-PCI TIMI 3 flow, n (%) | 2124 (97.3) | 577 (97.5) | 1547 (97.2) | 0.014 | 0.77 |
| D2B time ≥ 120 mins, n (%) | 1227 (56.2) | 320 (54.1) | 907 (57.0) | 0.059 | 0.22 |
| CR before discharge, n (%) | 981 (44.9) | 259 (43.8) | 722 (45.4) | 0.033 | 0.50 |
| Medications at discharge |  |  |  |  |  |
| Aspirin, n (%) | 2170 (99.4) | 587 (99.2) | 1583 (99.5) | 0.042 | 0.36 |
| P2Y_12_ inhibitors, n (%) | 2171 (99.5) | 583 (98.5) | 1588 (99.8) | 0.145 | <0.001 |
| Statins, n (%) | 2050 (93.9) | 554 (93.6) | 1496 (94.0) | 0.019 | 0.70 |
| β blockers, n (%) | 1866 (85.5) | 465 (78.5) | 1401 (88.1) | 0.257 | <0.001 |

ACEI = angiotensin-converting enzyme inhibitor, SMD = standardized mean difference, PCI = percutaneous coronary intervention, CABG = coronary artery bypass grafting, STEMI = ST-segment elevation myocardial infarction, SBP = systolic blood pressure, DBP = diastolic blood pressure, EF = ejection fraction, eGFR, estimated glomerular filtration rate, LDL-C = low-density lipoprotein cholesterol, hsCRP = high-sensitivity C-reactive protein, cTnI = cardiac troponin I, LM = left main, LAD = left anterior descending artery, LCX = left circumflex, RCA = right coronary artery, TIMI flow = Thrombolysis In Myocardial Infarction grade flow, D2B time = door-to-balloon time, CR = complete revascularization.

**Supplementary table 9.** Baseline characteristics of in the original dataset stratified by the medication of ARB.

| Variables | All patients (N=806) | No ARB (N=592) | ARB (N=214) | SMD | P value |
| --- | --- | --- | --- | --- | --- |
| Age, years | 58.3 ± 11.6 | 58.2 ± 11.4 | 58.7 ± 12.4 | 0.045 | 0.57 |
| Male sex, n (%) | 639 (79.3) | 474 (80.1) | 165 (77.1) | 0.072 | 0.36 |
| Hypertension, n (%) | 416 (51.6) | 237 (40.0) | 179 (83.6) | 1.005 | <0.001 |
| Diabetes, n (%) | 259 (32.1) | 181 (30.6) | 78 (36.4) | 0.125 | 0.11 |
| History of PCI or CABG, n (%) | 109 (13.5) | 66 (11.1) | 43 (20.1) | 0.248 | 0.001 |
| Peripheral artery diseases, n (%) | 25 (3.1) | 21 (3.5) | 4 (1.9) | 0.104 | 0.22 |
| STEMI, n (%) | 668 (82.9) | 503 (85.0) | 165 (77.1) | 0.202 | 0.009 |
| Hemodynamics |  |  |  |  |  |
| Heart rate, bpm | 75.5 ± 14.4 | 76.2 ± 14.8 | 73.7 ± 13.3 | 0.180 | 0.028 |
| SBP, mmHg | 122.9 ± 18.9 | 119.1 ± 16.8 | 133.6 ± 20.4 | 0.777 | <0.001 |
| DBP, mmHg | 73.0 ± 12.2 | 71.5 ± 11.4 | 77.2 ± 13.5 | 0.461 | <0.001 |
| EF, % | 57.7 ± 4.1 | 57.4 ± 4.1 | 58.6 ± 4.1 | 0.295 | <0.001 |
| Cardiac arrest, n (%) | 22 (2.7) | 19 (3.2) | 3 (1.4) | 0.121 | 0.16 |
| Lab tests |  |  |  |  |  |
| Creatinine, μmoI/L | 81.6 ± 24.5 | 80.5 ± 25.0 | 84.9 ± 22.8 | 0.186 | 0.023 |
| eGFR, ml/min | 85.3 (71.2-98.4) | 86.0 (73.7-100.4) | 81.9 (65.0-94.4) | 0.201 | <0.001 |
| LDL-C, mmol/L | 2.7 ± 0.9 | 2.7 ± 0.9 | 2.6 ± 0.9 | 0.066 | 0.41 |
| hsCRP, mg/L | 5.97 (2.45-11.58) | 6.43 (2.50-11.79) | 5.35 (2.42-11.13) | 0.146 | 0.12 |
| D-dimer, ng/mL | 310 (210-490) | 310 (220-490) | 300 (210-508) | 0.034 | 0.83 |
| Peak cTnI, ng/mL | 2.31 (0.59-7.69) | 2.45 (0.70-8.46) | 1.58 (0.40-5.63) | 0.187 | 0.011 |
| Coronary angiography findings |  |  |  |  |  |
| Culprit lesion, n (%) |  |  |  |  |  |
| LM | 12 (1.5) | 12 (2.0) | 0 (0.0) | 0.335 | 0.004 |
| LAD | 211 (26.2) | 142 (24.0) | 69 (32.2) |  |  |
| LCX | 167 (20.7) | 114 (19.3) | 53 (24.8) |  |  |
| RCA | 415 (51.5) | 323 (54.6) | 92 (43.0) |  |  |
| Bypass grafts | 1 (0.1) | 1 (0.2) | 0 (0.0) |  |  |
| Multivessel diseases, n (%) | 604 (74.9) | 446 (75.3) | 158 (73.8) | 0.035 | 0.66 |
| Pre-PCI TIMI 0 flow, n (%) | 517 (64.1) | 394 (66.6) | 123 (57.5) | 0.188 | 0.018 |
| Post-PCI TIMI 3 flow, n (%) | 785 (97.4) | 577 (97.5) | 208 (97.2) | 0.017 | 0.83 |
| D2B time ≥ 120 mins, n (%) | 465 (57.7) | 320 (54.1) | 145 (67.8) | 0.284 | <0.001 |
| CR before discharge, n (%) | 361 (44.8) | 259 (43.8) | 102 (47.7) | 0.079 | 0.32 |
| Medications at discharge |  |  |  |  |  |
| Aspirin, n (%) | 800 (99.3) | 587 (99.2) | 213 (99.5) | 0.047 | 0.58 |
| P2Y_12_ inhibitors, n (%) | 796 (98.8) | 583 (98.5) | 213 (99.5) | 0.106 | 0.23 |
| Statins, n (%) | 759 (94.2) | 554 (93.6) | 205 (95.8) | 0.099 | 0.24 |
| β blockers, n (%) | 659 (81.8) | 465 (78.5) | 194 (90.7) | 0.340 | <0.001 |

ARB = angiotensin receptor blocker, SMD = standardized mean difference, PCI = percutaneous coronary intervention, CABG = coronary artery bypass grafting, STEMI = ST-segment elevation myocardial infarction, SBP = systolic blood pressure, DBP = diastolic blood pressure, EF = ejection fraction, eGFR, estimated glomerular filtration rate, LDL-C = low-density lipoprotein cholesterol, hsCRP = high-sensitivity C-reactive protein, cTnI = cardiac troponin I, LM = left main, LAD = left anterior descending artery, LCX = left circumflex, RCA = right coronary artery, TIMI flow = Thrombolysis In Myocardial Infarction grade flow, D2B time = door-to-balloon time, CR = complete revascularization.

**Supplementary table 10.** Baseline characteristics of diabetic patients in the PSM dataset stratified by ACEI/ARB medication.

| Variables | All patients (N=334) | No ACEI/ARB (N=167) | ACEI/ARB (N=167) | SMD | P value |
| --- | --- | --- | --- | --- | --- |
| Age, years | 60.0 ± 10.9 | 59.6 ± 11.2 | 60.3 ± 10.6 | 0.064 | 0.56 |
| Male sex, n (%) | 258 (77.2) | 128 (76.6) | 130 (77.8) | 0.029 | 0.79 |
| Hypertension, n (%) | 161 (48.2) | 78 (46.7) | 83 (49.7) | 0.060 | 0.58 |
| Diabetes, n (%) | 334 (100) | 167 (100) | 167 (100) | <0.001 | - |
| History of PCI or CABG, n (%) | 46 (13.8) | 27 (16.2) | 19 (11.4) | 0.139 | 0.20 |
| Peripheral artery diseases, n (%) | 9 (2.7) | 5 (3.0) | 4 (2.4) | 0.037 | 0.74 |
| STEMI, n (%) | 288 (86.2) | 142 (85.0) | 146 (87.4) | 0.070 | 0.53 |
| Hemodynamics |  |  |  |  |  |
| Heart rate, bpm | 76.3 ± 13.2 | 76.2 ± 13.8 | 76.4 ± 12.5 | 0.017 | 0.88 |
| SBP, mmHg | 120.4 ± 15.6 | 120.3 ± 16.3 | 120.5 ± 15.0 | 0.016 | 0.88 |
| DBP, mmHg | 70.8 ± 10.7 | 71.4 ± 11.2 | 70.3 ± 10.1 | 0.098 | 0.37 |
| EF, % | 57.6 ± 4.2 | 57.8 ± 3.9 | 57.3 ± 4.5 | 0.120 | 0.27 |
| Cardiac arrest, n (%) | 5 (1.5) | 2 (1.2) | 3 (1.8) | 0.049 | 0.65 |
| Lab tests |  |  |  |  |  |
| Creatinine, μmoI/L | 79.2 ± 22.5 | 77.1 ± 21.1 | 81.3 ± 23.7 | 0.188 | 0.086 |
| eGFR, ml/min | 86.7 (72.6-100.8) | 87.5 (76.5-102.6) | 85.7 (67.6-98.6) | 0.005 | 0.025 |
| LDL-C, mmol/L | 2.5 ± 0.8 | 2.5 ± 0.8 | 2.5 ±0.8 | 0.003 | 0.98 |
| hsCRP, mg/L | 6.52 (2.50-12.09) | 6.20 (2.22-11.48) | 6.63 (2.71-12.36) | 0.100 | 0.28 |
| D-dimer, ng/mL | 289 (218-450) | 280 (210-440) | 290 (220-470) | 0.019 | 0.92 |
| Peak cTnI, ng/mL | 1.85 (0.59-4.84) | 1.90 (0.44-6.50) | 1.84 (0.63-3.57) | 0.045 | 0.55 |
| Coronary angiography findings |  |  |  |  |  |
| Culprit lesion, n (%) |  |  |  |  |  |
| LM | 1 (0.3) | 1 (0.6) | 0 (0) | 0.127 | 0.72 |
| LAD | 92 (27.5) | 48 (28.7) | 44 (26.3) |  |  |
| LCX | 67 (20.1) | 32 (19.2) | 35 (21.0) |  |  |
| RCA | 174 (52.1) | 86 (51.5) | 88 (52.7) |  |  |
| Bypass grafts | 0 (0) | 0 (0) | 0 (0) |  |  |
| Multivessel diseases, n (%) | 259 (77.5) | 131 (78.4) | 128 (76.6) | 0.043 | 0.69 |
| Pre-PCI TIMI 0 flow, n (%) | 221 (66.2) | 104 (62.3) | 117 (70.1) | 0.165 | 0.13 |
| Post-PCI TIMI 3 flow, n (%) | 324 (97.0) | 162 (97.0) | 162 (97.0) | <0.001 | 1.00 |
| D2B time ≥ 120 mins, n (%) | 196 (58.7) | 97 (58.1) | 99 (59.3) | 0.024 | 0.82 |
| CR before discharge, n (%) | 152 (45.5) | 71 (42.5) | 81 (48.5) | 0.120 | 0.27 |
| Medications at discharge |  |  |  |  |  |
| Aspirin, n (%) | 331 (99.1) | 166 (99.4) | 165 (98.8) | 0.064 | 0.56 |
| P2Y_12_ inhibitors, n (%) | 331 (99.1) | 165 (98.8) | 166 (99.4) | 0.064 | 0.56 |
| Statins, n (%) | 312 (93.4) | 158 (94.6) | 154 (92.2) | 0.097 | 0.38 |
| β blockers, n (%) | 294 (88.0) | 146 (87.4) | 148 (88.6) | 0.037 | 0.74 |

PSM = propensity score matching, ACEI = angiotensin-converting enzyme inhibitor, ARB = angiotensin receptor blocker, SMD = standardized mean difference, PCI = percutaneous coronary intervention, CABG = coronary artery bypass grafting, STEMI = ST-segment elevation myocardial infarction, SBP = systolic blood pressure, DBP = diastolic blood pressure, EF = ejection fraction, eGFR, estimated glomerular filtration rate, LDL-C = low-density lipoprotein cholesterol, hsCRP = high-sensitivity C-reactive protein, cTnI = cardiac troponin I, LM = left main, LAD = left anterior descending artery, LCX = left circumflex, RCA = right coronary artery, TIMI flow = Thrombolysis In Myocardial Infarction grade flow, D2B time = door-to-balloon time, CR = complete revascularization.

**Supplementary table 11.** Baseline characteristics of non-diabetic patients in the PSM dataset stratified by ACEI/ARB medication.

| Variables | All patients (N=746) | No ACEI/ARB (N=373) | ACEI/ARB (N=373) | SMD | P value |
| --- | --- | --- | --- | --- | --- |
| Age, years | 57.3 ± 11.5 | 57.2 ± 11.1 | 57.4 ± 11.9 | 0.018 | 0.81 |
| Male sex, n (%) | 598 (80.2) | 302 (81.0) | 296 (79.4) | 0.040 | 0.58 |
| Hypertension, n (%) | 318 (42.6) | 154 (41.3) | 164 (44.0) | 0.054 | 0.46 |
| Diabetes, n (%) | 0 (0) | 0 (0) | 0 (0) | <0.001 | - |
| History of PCI or CABG, n (%) | 52 (7.0) | 30 (8.0) | 22 (5.9) | 0.084 | 0.25 |
| Peripheral artery diseases, n (%) | 31 (4.2) | 14 (3.8) | 17 (4.6) | 0.040 | 0.58 |
| STEMI, n (%) | 665 (89.1) | 324 (86.9) | 341 (91.4) | 0.147 | 0.045 |
| Hemodynamics |  |  |  |  |  |
| Heart rate, bpm | 76.0 ± 14.3 | 75.4 ± 14.2 | 76.6 ± 14.4 | 0.082 | 0.27 |
| SBP, mmHg | 119.6 ± 16.6 | 120.1 ± 17.0 | 119.2 ± 16.3 | 0.051 | 0.49 |
| DBP, mmHg | 71.4 ± 11.7 | 72.4 ± 11.5 | 70.4 ± 11.9 | 0.170 | 0.021 |
| EF, % | 57.2 ± 4.2 | 57.3 ± 4.2 | 57.1 ± 4.1 | 0.060 | 0.41 |
| Cardiac arrest, n (%) | 25 (3.4) | 12 (3.2) | 13 (3.5) | 0.015 | 0.84 |
| Lab tests |  |  |  |  |  |
| Creatinine, μmoI/L | 81.1 ± 23.0 | 80.7 ± 23.9 | 81.4 ± 22.1 | 0.030 | 0.69 |
| eGFR, ml/min | 85.3 (70.8-99.0) | 85.7 (73.3-99.5) | 84.6 (68.1-98.3) | 0.129 | 0.14 |
| LDL-C, mmol/L | 2.8 ± 0.9 | 2.8 ± 0.9 | 2.8 ± 0.9 | 0.012 | 0.87 |
| hsCRP, mg/L | 6.56 (2.60-11.75) | 5.96 (2.45-11.73) | 7.14 (2.96-11.75) | 0.127 | 0.29 |
| D-dimer, ng/mL | 320 (210-490) | 320 (220-490) | 320 (220-490) | 0.022 | 0.51 |
| Peak cTnI, ng/mL | 2.62 (0.94-7.54) | 2.62 (0.93-8.85) | 2.59 (0.96-6.29) | 0.012 | 0.50 |
| Coronary angiography findings |  |  |  |  |  |
| Culprit lesion, n (%) |  |  |  |  |  |
| LM | 13 (1.7) | 7 (1.9) | 6 (1.6) | 0.047 | 0.98 |
| LAD | 175 (23.5) | 90 (24.1) | 85 (22.8) |  |  |
| LCX | 152 (20.4) | 77 (20.6) | 75 (20.1) |  |  |
| RCA | 404 (54.2) | 198 (53.1) | 206 (55.2) |  |  |
| Bypass grafts | 2 (0.3) | 1 (0.3) | 1 (0.3) |  |  |
| Multivessel diseases, n (%) | 553 (74.1) | 275 (73.7) | 278 (74.5) | 0.018 | 0.80 |
| Pre-PCI TIMI 0 flow, n (%) | 524 (70.2) | 252 (67.6) | 272 (72.9) | 0.117 | 0.11 |
| Post-PCI TIMI 3 flow, n (%) | 726 (97.3) | 364 (97.6) | 362 (97.1) | 0.033 | 0.65 |
| D2B time ≥ 120 mins, n (%) | 386 (51.7) | 192 (51.5) | 194 (52.0) | 0.011 | 0.88 |
| CR before discharge, n (%) | 327 (43.8) | 165 (44.2) | 162 (43.4) | 0.016 | 0.82 |
| Medications at discharge |  |  |  |  |  |
| Aspirin, n (%) | 740 (99.2) | 370 (99.2) | 370 (99.2) | <0.001 | 1.00 |
| P2Y_12_ inhibitors, n (%) | 740 (99.2) | 369 (98.9) | 371 (99.5) | 0.060 | 0.41 |
| Statins, n (%) | 702 (94.1) | 351 (94.1) | 351 (94.1) | <0.001 | 1.00 |
| β blockers, n (%) | 597 (80.0) | 294 (78.8) | 303 (81.2) | 0.060 | 0.41 |

PSM = propensity score matching, ACEI = angiotensin-converting enzyme inhibitor, ARB = angiotensin receptor blocker, SMD = standardized mean difference, PCI = percutaneous coronary intervention, CABG = coronary artery bypass grafting, STEMI = ST-segment elevation myocardial infarction, SBP = systolic blood pressure, DBP = diastolic blood pressure, EF = ejection fraction, eGFR, estimated glomerular filtration rate, LDL-C = low-density lipoprotein cholesterol, hsCRP = high-sensitivity C-reactive protein, cTnI = cardiac troponin I, LM = left main, LAD = left anterior descending artery, LCX = left circumflex, RCA = right coronary artery, TIMI flow = Thrombolysis In Myocardial Infarction grade flow, D2B time = door-to-balloon time, CR = complete revascularization.

**Supplementary table 12.** Baseline characteristics of hypertensive patients in the PSM dataset stratified by ACEI/ARB medication.

| Variables | All patients (N=464) | No ACEI/ARB (N=232) | ACEI/ARB (N=232) | SMD | P value |
| --- | --- | --- | --- | --- | --- |
| Age, years | 60.1 ± 11.0 | 60.4 ± 11.0 | 59.9 ± 11.0 | 0.048 | 0.60 |
| Male sex, n (%) | 337 (72.6) | 171 (73.7) | 166 (71.6) | 0.048 | 0.60 |
| Hypertension, n (%) | 464 (100) | 232 (100) | 232 (100) | <0.001 | - |
| Diabetes, n (%) | 159 (34.3) | 79 (34.1) | 80 (34.5) | 0.009 | 0.92 |
| History of PCI or CABG, n (%) | 49 (10.6) | 33 (14.2) | 16 (6.9) | 0.240 | 0.010 |
| Peripheral artery diseases, n (%) | 26 (5.6) | 13 (5.6) | 13 (5.6) | <0.001 | 1.00 |
| STEMI, n (%) | 408 (87.9) | 196 (84.5) | 212 (91.4) | 0.213 | 0.023 |
| Hemodynamics |  |  |  |  |  |
| Heart rate, bpm | 76.1 ± 13.9 | 75.9 ± 14.4 | 76.3 ± 13.5 | 0.026 | 0.78 |
| SBP, mmHg | 122.6 ± 17.9 | 122.5 ± 17.9 | 122.7 ± 17.7 | 0.008 | 0.93 |
| DBP, mmHg | 72.7 ± 12.1 | 73.5 ± 12.2 | 71.9 ± 12.0 | 0.133 | 0.15 |
| EF, % | 57.3 ± 4.3 | 57.5 ± 4.2 | 57.1 ± 4.5 | 0.089 | 0.34 |
| Cardiac arrest, n (%) | 17 (3.7) | 8 (3.4) | 9 (3.9) | 0.023 | 0.80 |
| Lab tests |  |  |  |  |  |
| Creatinine, μmoI/L | 82.3 ± 25.9 | 81.6 ± 24.8 | 83.1 ± 26.9 | 0.055 | 0.55 |
| eGFR, ml/min | 82.1 (67.7-96.6) | 84.2 (70.4-97.3) | 80.9 (65.4-96.4) | 0.184 | 0.14 |
| LDL-C, mmol/L | 2.6 ± 0.9 | 2.6 ± 0.9 | 2.6 ± 0.8 | 0.050 | 0.59 |
| hsCRP, mg/L | 8.94 (3.49-12.25) | 8.01 (2.98-12.32) | 9.45 (4.17-12.24) | 0.161 | 0.22 |
| D-dimer, ng/mL | 331 (220-540) | 340 (230-555) | 320 (200-530) | 0.106 | 0.14 |
| Peak cTnI, ng/mL | 2.54 (1.00-6.76) | 2.62 (0.78-8.87) | 2.51 (1.12-5.86) | 0.025 | 0.74 |
| Coronary angiography findings |  |  |  |  |  |
| Culprit lesion, n (%) |  |  |  |  |  |
| LM | 5 (1.1) | 3 (1.3) | 2 (0.9) | 0.122 | 0.79 |
| LAD | 102 (22.0) | 51 (22.0) | 51 (22.0) |  |  |
| LCX | 96 (20.7) | 53 (22.8) | 43 (18.5) |  |  |
| RCA | 259 (55.8) | 124 (53.4) | 135 (58.2) |  |  |
| Bypass grafts | 2 (0.4) | 1 (0.4) | 1 (0.4) |  |  |
| Multivessel diseases, n (%) | 367 (79.1) | 186 (80.2) | 181 (78.0) | 0.053 | 0.57 |
| Pre-PCI TIMI 0 flow, n (%) | 320 (69.0) | 153 (65.9) | 167 (72.0) | 0.131 | 0.16 |
| Post-PCI TIMI 3 flow, n (%) | 449 (96.8) | 225 (97.0) | 224 (96.6) | 0.024 | 0.79 |
| D2B time ≥ 120 mins, n (%) | 250 (53.9) | 127 (54.7) | 123 (53.0) | 0.035 | 0.71 |
| CR before discharge, n (%) | 205 (44.2) | 97 (41.8) | 108 (46.6) | 0.096 | 0.30 |
| Medications at discharge |  |  |  |  |  |
| Aspirin, n (%) | 458 (98.7) | 229 (98.7) | 229 (98.7) | <0.001 | 1.00 |
| P2Y_12_ inhibitors, n (%) | 460 (99.1) | 230 (99.1) | 230 (99.1) | <0.001 | 1.00 |
| Statins, n (%) | 445 (95.9) | 223 (96.1) | 222 (95.7) | 0.022 | 0.81 |
| β blockers, n (%) | 397 (85.6) | 195 (84.1) | 202 (87.1) | 0.086 | 0.36 |

PSM = propensity score matching, ACEI = angiotensin-converting enzyme inhibitor, ARB = angiotensin receptor blocker, SMD = standardized mean difference, PCI = percutaneous coronary intervention, CABG = coronary artery bypass grafting, STEMI = ST-segment elevation myocardial infarction, SBP = systolic blood pressure, DBP = diastolic blood pressure, EF = ejection fraction, eGFR, estimated glomerular filtration rate, LDL-C = low-density lipoprotein cholesterol, hsCRP = high-sensitivity C-reactive protein, cTnI = cardiac troponin I, LM = left main, LAD = left anterior descending artery, LCX = left circumflex, RCA = right coronary artery, TIMI flow = Thrombolysis In Myocardial Infarction grade flow, D2B time = door-to-balloon time, CR = complete revascularization.

**Supplementary table 13.** Baseline characteristics of non-hypertensive patients in PSM dataset stratified by ACEI/ARB medication.

| Variables | All patients (N=610) | No ACEI/ARB (N=305) | ACEI/ARB (N=305) | SMD | P value |
| --- | --- | --- | --- | --- | --- |
| Age, years | 56.5 ± 11.5 | 56.5 ± 11.4 | 56.6 ± 11.7 | 0.002 | 0.98 |
| Male sex, n (%) | 518 (84.9) | 261 (85.6) | 257 (84.3) | 0.037 | 0.65 |
| Hypertension, n (%) | 0 (0) | 0 (0) | 0 (0) | <0.001 | - |
| Diabetes, n (%) | 174 (28.5) | 88 (28.9) | 86 (28.2) | 0.015 | 0.86 |
| History of PCI or CABG, n (%) | 52 (8.5) | 27 (8.9) | 25 (8.2) | 0.023 | 0.77 |
| Peripheral artery diseases, n (%) | 14 (2.3) | 7 (2.3) | 7 (2.3) | <0.001 | 1.00 |
| STEMI, n (%) | 531 (87.0) | 263 (86.2) | 268 (87.9) | 0.049 | 0.55 |
| Hemodynamics |  |  |  |  |  |
| Heart rate, bpm | 75.4 ± 13.7 | 75.6 ± 13.5 | 75.2 ± 13.9 | 0.030 | 0.71 |
| SBP, mmHg | 118.2 ± 15.0 | 118.2 ± 15.2 | 118.2 ± 14.7 | 0.004 | 0.97 |
| DBP, mmHg | 70.1 ± 10.8 | 71.1 ± 10.7 | 69.2 ± 10.8 | 0.173 | 0.033 |
| EF, % | 57.5 ± 4.0 | 57.5 ± 4.0 | 57.4 ± 4.1 | 0.033 | 0.68 |
| Cardiac arrest, n (%) | 16 (2.6) | 8 (2.6) | 8 (2.6) | <0.001 | 1.00 |
| Lab tests |  |  |  |  |  |
| Creatinine, μmoI/L | 78.3 ± 19.7 | 78.1 ± 21.4 | 78.6 ± 17.8 | 0.025 | 0.76 |
| eGFR, ml/min | 88.1 (76.2-102.2) | 88.9 (78.0-102.3) | 86.8 (75.2-102.0) | 0.077 | 0.21 |
| LDL-C, mmol/L | 2.8 ± 0.9 | 2.8 ± 0.9 | 2.8 ± 0.9 | 0.042 | 0.61 |
| hsCRP, mg/L | 5.26 (2.35-11.35) | 5.13 (2.14-11.23) | 5.28 (2.56-11.48) | 0.068 | 0.46 |
| D-dimer, ng/mL | 290 (210-419) | 290 (210-410) | 290 (210-420) | 0.001 | 0.91 |
| Peak cTnI, ng/mL | 2.20 (0.67-6.29) | 2.40 (0.67-7.78) | 1.96 (0.67-5.16) | 0.114 | 0.097 |
| Coronary angiography findings |  |  |  |  |  |
| Culprit lesion, n (%) |  |  |  |  |  |
| LM | 8 (1.3) | 5 (1.6) | 3 (1.0) | 0.063 | 0.90 |
| LAD | 170 (27.9) | 86 (28.2) | 84 (27.5) |  |  |
| LCX | 119 (19.5) | 58 (19.0) | 61 (20.0) |  |  |
| RCA | 313 (51.3) | 156 (51.1) | 157 (51.5) |  |  |
| Bypass grafts | 0 (0) | 0 (0) | 0 (0) |  |  |
| Multivessel diseases, n (%) | 436 (71.5) | 216 (70.8) | 220 (72.1) | 0.029 | 0.72 |
| Pre-PCI TIMI 0 flow, n (%) | 408 (66.9) | 197 (64.6) | 211 (69.2) | 0.098 | 0.23 |
| Post-PCI TIMI 3 flow, n (%) | 597 (97.9) | 297 (97.4) | 300 (98.4) | 0.068 | 0.40 |
| D2B time ≥ 120 mins, n (%) | 341 (55.9) | 166 (54.4) | 175 (57.4) | 0.059 | 0.46 |
| CR before discharge, n (%) | 279 (45.7) | 139 (45.6) | 140 (45.9) | 0.007 | 0.94 |
| Medications at discharge |  |  |  |  |  |
| Aspirin, n (%) | 608 (99.7) | 304 (99.7) | 304 (99.7) | <0.001 | 1.00 |
| P2Y_12_ inhibitors, n (%) | 606 (99.3) | 303 (99.3) | 303 (99.3) | <0.001 | 1.00 |
| Statins, n (%) | 570 (93.4) | 286 (93.8) | 284 (93.1) | 0.026 | 0.74 |
| β blockers, n (%) | 496 (81.3) | 247 (81.0) | 249 (81.6) | 0.017 | 0.84 |

PSM = propensity score matching, ACEI = angiotensin-converting enzyme inhibitor, ARB = angiotensin receptor blocker, SMD = standardized mean difference, PCI = percutaneous coronary intervention, CABG = coronary artery bypass grafting, STEMI = ST-segment elevation myocardial infarction, SBP = systolic blood pressure, DBP = diastolic blood pressure, EF = ejection fraction, eGFR, estimated glomerular filtration rate, LDL-C = low-density lipoprotein cholesterol, hsCRP = high-sensitivity C-reactive protein, cTnI = cardiac troponin I, LM = left main, LAD = left anterior descending artery, LCX = left circumflex, RCA = right coronary artery, TIMI flow = Thrombolysis In Myocardial Infarction grade flow, D2B time = door-to-balloon time, CR = complete revascularization.

**Supplementary table 14.** Baseline characteristics of anterior infarction patients in PSM dataset stratified by ACEI/ARB medication.

| Variables | All patients (N=282) | No ACEI/ARB (N=141) | ACEI/ARB (N=141) | SMD | P value |
| --- | --- | --- | --- | --- | --- |
| Age, years | 56.5 ± 11.4 | 56.8 ± 11.2 | 56.3 ± 11.8 | 0.043 | 0.72 |
| Male sex, n (%) | 221 (78.4) | 111 (78.7) | 110 (78.0) | 0.017 | 0.89 |
| Hypertension, n (%) | 112 (39.7) | 54 (38.3) | 58 (41.1) | 0.058 | 0.63 |
| Diabetes, n (%) | 91 (32.3) | 48 (34.0) | 43 (30.5) | 0.076 | 0.52 |
| History of PCI or CABG, n (%) | 26 (9.2) | 14 (9.9) | 12 (8.5) | 0.049 | 0.68 |
| Peripheral artery diseases, n (%) | 11 (3.9) | 7 (5.0) | 4 (2.8) | 0.110 | 0.36 |
| STEMI, n (%) | 235 (83.3) | 114 (80.9) | 121 (85.8) | 0.134 | 0.26 |
| Hemodynamics |  |  |  |  |  |
| Heart rate, bpm | 74.5 ± 11.9 | 74.0 ± 11.2 | 75.1 ± 12.6 | 0.094 | 0.43 |
| SBP, mmHg | 122.7 ± 15.9 | 122.4 ± 16.1 | 123.0 ± 15.6 | 0.035 | 0.77 |
| DBP, mmHg | 74.2 ± 12.1 | 74.6 ± 12.4 | 73.7 ± 11.8 | 0.074 | 0.54 |
| EF, % | 56.7 ± 4.6 | 56.8 ± 4.5 | 56.7 ± 4.7 | 0.026 | 0.83 |
| Cardiac arrest, n (%) | 9 (3.2) | 4 (2.8) | 5 (3.5) | 0.040 | 0.73 |
| Lab tests |  |  |  |  |  |
| Creatinine, μmoI/L | 78.3 ± 23.0 | 78.2 ± 24.8 | 78.3 ± 21.2 | 0.002 | 0.99 |
| eGFR, ml/min | 86.0 (73.9-102.6) | 87.7 (76.3-103.9) | 84.5 (70.1-101.2) | 0.204 | 0.20 |
| LDL-C, mmol/L | 2.6 ± 0.8 | 2.6 ± 0.8 | 2.6 ± 0.8 | 0.002 | 0.98 |
| hsCRP, mg/L | 5.72 (2.21-11.62) | 4.89 (1.99-11.69) | 6.61 (2.28-11.50) | 0.116 | 0.48 |
| D-dimer, ng/mL | 270 (196-394) | 270 (200-410) | 270 (170-388) | 0.049 | 0.57 |
| Peak cTnI, ng/mL | 2.19 (0.75-5.57) | 2.45 (0.67-7.43) | 1.98 (0.88-4.63) | 0.055 | 0.46 |
| Coronary angiography findings |  |  |  |  |  |
| Culprit lesion, n (%) |  |  |  |  |  |
| LM | 14 (5.0) | 8 (5.7) | 6 (4.3) | 0.065 | 0.58 |
| LAD | 268 (95.0) | 133 (94.3) | 135 (95.7) |  |  |
| LCX | 0 (0) | 0 (0) | 0 (0) |  |  |
| RCA | 0 (0) | 0 (0) | 0 (0) |  |  |
| Bypass grafts | 0 (0) | 0 (0) | 0 (0) |  |  |
| Multivessel diseases, n (%) | 158 (56.0) | 85 (60.3) | 73 (51.8) | 0.172 | 0.15 |
| Pre-PCI TIMI 0 flow, n (%) | 169 (59.9) | 76 (53.9) | 93 (66.0) | 0.248 | 0.039 |
| Post-PCI TIMI 3 flow, n (%) | 272 (96.5) | 136 (96.5) | 136 (96.5) | <0.001 | 1.00 |
| D2B time ≥ 120 mins, n (%) | 168 (59.6) | 86 (61.0) | 82 (58.2) | 0.058 | 0.63 |
| CR before discharge, n (%) | 163 (57.8) | 79 (56.0) | 84 (59.6) | 0.072 | 0.55 |
| Medications at discharge |  |  |  |  |  |
| Aspirin, n (%) | 280 (99.3) | 140 (99.3) | 140 (99.3) | <0.001 | 1.00 |
| P2Y_12_ inhibitors, n (%) | 281 (99.6) | 141 (100.0) | 140 (99.3) | 0.120 | 0.32 |
| Statins, n (%) | 266 (94.3) | 133 (94.3) | 133 (94.3) | <0.001 | 1.00 |
| β blockers, n (%) | 257 (91.1) | 129 (91.5) | 128 (90.8) | 0.025 | 0.83 |

PSM = propensity score matching, ACEI = angiotensin-converting enzyme inhibitor, ARB = angiotensin receptor blocker, SMD = standardized mean difference, PCI = percutaneous coronary intervention, CABG = coronary artery bypass grafting, STEMI = ST-segment elevation myocardial infarction, SBP = systolic blood pressure, DBP = diastolic blood pressure, EF = ejection fraction, eGFR, estimated glomerular filtration rate, LDL-C = low-density lipoprotein cholesterol, hsCRP = high-sensitivity C-reactive protein, cTnI = cardiac troponin I, LM = left main, LAD = left anterior descending artery, LCX = left circumflex, RCA = right coronary artery, TIMI flow = Thrombolysis In Myocardial Infarction grade flow, D2B time = door-to-balloon time, CR = complete revascularization.

**Supplementary table 15.** Baseline characteristics of non-anterior infarction patients in PSM dataset by ACEI/ARB medication.

| Variables | All patients (N=792) | No ACEI/ARB (N=396) | ACEI/ARB (N=396) | SMD | P value |
| --- | --- | --- | --- | --- | --- |
| Age, years | 58.8 ± 11.3 | 58.7 ± 11.4 | 58.8 ± 11.3 | 0.003 | 0.96 |
| Male sex, n (%) | 638 (80.6) | 319 (80.6) | 319 (80.6) | <0.001 | 1.00 |
| Hypertension, n (%) | 356 (44.9) | 180 (45.5) | 176 (44.4) | 0.020 | 0.78 |
| Diabetes, n (%) | 242 (30.6) | 119 (30.1) | 123 (31.1) | 0.022 | 0.76 |
| History of PCI or CABG, n (%) | 79 (10.0) | 44 (11.1) | 35 (8.8) | 0.076 | 0.29 |
| Peripheral artery diseases, n (%) | 25 (3.2) | 11 (2.8) | 14 (3.5) | 0.043 | 0.54 |
| STEMI, n (%) | 703 (88.8) | 345 (87.1) | 358 (90.4) | 0.104 | 0.14 |
| Hemodynamics |  |  |  |  |  |
| Heart rate, bpm | 76.2 ± 14.7 | 76.0 ± 15.2 | 76.3 ± 14.2 | 0.016 | 0.82 |
| SBP, mmHg | 119.2 ± 16.0 | 119.5 ± 16.2 | 118.8 ± 15.8 | 0.046 | 0.52 |
| DBP, mmHg | 70.1 ± 10.9 | 71.0 ± 10.7 | 69.1 ± 11.1 | 0.179 | 0.012 |
| EF, % | 57.6 ± 4.0 | 57.8 ± 3.9 | 57.5 ± 4.1 | 0.078 | 0.27 |
| Cardiac arrest, n (%) | 21 (2.7) | 12 (3.0) | 9 (2.3) | 0.047 | 0.51 |
| Lab tests |  |  |  |  |  |
| Creatinine, μmoI/L | 81.8 ± 22.7 | 80.6 ± 22.6 | 83.1 ± 22.7 | 0.111 | 0.12 |
| eGFR, ml/min | 85.2 (70.2-98.3) | 86.1 (73.0-100.4) | 84.2 (68.0-97.2) | 0.083 | 0.020 |
| LDL-C, mmol/L | 2.7 ± 0.9 | 2.7 ± 0.9 | 2.8 ± 0.9 | 0.074 | 0.30 |
| hsCRP, mg/L | 6.74 (2.86-11.74) | 6.83 (2.69-11.71) | 6.67 (3.02-11.79) | 0.052 | 0.69 |
| D-dimer, ng/mL | 333 (220-502) | 320 (220-490) | 336 (220-519) | 0.021 | 0.83 |
| Peak cTnI, ng/mL | 2.37 (0.81-7.15) | 2.52 (0.76-8.78) | 2.24 (0.89-5.86) | 0.023 | 0.31 |
| Coronary angiography findings |  |  |  |  |  |
| Culprit lesion, n (%) |  |  |  |  |  |
| LM | 0 (0) | 0 (0) | 0 (0) | 0.040 | 0.85 |
| LAD | 0 (0) | 0 (0) | 0 (0) |  |  |
| LCX | 213 (26.9) | 110 (27.8) | 103 (26.0) |  |  |
| RCA | 577 (72.9) | 285 (72.0) | 292 (73.7) |  |  |
| Bypass grafts | 2 (0.3) | 1 (0.3) | 1 (0.3) |  |  |
| Multivessel diseases, n (%) | 649 (81.9) | 318 (80.3) | 331 (83.6) | 0.085 | 0.23 |
| Pre-PCI TIMI 0 flow, n (%) | 565 (71.3) | 272 (68.7) | 293 (74.0) | 0.117 | 0.099 |
| Post-PCI TIMI 3 flow, n (%) | 776 (98.0) | 388 (98.0) | 388 (98.0) | <0.001 | 1.00 |
| D2B time ≥ 120 mins, n (%) | 425 (53.7) | 207 (52.3) | 218 (55.1) | 0.056 | 0.43 |
| CR before discharge, n (%) | 315 (39.8) | 157 (39.6) | 158 (39.9) | 0.071 | 0.94 |
| Medications at discharge |  |  |  |  |  |
| Aspirin, n (%) | 788 (99.5) | 393 (99.2) | 395 (99.7) | 0.071 | 0.32 |
| P2Y_12_ inhibitors, n (%) | 787 (99.4) | 392 (99.0) | 395 (99.7) | 0.096 | 0.18 |
| Statins, n (%) | 744 (93.9) | 373 (94.2) | 371 (93.7) | 0.021 | 0.77 |
| β blockers, n (%) | 623 (78.7) | 309 (78.0) | 314 (79.3) | 0.031 | 0.66 |

PSM = propensity score matching, ACEI = angiotensin-converting enzyme inhibitor, ARB = angiotensin receptor blocker, SMD = standardized mean difference, PCI = percutaneous coronary intervention, CABG = coronary artery bypass grafting, STEMI = ST-segment elevation myocardial infarction, SBP = systolic blood pressure, DBP = diastolic blood pressure, EF = ejection fraction, eGFR, estimated glomerular filtration rate, LDL-C = low-density lipoprotein cholesterol, hsCRP = high-sensitivity C-reactive protein, cTnI = cardiac troponin I, LM = left main, LAD = left anterior descending artery, LCX = left circumflex, RCA = right coronary artery, TIMI flow = Thrombolysis In Myocardial Infarction grade flow, D2B time = door-to-balloon time, CR = complete revascularization.

**Supplementary table 16.** Baseline characteristics of patients in the PSM dataset by the medication of ACEI.

| Variables | All patients (N=1074) | No ACEI (N=537) | ACEI (N=537) | SMD | P value |
| --- | --- | --- | --- | --- | --- |
| Age, years | 58.0 ± 11.2 | 58.0 ± 11.2 | 57.9 ± 11.2 | 0.008 | 0.90 |
| Male sex, n (%) | 857 (79.8) | 430 (80.1) | 427 (79.5) | 0.014 | 0.82 |
| Hypertension, n (%) | 474 (44.1) | 232 (43.2) | 242 (45.1) | 0.038 | 0.54 |
| Diabetes, n (%) | 326 (30.4) | 165 (30.7) | 161 (30.0) | 0.016 | 0.79 |
| History of PCI or CABG, n (%) | 101 (9.4) | 59 (11.0) | 42 (7.8) | 0.109 | 0.076 |
| Peripheral artery diseases, n (%) | 44 (4.1) | 21 (3.9) | 23 (4.3) | 0.019 | 0.76 |
| STEMI, n (%) | 942 (87.7) | 459 (85.5) | 483 (89.9) | 0.136 | 0.026 |
| Hemodynamics |  |  |  |  |  |
| Heart rate, bpm | 75.7 ± 13.8 | 75.7 ± 14.0 | 75.7 ± 13.7 | 0.003 | 0.96 |
| SBP, mmHg | 119.9 ± 16.3 | 119.9 ± 16.6 | 119.8 ± 16.1 | 0.006 | 0.92 |
| DBP, mmHg | 71.1 ± 11.4 | 71.9 ± 11.4 | 70.4 ± 11.5 | 0.139 | 0.023 |
| EF, % | 57.4 ± 4.2 | 57.6 ± 4.1 | 57.2 ± 4.2 | 0.084 | 0.17 |
| Cardiac arrest, n (%) | 33 (3.1) | 17 (3.2) | 16 (3.0) | 0.011 | 0.86 |
| Lab tests |  |  |  |  |  |
| Creatinine, μmoI/L | 80.3 ± 22.4 | 80.2 ± 23.2 | 80.4 ± 21.6 | 0.009 | 0.88 |
| eGFR, ml/min | 85.8 (72.1-99.4) | 86.2 (74.4-100.5) | 85.1 (70.0-98.6) | 0.147 | 0.14 |
| LDL-C, mmol/L | 2.7 ± 0.9 | 2.7 ± 0.9 | 2.7 ± 0.9 | 0.034 | 0.57 |
| hsCRP, mg/L | 6.62 (2.73-11.84) | 6.30 (2.43-11.71) | 7.00 (3.05-11.92) | 0.137 | 0.11 |
| D-dimer, ng/mL | 310 (220-470) | 310 (220-480) | 300 (220-450) | 0.034 | 0.43 |
| Peak cTnI, ng/mL | 2.30 (0.79-6.26) | 2.41 (0.68-8.06) | 2.17 (0.90-5.08) | 0.033 | 0.24 |
| Coronary angiography findings |  |  |  |  |  |
| Culprit lesion, n (%) |  |  |  |  |  |
| LM | 15 (1.4) | 9 (1.7) | 6 (1.1) | 0.067 | 0.88 |
| LAD | 270 (25.1) | 140 (26.1) | 130 (24.2) |  |  |
| LCX | 212 (19.7) | 105 (19.6) | 107 (19.9) |  |  |
| RCA | 575 (53.5) | 282 (52.5) | 293 (54.6) |  |  |
| Bypass grafts | 2 (0.2) | 1 (0.2) | 1 (0.2) |  |  |
| Multivessel diseases, n (%) | 803 (74.8) | 399 (74.3) | 404 (75.2) | 0.021 | 0.73 |
| Pre-PCI TIMI 0 flow, n (%) | 735 (68.4) | 352 (65.5) | 383 (71.3) | 0.124 | 0.042 |
| Post-PCI TIMI 3 flow, n (%) | 1046 (97.4) | 523 (97.4) | 523 (97.4) | <0.001 | 1.00 |
| D2B time ≥ 120 mins, n (%) | 587 (54.7) | 294 (54.7) | 293 (54.6) | 0.004 | 0.95 |
| CR before discharge, n (%) | 493 (45.9) | 241 (44.9) | 252 (46.9) | 0.041 | 0.50 |
| Medications at discharge |  |  |  |  |  |
| Aspirin, n (%) | 1067 (99.3) | 533 (99.3) | 534 (99.4) | 0.023 | 0.70 |
| P2Y_12_ inhibitors, n (%) | 1073 (99.9) | 537 (100.0) | 536 (99.8) | 0.061 | 0.32 |
| Statins, n (%) | 1008 (93.9) | 506 (94.2) | 502 (93.5) | 0.031 | 0.61 |
| β blockers, n (%) | 874 (81.4) | 436 (81.2) | 438 (81.6) | 0.010 | 0.88 |

PSM = propensity score matching, ACEI = angiotensin-converting enzyme inhibitor, SMD = standardized mean difference, PCI = percutaneous coronary intervention, CABG = coronary artery bypass grafting, STEMI = ST-segment elevation myocardial infarction, SBP = systolic blood pressure, DBP = diastolic blood pressure, EF = ejection fraction, eGFR, estimated glomerular filtration rate, LDL-C = low-density lipoprotein cholesterol, hsCRP = high-sensitivity C-reactive protein, cTnI = cardiac troponin I, LM = left main, LAD = left anterior descending artery, LCX = left circumflex, RCA = right coronary artery, TIMI flow = Thrombolysis In Myocardial Infarction grade flow, D2B time = door-to-balloon time, CR = complete revascularization.

**Supplementary table 17.** Baseline characteristics of patients in the PSM dataset by the medication of ARB.

| Variables | All patients (N=322) | No ARB (N=161) | ARB (N=161) | SMD | P value |
| --- | --- | --- | --- | --- | --- |
| Age, years | 58.6 ± 12.1 | 58.6 ± 11.8 | 58.6 ± 12.4 | 0.001 | 1.00 |
| Male sex, n (%) | 246 (76.4) | 120 (74.5) | 126 (78.3) | 0.088 | 0.43 |
| Hypertension, n (%) | 252 (78.3) | 126 (78.3) | 126 (78.3) | <0.001 | 1.00 |
| Diabetes, n (%) | 118 (36.6) | 63 (39.1) | 55 (34.2) | 0.103 | 0.35 |
| History of PCI or CABG, n (%) | 52 (16.1) | 27 (16.8) | 25 (15.5) | 0.034 | 0.76 |
| Peripheral artery diseases, n (%) | 9 (2.8) | 5 (3.1) | 4 (2.5) | 0.038 | 0.74 |
| STEMI, n (%) | 274 (85.1) | 137 (85.1) | 137 (85.1) | <0.001 | 1.00 |
| Hemodynamics |  |  |  |  |  |
| Heart rate, bpm | 74.7 ± 13.3 | 74.9 ± 12.9 | 74.4 ± 13.8 | 0.036 | 0.75 |
| SBP, mmHg | 129.2 ± 18.6 | 129.0 ± 17.8 | 129.3 ± 19.4 | 0.017 | 0.88 |
| DBP, mmHg | 75.4 ± 12.9 | 75.6 ± 12.6 | 75.3 ± 13.2 | 0.023 | 0.84 |
| EF, % | 58.0 ± 4.1 | 58.0 ± 4.3 | 58.0 ± 4.0 | 0.009 | 0.93 |
| Cardiac arrest, n (%) | 8 (2.5) | 5 (3.1) | 3 (1.9) | 0.080 | 0.47 |
| Lab tests |  |  |  |  |  |
| Creatinine, μmoI/L | 84.0 ± 25.8 | 83.0 ± 29.0 | 85.0 ± 22.2 | 0.075 | 0.50 |
| eGFR, ml/min | 83.4 (67.4-96.8) | 85.3 (70.6-100.1) | 82.2 (64.9-93.2) | 0.183 | 0.14 |
| LDL-C, mmol/L | 2.7 ± 0.9 | 2.8 ± 1.0 | 2.7 ± 0.9 | 0.091 | 0.42 |
| hsCRP, mg/L | 6.07 (2.44-11.38) | 5.74 (2.32-11.29) | 6.20 (2.58-11.38) | 0.035 | 0.58 |
| D-dimer, ng/mL | 310 (200-510) | 310 (215-490) | 310 (200-510) | 0.039 | 0.94 |
| Peak cTnI, ng/mL | 1.88 (0.52-5.86) | 1.73 (0.54-5.68) | 2.04 (0.49-6.09) | 0.071 | 0.56 |
| Coronary angiography findings |  |  |  |  |  |
| Culprit lesion, n (%) |  |  |  |  |  |
| LM | 0 (0) | 0 (0) | 0 (0) | 0.050 | 0.91 |
| LAD | 88 (27.3) | 45 (28.0) | 43 (26.7) |  |  |
| LCX | 74 (23.0) | 38 (23.6) | 36 (22.4) |  |  |
| RCA | 160 (49.7) | 78 (48.4) | 82 (50.9) |  |  |
| Bypass grafts | 0 (0) | 0 (0) | 0 (0) |  |  |
| Multivessel diseases, n (%) | 247 (76.7) | 124 (77.0) | 123 (76.4) | 0.015 | 0.90 |
| Pre-PCI TIMI 0 flow, n (%) | 211 (65.5) | 110 (68.3) | 101 (62.7) | 0.118 | 0.29 |
| Post-PCI TIMI 3 flow, n (%) | 314 (97.5) | 156 (96.9) | 158 (98.1) | 0.080 | 0.47 |
| D2B time ≥ 120 mins, n (%) | 196 (60.9) | 94 (58.4) | 102 (63.4) | 0.102 | 0.36 |
| CR before discharge, n (%) | 154 (47.8) | 78 (48.4) | 76 (47.2) | 0.025 | 0.82 |
| Medications at discharge |  |  |  |  |  |
| Aspirin, n (%) | 320 (99.4) | 160 (99.4) | 160 (99.4) | <0.001 | 1.00 |
| P2Y_12_ inhibitors, n (%) | 320 (99.4) | 160 (99.4) | 160 (99.4) | <0.001 | 1.00 |
| Statins, n (%) | 309 (96.0) | 155 (96.3) | 154 (95.7) | 0.032 | 0.78 |
| β blockers, n (%) | 284 (88.2) | 141 (87.6) | 143 (88.8) | 0.039 | 0.73 |

PSM = propensity score matching, ARB = angiotensin receptor blocker, SMD = standardized mean difference, PCI = percutaneous coronary intervention, CABG = coronary artery bypass grafting, STEMI = ST-segment elevation myocardial infarction, SBP = systolic blood pressure, DBP = diastolic blood pressure, EF = ejection fraction, eGFR, estimated glomerular filtration rate, LDL-C = low-density lipoprotein cholesterol, hsCRP = high-sensitivity C-reactive protein, cTnI = cardiac troponin I, LM = left main, LAD = left anterior descending artery, LCX = left circumflex, RCA = right coronary artery, TIMI flow = Thrombolysis In Myocardial Infarction grade flow, D2B time = door-to-balloon time, CR = complete revascularization.

**Supplementary table 18.** Baseline characteristics of diabetic patients in the PSMW dataset stratified by ACEI/ARB medication.

| Variables | All patients (N=333.19) | No ACEI/ARB (N=166.89) | ACEI/ARB (N=166.30) | SMD |
| --- | --- | --- | --- | --- |
| Age, years | 60.0 ± 11.3 | 60.0 ± 11.6 | 59.9 ± 11.0 | 0.006 |
| Male sex, n (%) | 257.3 (77.2) | 128.4 (77.0) | 128.8 (77.5) | 0.012 |
| Hypertension, n (%) | 157.5 (47.3) | 78.6 (47.1) | 78.9 (47.5) | 0.008 |
| Diabetes, n (%) | 333.2 (100) | 166.9 (100) | 166.3 (100) | <0.001 |
| History of PCI or CABG, n (%) | 52.4 (15.7) | 26.2 (15.7) | 26.2 (15.8) | 0.002 |
| Peripheral artery diseases, n (%) | 9.7 (2.9) | 4.8 (2.9) | 4.9 (3.0) | 0.004 |
| STEMI, n (%) | 282.7 (84.9) | 141.1 (84.6) | 141.6 (85.2) | 0.017 |
| Hemodynamics |  |  |  |  |
| Heart rate, bpm | 76.0 ± 13.4 | 76.1 ± 14.0 | 76.0 ± 12.7 | 0.008 |
| SBP, mmHg | 120.2 ± 15.8 | 120.1 ± 16.3 | 120.2 ± 16.4 | 0.005 |
| DBP, mmHg | 71.4 ± 11.2 | 71.2 ± 11.4 | 71.5 ± 11.1 | 0.023 |
| EF, % | 57.7 ± 4.1 | 57.7 ± 4.0 | 57.7 ± 4.2 | 0.007 |
| Cardiac arrest, n (%) | 5.6 (1.7) | 2.9 (1.8) | 2.6 (1.6) | 0.013 |
| Lab tests |  |  |  |  |
| Creatinine, μmoI/L | 78.1 ± 23.4 | 78.1 ± 25.7 | 78.1 ± 21.0 | 0.001 |
| eGFR, ml/min | 87.6 (75.3-101.6) | 87.3 (76.2-102.2) | 88.1 (75.0-100.4) | 0.008 |
| LDL-C, mmol/L | 2.6 ± 0.8 | 2.6 ± 0.8 | 2.6 ± 0.8 | 0.001 |
| hsCRP, mg/L | 6.20 (2.56-11.65) | 6.35 (2.32-11.48) | 5.98 (3.00-11.78) | 0.016 |
| D-dimer, ng/mL | 290 (220-450) | 280 (210-440) | 300 (220-450) | 0.023 |
| Peak cTnI, ng/mL | 1.85 (0.49-6.21) | 1.85 (0.44-6.47) | 1.82 (0.49-5.21) | 0.008 |
| Coronary angiography findings |  |  |  |  |
| Culprit lesion, n (%) |  |  |  |  |
| LM | 1.9 (0.6) | 1.0 (0.6) | 0.9 (0.6) | 0.028 |
| LAD | 94.4 (28.3) | 48.2 (28.9) | 46.2 (27.8) |  |
| LCX | 63.8 (19.2) | 31.3 (18.8) | 32.5 (19.5) |  |
| RCA | 173.1 (51.9) | 86.4 (51.8) | 86.7 (52.1) |  |
| Bypass grafts | 0 (0) | 0 (0) | 0 (0) |  |
| Multivessel diseases, n (%) | 263.7 (79.2) | 131.6 (78.9) | 132.1 (79.5) | 0.015 |
| Pre-PCI TIMI 0 flow, n (%) | 210.9 (63.3) | 104.7 (62.7) | 106.2 (63.8) | 0.023 |
| Post-PCI TIMI 3 flow, n (%) | 323.2 (97.0) | 161.9 (97.0) | 161.4 (97.0) | 0.001 |
| D2B time ≥ 120 mins, n (%) | 195.2 (58.6) | 99.3 (59.5) | 95.8 (57.6) | 0.004 |
| CR before discharge, n (%) | 142.8 (42.9) | 71.4 (42.8) | 71.4 (43.0) | 0.004 |
| Medications at discharge |  |  |  |  |
| Aspirin, n (%) | 330.1 (99.1) | 165.3 (99.1) | 164.8 (99.1) | <0.001 |
| P2Y_12_ inhibitors, n (%) | 331.0 (99.3) | 165.7 (99.3) | 165.3 (99.4) | 0.015 |
| Statins, n (%) | 314.8 (94.5) | 158.0 (94.7) | 156.8 (94.3) | 0.019 |
| β blockers, n (%) | 288.3 (86.5) | 144.4 (86.5) | 143.9 (86.5) | <0.001 |

PSMW = propensity score matching weight, ACEI = angiotensin-converting enzyme inhibitor, ARB = angiotensin receptor blocker, SMD = standardized mean difference, PCI = percutaneous coronary intervention, CABG = coronary artery bypass grafting, STEMI = ST-segment elevation myocardial infarction, SBP = systolic blood pressure, DBP = diastolic blood pressure, EF = ejection fraction, eGFR = estimated glomerular filtration rate, LDL-C = low-density lipoprotein cholesterol, hsCRP = high-sensitivity C-reactive protein, cTnI = cardiac troponin I, LM = left main, LAD = left anterior descending artery, LCX = left circumflex, RCA = right coronary artery, TIMI flow = Thrombolysis In Myocardial Infarction grade flow, D2B time = door-to-balloon time, CR = complete revascularization.

**Supplementary table 19.** Baseline characteristics of non-diabetic patients in the PSMW dataset stratified by ACEI/ARB medication.

| Variables | All patients (N=754.24) | No ACEI/ARB (N=379.21) | ACEI/ARB (N=375.03) | SMD |
| --- | --- | --- | --- | --- |
| Age, years | 57.3 ± 11.7 | 57.3 ± 11.3 | 57.3 ± 12.2 | 0.003 |
| Male sex, n (%) | 613.1 (81.3) | 309.6 (81.6) | 303.6 (80.9) | 0.018 |
| Hypertension, n (%) | 309.6 (41.0) | 154.6 (40.8) | 155.0 (41.3) | 0.011 |
| Diabetes, n (%) | 754.2 (100.0) | 379.2 (100.0) | 375.0 (100.0) | <0.001 |
| History of PCI or CABG, n (%) | 67.2 (8.9) | 33.8 (8.9) | 33.4 (8.9) | <0.001 |
| Peripheral artery diseases, n (%) | 31.0 (4.1) | 14.9 (3.9) | 16.2 (4.3) | 0.019 |
| STEMI, n (%) | 648.3 (86.0) | 324.8 (85.7) | 323.5 (86.3) | 0.017 |
| Hemodynamics |  |  |  |  |
| Heart rate, bpm | 75.5 ± 14.3 | 75.5 ± 14.1 | 75.4 ± 14.5 | 0.006 |
| SBP, mmHg | 120.1 ± 16.4 | 120.0 ± 16.8 | 120.1 ± 16.0 | 0.007 |
| DBP, mmHg | 72.3 ± 11.7 | 72.2 ± 11.4 | 72.3 ± 12.0 | 0.011 |
| EF, % | 57.4 ± 4.1 | 57.4 ± 4.1 | 57.4 ± 4.0 | 0.008 |
| Cardiac arrest, n (%) | 26.7 (3.5) | 13.2 (3.5) | 13.5 (3.6) | 0.005 |
| Lab tests |  |  |  |  |
| Creatinine, μmoI/L | 80.8 ± 21.2 | 80.9 ± 23.4 | 80.7 ± 18.8 | 0.013 |
| eGFR, ml/min | 85.7 (72.2-99.0) | 85.6 (73.3-99.4) | 85.7 (71.6-98.2) | 0.024 |
| LDL-C, mmol/L | 2.8 ± 0.9 | 2.8 ± 0.9 | 2.8 ± 0.9 | 0.001 |
| hsCRP, mg/L | 5.86 (2.50-11.67) | 6.23 (2.49-11.75) | 5.74 (2.56-11.58) | 0.001 |
| D-dimer, ng/mL | 312 (220-490) | 320 (220-490) | 310 (220-497) | 0.005 |
| Peak cTnI, ng/mL | 2.82 (0.84-8.98) | 2.67 (0.93-8.85) | 2.89 (0.79-9.12) | 0.015 |
| Coronary angiography findings |  |  |  |  |
| Culprit lesion, n (%) |  |  |  |  |
| LM | 14.1 (1.9) | 7.2 (1.9) | 7.0 (1.9) | 0.013 |
| LAD | 181.0 (24.0) | 90.9 (24.0) | 90.1 (24.0) |  |
| LCX | 155.0 (20.6) | 77.3 (20.4) | 77.7 (20.7) |  |
| RCA | 402.0 (53.3) | 202.9 (53.5) | 199.1 (53.1) |  |
| Bypass grafts | 2.1 (0.3) | 1.0 (0.3) | 1.1 (0.3) |  |
| Multivessel diseases, n (%) | 557.9 (74.0) | 279.5 (73.7) | 278.4 (74.2) | 0.012 |
| Pre-PCI TIMI 0 flow, n (%) | 504.4 (66.9) | 253.1 (66.7) | 251.3 (67.0) | 0.005 |
| Post-PCI TIMI 3 flow, n (%) | 736.4 (97.6) | 370.1 (97.6) | 366.3 (97.7) | 0.004 |
| D2B time ≥ 120 mins, n (%) | 395.0 (52.4) | 198.5 (52.4) | 196.5 (52.4) | 0.001 |
| CR before discharge, n (%) | 334.6 (44.4) | 168.5 (44.4) | 166.1 (44.3) | 0.003 |
| Medications at discharge |  |  |  |  |
| Aspirin, n (%) | 749.6 (99.4) | 376.7 (99.3) | 372.9 (99.4) | 0.012 |
| P2Y_12_ inhibitors, n (%) | 748.7 (99.3) | 376.0 (99.2) | 372.7 (99.4) | 0.024 |
| Statins, n (%) | 710.6 (94.2) | 356.7 (94.1) | 353.9 (94.4) | 0.013 |
| β blockers, n (%) | 595.0 (78.9) | 297.8 (78.5) | 297.2 (79.2) | 0.017 |

PSMW = propensity score matching weight, ACEI = angiotensin-converting enzyme inhibitor, ARB = angiotensin receptor blocker, SMD = standardized mean difference, PCI = percutaneous coronary intervention, CABG = coronary artery bypass grafting, STEMI = ST-segment elevation myocardial infarction, SBP = systolic blood pressure, DBP = diastolic blood pressure, eGFR, estimated glomerular filtration rate, EF = ejection fraction, LDL-C = low-density lipoprotein cholesterol, hsCRP = high-sensitivity C-reactive protein, cTnI = cardiac troponin I, LM = left main, LAD = left anterior descending artery, LCX = left circumflex, RCA = right coronary artery, TIMI flow = Thrombolysis In Myocardial Infarction grade flow, D2B time = door-to-balloon time, CR = complete revascularization.

**Supplementary table 20.** Baseline characteristics of hypertensive patients in the PSMW dataset stratified by ACEI/ARB medication.

| Variables | All patients (N=465.75) | No ACEI/ARB (N=233.29) | ACEI/ARB (N=232.46) | SMD |
| --- | --- | --- | --- | --- |
| Age, years | 60.5 ± 11.0 | 60.5 ± 11.0 | 60.5 ± 11.0 | 0.004 |
| Male sex, n (%) | 342.1 (73.4) | 171.9 (73.7) | 170.2 (73.2) | 0.011 |
| Hypertension, n (%) | 465.7 (100) | 233.3 (100) | 232.5 (100) | <0.001 |
| Diabetes, n (%) | 158.7 (34.1) | 79.2 (33.9) | 79.6 (34.2) | 0.006 |
| History of PCI or CABG, n (%) | 64.9 (13.9) | 32.9 (14.1) | 32.0 (13.8) | 0.011 |
| Peripheral artery diseases, n (%) | 28.8 (6.2) | 13.6 (5.8) | 15.2 (6.6) | 0.031 |
| STEMI, n (%) | 393.6 (84.5) | 197.4 (84.6) | 196.3 (84.4) | 0.005 |
| Hemodynamics |  |  |  |  |
| Heart rate, bpm | 76.1 ± 14.3 | 76.1 ± 14.7 | 76.0 ± 14.0 | 0.004 |
| SBP, mmHg | 122.5 ± 17.4 | 122.5 ± 17.9 | 122.6 ± 16.9 | 0.009 |
| DBP, mmHg | 73.5 ± 12.1 | 73.5 ± 12.2 | 73.6 ± 11.9 | 0.013 |
| EF, % | 57.5 ± 4.1 | 57.5 ± 4.2 | 57.6 ± 4.0 | 0.017 |
| Cardiac arrest, n (%) | 18.1 (3.9) | 8.7 (3.7) | 9.4 (4.0) | 0.016 |
| Lab tests |  |  |  |  |
| Creatinine, μmoI/L | 81.7 ± 24.1 | 81.6 ± 24.9 | 81.8 ± 23.2 | 0.007 |
| eGFR, ml/min | 83.9 (69.7-86.7) | 83.9 (69.9-97.2) | 83.6 (67.9-96.5) | 0.058 |
| LDL-C, mmol/L | 2.6 ± 0.9 | 2.6 ± 0.9 | 2.6 ± 0.8 | 0.007 |
| hsCRP, mg/L | 7.60 (3.09-12.09) | 7.87 (2.95-12.32) | 7.38 (3.27-11.92) | 0.004 |
| D-dimer, ng/mL | 340 (230-560) | 340 (230-550) | 350 (230-555) | 0.010 |
| Peak cTnI, ng/mL | 2.68 (0.79-9.10) | 2.65 (0.79-8.91) | 2.75 (0.80-9.72) | 0.003 |
| Coronary angiography findings |  |  |  |  |
| Culprit lesion, n (%) |  |  |  |  |
| LM | 6.6 (1.4) | 3.3 (1.4) | 3.2 (1.4) | 0.017 |
| LAD | 102.3 (22.0) | 51.0 (21.9) | 51.3 (22.0) |  |
| LCX | 105.1 (22.6) | 53.0 (22.7) | 52.1 (22.4) |  |
| RCA | 249.6 (53.6) | 125.0 (53.6) | 124.7 (53.6) |  |
| Bypass grafts | 2.2 (0.5) | 1.0 (0.4) | 1.2 (0.5) |  |
| Multivessel diseases, n (%) | 373.4 (80.2) | 186.3 (79.9) | 187.1 (80.5) | 0.016 |
| Pre-PCI TIMI 0 flow, n (%) | 310.2 (66.6) | 155.0 (66.4) | 155.3 (66.8) | 0.008 |
| Post-PCI TIMI 3 flow, n (%) | 451.5 (96.9) | 226.3 (97.0) | 225.2 (96.9) | 0.007 |
| D2B time ≥ 120 mins, n (%) | 253.5 (54.4) | 126.7 (54.3) | 126.9 (54.6) | 0.006 |
| CR before discharge, n (%) | 196.3 (42.1) | 98.4 (42.2) | 97.9 (42.1) | 0.002 |
| Medications at discharge |  |  |  |  |
| Aspirin, n (%) | 459.9 (98.7) | 230.3 (98.7) | 229.6 (98.8) | 0.006 |
| P2Y_12_ inhibitors, n (%) | 461.7 (99.1) | 231.2 (99.1) | 230.5 (99.1) | 0.003 |
| Statins, n (%) | 448.2 (96.2) | 224.4 (96.2) | 223.9 (96.3) | 0.006 |
| β blockers, n (%) | 390.4 (83.8) | 195.1 (83.6) | 195.3 (84.0) | 0.011 |

PSMW = propensity score matching weight, ACEI = angiotensin-converting enzyme inhibitor, ARB = angiotensin receptor blocker, SMD = standardized mean difference, PCI = percutaneous coronary intervention, CABG = coronary artery bypass grafting, STEMI = ST-segment elevation myocardial infarction, SBP = systolic blood pressure, DBP = diastolic blood pressure, EF = ejection fraction, eGFR = estimated glomerular filtration rate, LDL-C = low-density lipoprotein cholesterol, hsCRP = high-sensitivity C-reactive protein, cTnI = cardiac troponin I, LM = left main, LAD = left anterior descending artery, LCX = left circumflex, RCA = right coronary artery, TIMI flow = Thrombolysis In Myocardial Infarction grade flow, D2B time = door-to-balloon time, CR = complete revascularization.

**Supplementary table 21.** Baseline characteristics of non-hypertensive patients in PSMW dataset by ACEI/ARB medication.

| Variables | All patients (N=615.89) | No ACEI/ARB (N=309.31) | ACEI/ARB (N=306.58) | SMD |
| --- | --- | --- | --- | --- |
| Age, years | 56.3 ± 11.5 | 56.2 ± 11.3 | 56.3 ± 11.8 | 0.005 |
| Male sex, n (%) | 522.3 (84.8) | 263.4 (85.1) | 258.9 (84.5) | 0.019 |
| Hypertension, n (%) | 0 (0) | 0 (0) | 0 (0) | <0.001 |
| Diabetes, n (%) | 178.8 (29.0) | 89.7 (29.0) | 89.1 (29.1) | 0.002 |
| History of PCI or CABG, n (%) | 52.8 (8.6) | 26.2 (8.5) | 26.6 (8.7) | 0.007 |
| Peripheral artery diseases, n (%) | 12.8 (2.1) | 6.5 (2.1) | 6.3 (2.1) | 0.002 |
| STEMI, n (%) | 540.2 (87.7) | 271.7 (87.9) | 268.5 (87.6) | 0.008 |
| Hemodynamics |  |  |  |  |
| Heart rate, bpm | 75.4 ± 13.9 | 75.6 ± 13.7 | 75.2 ± 14.2 | 0.008 |
| SBP, mmHg | 118.1 ± 14.9 | 118.0 ± 15.4 | 118.1 ± 14.4 | 0.025 |
| DBP, mmHg | 71.0 ± 11.1 | 70.9 ± 10.6 | 71.0 ± 11.5 | 0.007 |
| EF, % | 57.5 ± 4.0 | 57.5 ± 4.0 | 57.5 ± 4.0 | 0.015 |
| Cardiac arrest, n (%) | 16.1 (2.6) | 8.7 (2.8) | 7.4 (2.4) | 0.010 |
| Lab tests |  |  |  |  |
| Creatinine, μmoI/L | 78.7 ± 19.6 | 78.8 ± 22.5 | 78.6 ± 16.2 | 0.008 |
| eGFR, ml/min | 88.5 (76.3-102.0) | 88.5 (77.7-102.2) | 88.5 (75.7-101.0) | 0.003 |
| LDL-C, mmol/L | 2.8 ± 0.9 | 2.8 ± 0.9 | 2.8 ± 0.9 | 0.001 |
| hsCRP, mg/L | 5.11 (2.21-11.33) | 5.41 (2.07-11.39) | 5.04 (2.31-11.26) | 0.012 |
| D-dimer, ng/mL | 290 (210-420) | 290 (210-420) | 280 (210-420) | 0.006 |
| Peak cTnI, ng/mL | 2.45 (0.66-7.78) | 2.34 (0.67-8.23) | 2.55 (0.59-7.58) | 0.004 |
| Coronary angiography findings |  |  |  |  |
| Culprit lesion, n (%) |  |  |  |  |
| LM | 7.2 (1.2) | 3.4 (1.1) | 3.8 (1.2) | 0.015 |
| LAD | 174.3 (28.3) | 87.9 (28.4) | 86.4 (28.2) |  |
| LCX | 113.6 (18.5) | 56.5 (18.3) | 57.1 (18.6) |  |
| RCA | 320.7 (52.1) | 161.5 (52.2) | 159.2 (51.9) |  |
| Bypass grafts | 0 (0) | 0 (0) | 0 (0) |  |
| Multivessel diseases, n (%) | 440.1 (71.5) | 220.7 (71.4) | 219.4 (71.6) | 0.004 |
| Pre-PCI TIMI 0 flow, n (%) | 401.8 (65.2) | 202.0 (65.3) | 199.9 (65.2) | 0.002 |
| Post-PCI TIMI 3 flow, n (%) | 602.2 (97.8) | 302.0 (97.6) | 300.2 (97.9) | 0.018 |
| D2B time ≥ 120 mins, n (%) | 330.4 (53.6) | 165.0 (53.3) | 165.4 (54.0) | 0.013 |
| CR before discharge, n (%) | 283.8 (46.1) | 142.8 (46.2) | 141.1 (46.0) | 0.003 |
| Medications at discharge |  |  |  |  |
| Aspirin, n (%) | 613.6 (99.6) | 308.0 (99.6) | 305.6 (99.7) | 0.016 |
| P2Y_12_ inhibitors, n (%) | 611.1 (99.2) | 306.5 (99.1) | 304.6 (99.3) | 0.029 |
| Statins, n (%) | 574.7 (93.3) | 288.6 (93.3) | 286.1 (93.3) | 0.001 |
| β blockers, n (%) | 497.6 (80.8) | 249.4 (80.6) | 248.2 (81.0) | 0.008 |

PSMW = propensity score matching weight, ACEI = angiotensin-converting enzyme inhibitor, ARB = angiotensin receptor blocker, SMD = standardized mean difference, PCI = percutaneous coronary intervention, CABG = coronary artery bypass grafting, STEMI = ST-segment elevation myocardial infarction, SBP = systolic blood pressure, DBP = diastolic blood pressure, EF = ejection fraction, eGFR, estimated glomerular filtration rate, LDL-C = low-density lipoprotein cholesterol, hsCRP = high-sensitivity C-reactive protein, cTnI = cardiac troponin I, LM = left main, LAD = left anterior descending artery, LCX = left circumflex, RCA = right coronary artery, TIMI flow = Thrombolysis In Myocardial Infarction grade flow, D2B time = door-to-balloon time, CR = complete revascularization.

**Supplementary table 22.** Baseline characteristics of anterior infarction patients in PSMW dataset by ACEI/ARB medication.

| Variables | All patients (N=293.89) | No ACEI/ARB (N=147.03) | ACEI/ARB (N=146.87) | SMD |
| --- | --- | --- | --- | --- |
| Age, years | 56.7 ± 11.6 | 57.7 ± 11.1 | 56.8 ± 12.0 | 0.006 |
| Male sex, n (%) | 232.0 (78.9) | 117.0 (79.6) | 115.0 (78.3) | 0.033 |
| Hypertension, n (%) | 109.5 (37.3) | 54.3 (36.9) | 55.2 (37.6) | 0.014 |
| Diabetes, n (%) | 98.3 (33.4) | 49.0 (33.3) | 49.3 (33.6) | 0.006 |
| History of PCI or CABG, n (%) | 30.2 (10.3) | 15.9 (10.8) | 14.4 (9.8) | 0.033 |
| Peripheral artery diseases, n (%) | 15.5 (5.3) | 7.7 (5.2) | 7.9 (5.3) | 0.006 |
| STEMI, n (%) | 237.0 (80.6) | 118.0 (80.2) | 119.0 (81.0) | 0.021 |
| Hemodynamics |  |  |  |  |
| Heart rate, bpm | 74.4 ± 12.0 | 74.4 ± 11.5 | 74.4 ± 12.5 | 0.002 |
| SBP, mmHg | 122.2 ± 15.6 | 122.0 ± 16.2 | 122.5 ± 15.0 | 0.033 |
| DBP, mmHg | 74.9 ± 11.7 | 74.6 ± 12.3 | 75.2 ± 11.2 | 0.051 |
| EF, % | 56.6 ± 4.5 | 56.7 ± 4.5 | 56.6 ± 4.5 | 0.018 |
| Cardiac arrest, n (%) | 7.9 (2.7) | 4.0 (2.7) | 3.9 (2.7) | 0.001 |
| Lab tests |  |  |  |  |
| Creatinine, μmoI/L | 78.2 ± 21.5 | 78.3 ± 24.2 | 78.1 ± 18.4 | 0.005 |
| eGFR, ml/min | 87.9 (75.7-102.5) | 86.9 (76.3-102.6) | 88.8 (75.3-101.1) | 0.073 |
| LDL-C, mmol/L | 2.6 ± 0.8 | 2.6 ± 0.8 | 2.6 ± 0.8 | 0.015 |
| hsCRP, mg/L | 5.15 (2.17-11.33) | 4.95 (2.05-11.75) | 5.51 (2.28-11.20) | 0.011 |
| D-dimer, ng/mL | 270 (200-400) | 270 (200-420) | 270 (210-400) | 0.003 |
| Peak cTnI, ng/mL | 2.45 (0.64-7.43) | 2.45 (0.64-7.43) | 2.52 (0.66-7.64) | 0.023 |
| Coronary angiography findings |  |  |  |  |
| Culprit lesion, n (%) |  |  |  |  |
| LM | 18.7 (6.4) | 9.6 (6.6) | 9.1 (6.2) | 0.015 |
| LAD | 275.2 (93.6) | 137.4 (93.5) | 137.8 (93.8) |  |
| LCX | 0 (0) | 0 (0) | 0 (0) |  |
| RCA | 0 (0) | 0 (0) | 0 (0) |  |
| Bypass grafts | 0 (0) | 0 (0) | 0 (0) |  |
| Multivessel diseases, n (%) | 174.2 (59.3) | 87.8 (59.7) | 86.4 (58.8) | 0.018 |
| Pre-PCI TIMI 0 flow, n (%) | 158.7 (54.0) | 80.3 (54.6) | 78.4 (53.4) | 0.025 |
| Post-PCI TIMI 3 flow, n (%) | 284.5 (96.8) | 142.1 (96.6) | 142.4 (97.0) | 0.019 |
| D2B time ≥ 120 mins, n (%) | 178.1 (60.6) | 89.2 (60.6) | 89.0 (60.6) | 0.001 |
| CR before discharge, n (%) | 168.0 (57.1) | 83.4 (56.8) | 84.5 (57.5) | 0.016 |
| Medications at discharge |  |  |  |  |
| Aspirin, n (%) | 291.6 (99.2) | 146.0 (99.3) | 145.6 (99.1) | 0.024 |
| P2Y_12_ inhibitors, n (%) | 291.8 (99.3) | 145.9 (99.3) | 145.9 (99.3) | 0.007 |
| Statins, n (%) | 278.2 (94.7) | 138.7 (94.4) | 139.5 (95.0) | 0.027 |
| β blockers, n (%) | 266.7 (90.8) | 133.4 (90.8) | 133.3 (90.7) | 0.001 |

PSMW = propensity score matching weight, ACEI = angiotensin-converting enzyme inhibitor, ARB = angiotensin receptor blocker, SMD = standardized mean difference, PCI = percutaneous coronary intervention, CABG = coronary artery bypass grafting, STEMI = ST-segment elevation myocardial infarction, SBP = systolic blood pressure, DBP = diastolic blood pressure, EF = ejection fraction, eGFR, estimated glomerular filtration rate, LDL-C = low-density lipoprotein cholesterol, hsCRP = high-sensitivity C-reactive protein, cTnI = cardiac troponin I, LM = left main, LAD = left anterior descending artery, LCX = left circumflex, RCA = right coronary artery, TIMI flow = Thrombolysis In Myocardial Infarction grade flow, D2B time = door-to-balloon time, CR = complete revascularization.

**Supplementary table 23.** Baseline characteristics of non-anterior infarction patients in PSMW dataset by ACEI/ARB medication.

| Variables | All patients (N=808.07) | No ACEI/ARB (N=406.21) | ACEI/ARB (N=401.86) | SMD |
| --- | --- | --- | --- | --- |
| Age, years | 58.6 ± 11.5 | 58.6 ± 11.5 | 58.6 ± 11.5 | 0.002 |
| Male sex, n (%) | 649.3 (80.4) | 327.0 (80.5) | 322.3 (80.2) | 0.008 |
| Hypertension, n (%) | 359.2 (44.5) | 180.5 (44.4) | 178.7 (44.5) | <0.001 |
| Diabetes, n (%) | 245.6 (30.4) | 122.8 (30.2) | 122.8 (30.6) | 0.007 |
| History of PCI or CABG, n (%) | 91.0 (11.3) | 45.4 (11.2) | 45.6 (11.3) | 0.005 |
| Peripheral artery diseases, n (%) | 24.6 (3.0) | 11.8 (2.9) | 12.8 (3.2) | 0.017 |
| STEMI, n (%) | 706.9 (87.5) | 354.3 (87.2) | 352.7 (87.8) | 0.017 |
| Hemodynamics |  |  |  |  |
| Heart rate, bpm | 76.1 ± 14.8 | 76.2 ± 15.0 | 76.0 ± 14.6 | 0.010 |
| SBP, mmHg | 119.2 ± 16.3 | 119.1 ± 16.7 | 119.2 ± 16.0 | 0.003 |
| DBP, mmHg | 70.9 ± 11.3 | 70.9 ± 10.9 | 71.0 ± 11.8 | 0.006 |
| EF, % | 57.8 ± 3.8 | 57.8 ± 3.9 | 57.8 ± 3.8 | 0.005 |
| Cardiac arrest, n (%) | 25.7 (3.2) | 12.9 (3.2) | 12.8 (3.2) | 0.001 |
| Lab tests |  |  |  |  |
| Creatinine, μmoI/L | 78.2 ± 21.5 | 80.8 ± 23.4 | 80.6 ± 20.0 | 0.011 |
| eGFR, ml/min | 85.6 (72.0-99.0) | 85.7 (72.2-99.8) | 85.5 (71.3-98.2) | 0.008 |
| LDL-C, mmol/L | 2.7 ± 0.9 | 2.7 ± 0.9 | 2.7 ± 0.9 | 0.009 |
| hsCRP, mg/L | 6.50 (2.78-11.75) | 6.68 (2.69-11.72) | 6.20 (2.97-11.81) | 0.011 |
| D-dimer, ng/mL | 320 (220-500) | 320 (220-490) | 329 (220-520) | 0.010 |
| Peak cTnI, ng/mL | 2.59 (0.75-8.40) | 2.40 (0.75-8.40) | 2.65 (0.74-8.34) | <0.001 |
| Coronary angiography findings |  |  |  |  |
| Culprit lesion, n (%) |  |  |  |  |
| LM | 0 (0) | 0 (0) | 0 (0) | 0.020 |
| LAD | 0 (0) | 0 (0) | 0 (0) |  |
| LCX | 220.6 (27.3) | 110.1 (27.1) | 110.6 (27.5) |  |
| RCA | 585.1 (72.4) | 295.1 (72.7) | 289.9 (72.1) |  |
| Bypass grafts | 2.4 (0.3) | 1.0 (0.2) | 1.4 (0.3) |  |
| Multivessel diseases, n (%) | 656.2 (81.2) | 327.9 (80.7) | 328.3 (81.7) | 0.025 |
| Pre-PCI TIMI 0 flow, n (%) | 567.5 (70.2) | 283.2 (69.7) | 284.3 (70.8) | 0.023 |
| Post-PCI TIMI 3 flow, n (%) | 789.0 (97.6) | 397.0 (97.7) | 392.0 (97.6) | 0.013 |
| D2B time ≥ 120 mins, n (%) | 423.3 (52.4) | 212.4 (52.3) | 210.9 (52.5) | 0.004 |
| CR before discharge, n (%) | 318.3 (39.4) | 160.6 (39.5) | 157.7 (39.2) | 0.006 |
| Medications at discharge |  |  |  |  |
| Aspirin, n (%) | 802.6 (99.3) | 403.2 (99.3) | 399.4 (99.4) | 0.017 |
| P2Y_12_ inhibitors, n (%) | 801.1 (99.1) | 402.3 (99.0) | 398.9 (99.3) | 0.025 |
| Statins, n (%) | 762.1 (94.3) | 383.1 (94.3) | 379.0 (94.3) | 0.001 |
| β blockers, n (%) | 626.9 (77.6) | 313.7 (77.2) | 313.2 (77.9) | 0.017 |

PSMW = propensity score matching weight, ACEI = angiotensin-converting enzyme inhibitor, ARB = angiotensin receptor blocker, SMD = standardized mean difference, PCI = percutaneous coronary intervention, CABG = coronary artery bypass grafting, STEMI = ST-segment elevation myocardial infarction, SBP = systolic blood pressure, DBP = diastolic blood pressure, EF = ejection fraction, eGFR, estimated glomerular filtration rate, LDL-C = low-density lipoprotein cholesterol, hsCRP = high-sensitivity C-reactive protein, cTnI = cardiac troponin I, LM = left main, LAD = left anterior descending artery, LCX = left circumflex, RCA = right coronary artery, TIMI flow = Thrombolysis In Myocardial Infarction grade flow, D2B time = door-to-balloon time, CR = complete revascularization.

**Supplementary table 24.** Baseline characteristics of patients in the PSMW dataset stratified by the medication of ACEI.

| Variables | All patients (N=1101.33) | No ACEI  (N=552.97) | ACEI  (N=548.35) | SMD |
| --- | --- | --- | --- | --- |
| Age, years | 58.1 ± 11.5 | 58.1 ± 11.4 | 58.1 ± 11.6 | 0.004 |
| Male sex, n (%) | 879.9 (79.9) | 443.5 (80.2) | 436.5 (79.6) | 0.015 |
| Hypertension, n (%) | 468.2 (42.5) | 233.8 (42.3) | 234.4 (42.8) | 0.010 |
| Diabetes, n (%) | 342.5 (31.1) | 170.9 (30.9) | 171.6 (31.3) | 0.008 |
| History of PCI or CABG, n (%) | 120.7 (11.0) | 60.4 (10.9) | 60.2 (11.0) | 0.002 |
| Peripheral artery diseases, n (%) | 41.8 (3.8) | 20.1 (3.6) | 21.7 (4.0) | 0.017 |
| STEMI, n (%) | 943.6 (85.7) | 472.5 (85.4) | 471.1 (85.9) | 0.013 |
| Hemodynamics |  |  |  |  |
| Heart rate, bpm | 75.6 ± 14.0 | 75.7 ± 14.1 | 75.5 ± 13.9 | 0.013 |
| SBP, mmHg | 119.9 ± 16.0 | 119.8 ± 16.6 | 120.0 ± 15.4 | 0.011 |
| DBP, mmHg | 71.9 ± 11.5 | 71.8 ± 11.4 | 72.0 ± 11.6 | 0.013 |
| EF, % | 57.5 ± 4.1 | 57.5 ± 4.1 | 57.5 ± 4.0 | 0.007 |
| Cardiac arrest, n (%) | 32.7 (3.0) | 16.5 (3.0) | 16.1 (2.9) | 0.003 |
| Lab tests |  |  |  |  |
| Creatinine, μmoI/L | 79.9 ± 21.4 | 79.9 ± 23.5 | 79.8 ± 19.1 | 0.009 |
| eGFR, ml/min | 86.2 (73.3-100.0) | 86.4 (74.0-100.5) | 86.1 (72.3-99.1) | 0.024 |
| LDL-C, mmol/L | 2.7 ± 0.9 | 2.7 ± 0.9 | 2.7 ± 0.9 | 0.001 |
| hsCRP, mg/L | 5.98 (2.52-11.67) | 6.24 (2.40-11.71) | 5.89 (2.63-11.61) | 0.006 |
| D-dimer, ng/mL | 310 (220-470) | 310 (220-470) | 310 (220-474) | 0.012 |
| Peak cTnI, ng/mL | 2.50 (0.72-7.92) | 2.40 (0.69-8.23) | 2.55 (0.73-7.78) | 0.006 |
| Coronary angiography findings |  |  |  |  |
| Culprit lesion, n (%) |  |  |  |  |
| LM | 18.0 (1.6) | 9.2 (1.7) | 8.9 (1.6) | 0.017 |
| LAD | 278.2 (25.3) | 139.7 (25.3) | 138.5 (25.3) |  |
| LCX | 220.3 (20.0) | 110.0 (19.9) | 110.3 (20.1) |  |
| RCA | 582.4 (52.9) | 293.1 (53.0) | 289.3 (52.8) |  |
| Bypass grafts | 2.4 (0.2) | 1.0 (0.2) | 1.4 (0.3) |  |
| Multivessel diseases, n (%) | 829.5 (75.3) | 415.1 (75.1) | 414.5 (75.6) | 0.012 |
| Pre-PCI TIMI 0 flow, n (%) | 724.3 (65.8) | 362.5 (65.5) | 361.8 (66.0) | 0.009 |
| Post-PCI TIMI 3 flow, n (%) | 1073.2 (97.4) | 538.9 (97.5) | 534.3 (97.4) | 0.002 |
| D2B time ≥ 120 mins, n (%) | 600.0 (54.5) | 301.0 (54.4) | 298.9 (54.5) | 0.001 |
| CR before discharge, n (%) | 485.8 (44.1) | 243.6 (44.1) | 242.2 (44.2) | 0.002 |
| Medications at discharge |  |  |  |  |
| Aspirin, n (%) | 1093.9 (99.3) | 549.1 (99.3) | 544.9 (99.4) | 0.008 |
| P2Y_12_ inhibitors, n (%) | 1094.7 (99.4) | 549.4 (99.4) | 545.4 (99.5) | 0.013 |
| Statins, n (%) | 1039.8 (94.4) | 521.6 (94.3) | 518.2 (94.5) | 0.008 |
| β blockers, n (%) | 893.0 (81.1) | 446.5 (80.7) | 446.5 (81.4) | 0.017 |

PSMW = propensity score matching weight, ACEI = angiotensin-converting enzyme inhibitor, SMD = standardized mean difference, PCI = percutaneous coronary intervention, CABG = coronary artery bypass grafting, STEMI = ST-segment elevation myocardial infarction, SBP = systolic blood pressure, DBP = diastolic blood pressure, EF = ejection fraction, eGFR, estimated glomerular filtration rate, LDL-C = low-density lipoprotein cholesterol, hsCRP = high-sensitivity C-reactive protein, cTnI = cardiac troponin I, LM = left main, LAD = left anterior descending artery, LCX = left circumflex, RCA = right coronary artery, TIMI flow = Thrombolysis In Myocardial Infarction grade flow, D2B time = door-to-balloon time, CR = complete revascularization.

**Supplementary table 25.** Baseline characteristics of patients in the PSMW dataset stratified by the medication of ARB.

| Variables | All patients (N=319.74) | No ARB  (N=159.76) | ARB  (N=159.99) | SMD |
| --- | --- | --- | --- | --- |
| Age, years | 58.6 ± 12.0 | 58.6 ± 11.5 | 58.6 ± 12.4 | 0.007 |
| Male sex, n (%) | 243.8 (76.3) | 120.9 (75.7) | 122.9 (76.8) | 0.028 |
| Hypertension, n (%) | 249.6 (78.1) | 124.6 (78.0) | 125.0 (78.1) | 0.003 |
| Diabetes, n (%) | 112.6 (35.2) | 57.1 (35.8) | 55.4 (34.6) | 0.023 |
| History of PCI or CABG, n (%) | 52.0 (16.3) | 27.0 (16.9) | 25.0 (15.6) | 0.034 |
| Peripheral artery diseases, n (%) | 9.0 (2.8) | 5.0 (3.1) | 4.0 (2.5) | 0.038 |
| STEMI, n (%) | 266.3 (83.3) | 133.3 (83.4) | 133.0 (83.1) | 0.008 |
| Hemodynamics |  |  |  |  |
| Heart rate, bpm | 74.7 ± 13.4 | 74.7 ± 13.4 | 74.6 ± 13.4 | 0.012 |
| SBP, mmHg | 128.5 ± 18.3 | 128.5 ± 18.5 | 128.6 ± 18.1 | 0.004 |
| DBP, mmHg | 75.1 ± 12.7 | 75.0 ± 12.6 | 75.2 ± 12.7 | 0.018 |
| EF, % | 58.2 ± 4.1 | 58.1 ± 4.2 | 58.2 ± 4.0 | 0.011 |
| Cardiac arrest, n (%) | 6.5 (2.0) | 3.5 (2.2) | 3.0 (1.9) | 0.022 |
| Lab tests |  |  |  |  |
| Creatinine, μmoI/L | 83.6 ± 25.5 | 83.4 ± 28.4 | 83.7 ± 22.3 | 0.012 |
| eGFR, ml/min | 82.7 (68.0-95.7) | 82.6 (70.5-98.0) | 82.6 (65.3-94.3) | 0.093 |
| LDL-C, mmol/L | 2.7 ± 0.9 | 2.7 ± 1.0 | 2.7 ± 0.9 | 0.014 |
| hsCRP, mg/L | 5.88 (2.51-11.44) | 5.64 (2.34-11.39) | 5.90 (2.58-11.38) | 0.006 |
| D-dimer, ng/mL | 300 (210-510) | 300 (215-490) | 300 (200-510) | 0.028 |
| Peak cTnI, ng/mL | 2.06 (0.50-6.71) | 2.06 (0.61-6.73) | 2.04 (0.45-6.22) | 0.011 |
| Coronary angiography findings |  |  |  |  |
| Culprit lesion, n (%) |  |  |  |  |
| LM | 0 (0) | 0 (0) | 0 (0) | 0.029 |
| LAD | 91.4 (28.6) | 46.4 (29.1) | 45.0 (28.1) |  |
| LCX | 76.4 (23.9) | 37.3 (23.3) | 39.1 (24.5) |  |
| RCA | 151.9 (47.5) | 76.1 (47.6) | 75.8 (47.4) |  |
| Bypass grafts | 0 (0) | 0 (0) | 0 (0) |  |
| Multivessel diseases, n (%) | 239.3 (74.8) | 119.0 (74.5) | 120.3 (75.2) | 0.017 |
| Pre-PCI TIMI 0 flow, n (%) | 203.3 (63.6) | 103.2 (64.6) | 100.2 (62.6) | 0.041 |
| Post-PCI TIMI 3 flow, n (%) | 313.0 (97.9) | 156.1 (97.7) | 156.9 (98.1) | 0.025 |
| D2B time ≥ 120 mins, n (%) | 195.6 (61.2) | 96.8 (60.6) | 98.8 (61.8) | 0.024 |
| CR before discharge, n (%) | 150.8 (47.2) | 76.0 (47.6) | 74.8 (46.7) | 0.017 |
| Medications at discharge |  |  |  |  |
| Aspirin, n (%) | 317.8 (99.4) | 158.8 (99.4) | 159.0 (99.4) | 0.003 |
| P2Y_12_ inhibitors, n (%) | 317.5 (99.3) | 158.5 (99.2) | 159.0 (99.4) | 0.020 |
| Statins, n (%) | 306.2 (95.8) | 153.2 (95.9) | 153.0 (95.6) | 0.015 |
| β blockers, n (%) | 285.0 (89.1) | 142.7 (89.3) | 142.3 (88.9) | 0.013 |

PSMW = propensity score matching weight, ARB = angiotensin receptor blocker, SMD = standardized mean difference, PCI = percutaneous coronary intervention, CABG = coronary artery bypass grafting, STEMI = ST-segment elevation myocardial infarction, SBP = systolic blood pressure, DBP = diastolic blood pressure, EF = ejection fraction, eGFR, estimated glomerular filtration rate, LDL-C = low-density lipoprotein cholesterol, hsCRP = high-sensitivity C-reactive protein, cTnI = cardiac troponin I, LM = left main, LAD = left anterior descending artery, LCX = left circumflex, RCA = right coronary artery, TIMI flow = Thrombolysis In Myocardial Infarction grade flow, D2B time = door-to-balloon time, CR = complete revascularization.

**Supplementary figure 1.** Receiver operating curve (ROC) for the logistic regression model of propensity score matching for the whole cohort.

**
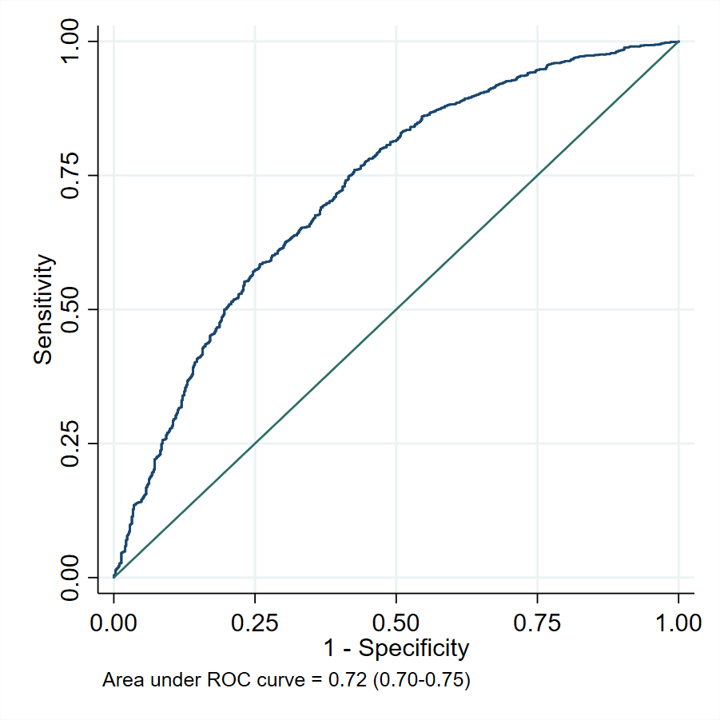
**

**Supplementary figure 2.** Receiver operating curve (ROC) for the logistic regression model of propensity score matching for various subgroups. ACEI = angiotensin-converting enzyme inhibitor, ARB = angiotensin receptor blocker.


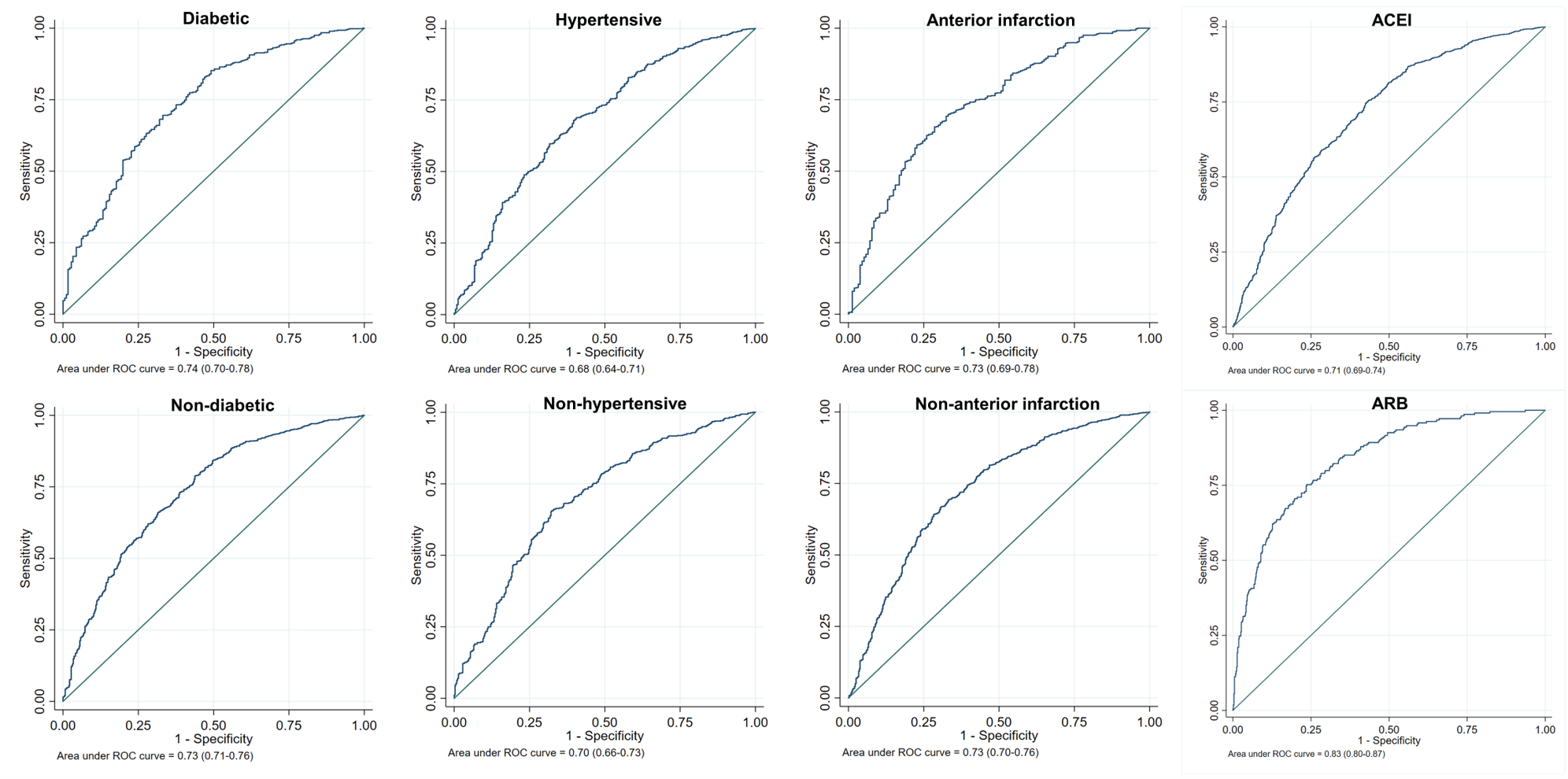


**Supplementary figure 3.** Density plots for distribution of propensity score of diabetic patients stratified by usage of ACEI/ARB in the original dataset (A), the PSM dataset (B) and PSMW dataset (C). Red line = ACEI/ARB users, blue line = ACEI/ARB non-users. ACEI = angiotensin converting enzyme inhibitors, ARB = angiotensin receptor blockers, PSM = propensity score matching, PSMW = propensity score matching weight.


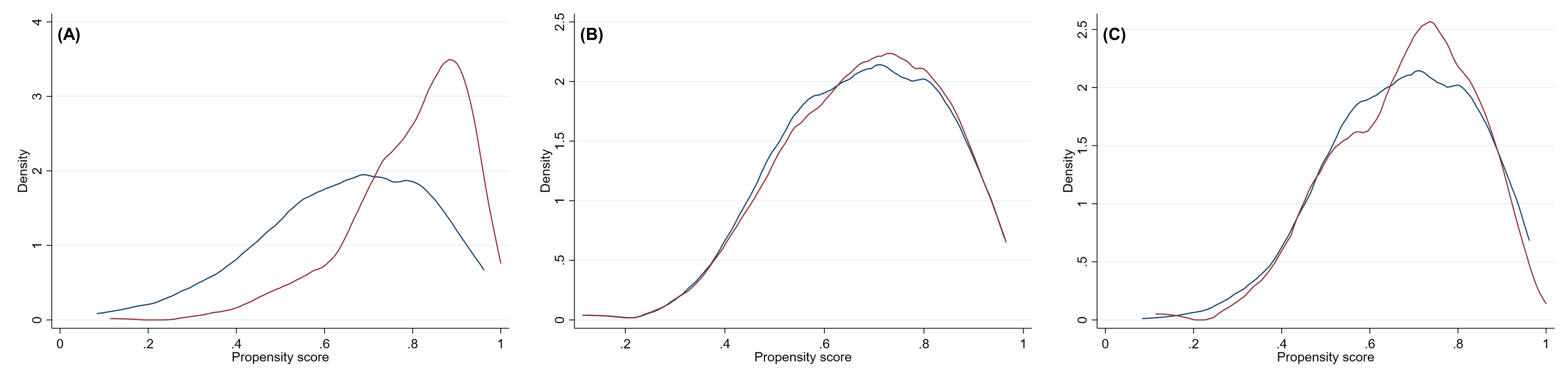


**Supplementary figure 4.** Density plots for distribution of propensity score of non-diabetic patients stratified by usage of ACEI/ARB in the original dataset (A), the PSM dataset (B) and PSMW dataset (C). Red line = ACEI/ARB users, blue line = ACEI/ARB non-users. ACEI = angiotensin converting enzyme inhibitors, ARB = angiotensin receptor blockers, PSM = propensity score matching, PSMW = propensity score matching weight.


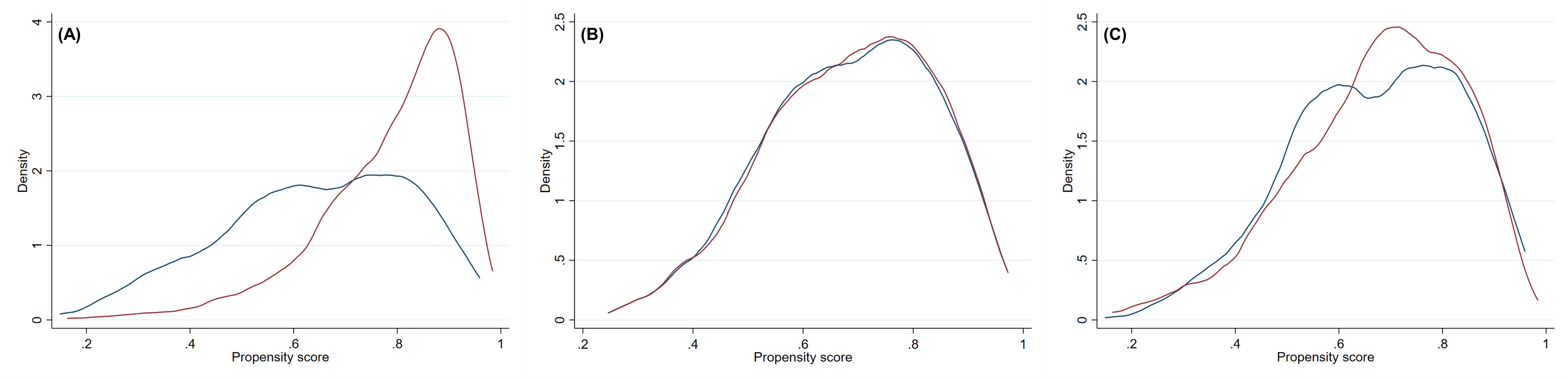


**Supplementary figure 5.** Density plots for distribution of propensity score of hypertensive patients stratified by usage of ACEI/ARB in the original dataset (A), the PSM dataset (B) and PSMW dataset (C). Red line = ACEI/ARB users, blue line = ACEI/ARB non-users. ACEI = angiotensin converting enzyme inhibitors, ARB = angiotensin receptor blockers, PSM = propensity score matching, PSMW = propensity score matching weight.


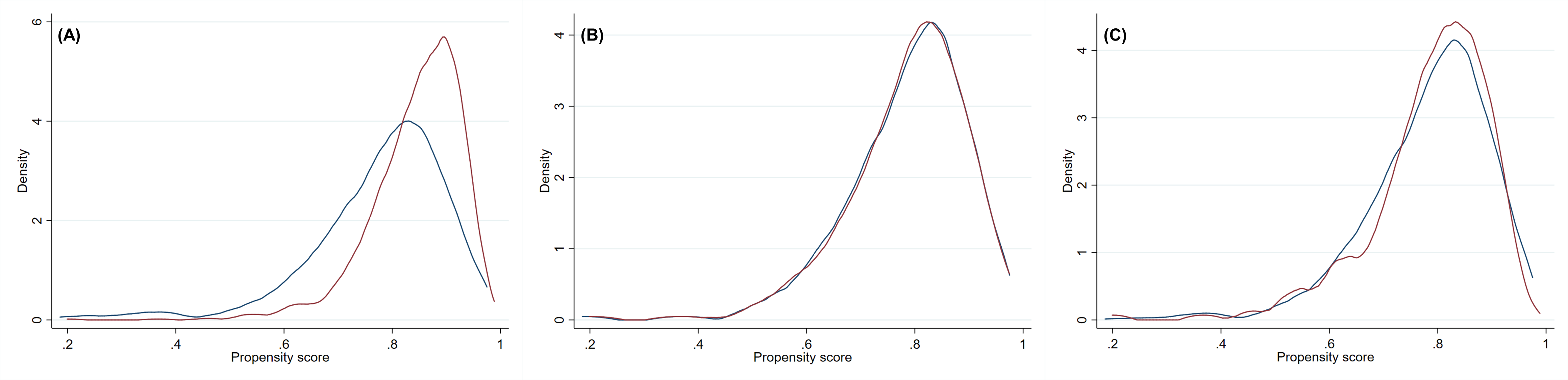


**Supplementary figure 6.** Density plots for distribution of propensity score of non-hypertensive patients stratified by usage of ACEI/ARB in the original dataset (A), the PSM dataset (B) and PSMW dataset (C). Red line = ACEI/ARB users, blue line = ACEI/ARB non-users. ACEI = angiotensin converting enzyme inhibitors, ARB = angiotensin receptor blockers, PSM = propensity score matching, PSMW = propensity score matching weight.


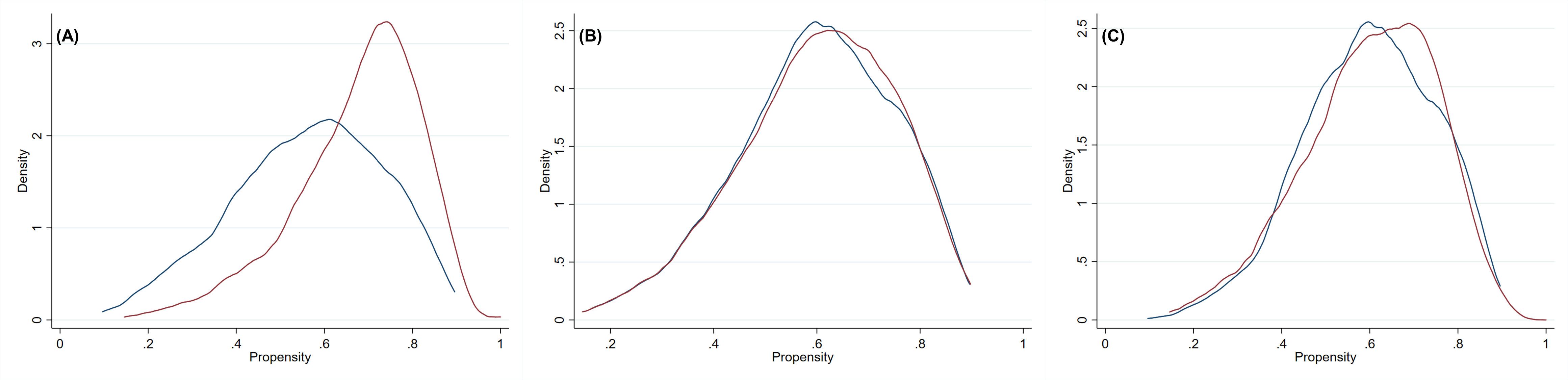


**Supplementary figure 7.** Density plots for distribution of propensity score of anterior infarction patients stratified by usage of ACEI/ARB in the original dataset (A), the PSM dataset (B) and PSMW dataset (C). Red line = ACEI/ARB users, blue line = ACEI/ARB non-users. ACEI = angiotensin converting enzyme inhibitors, ARB = angiotensin receptor blockers, PSM = propensity score matching, PSMW = propensity score matching weight.


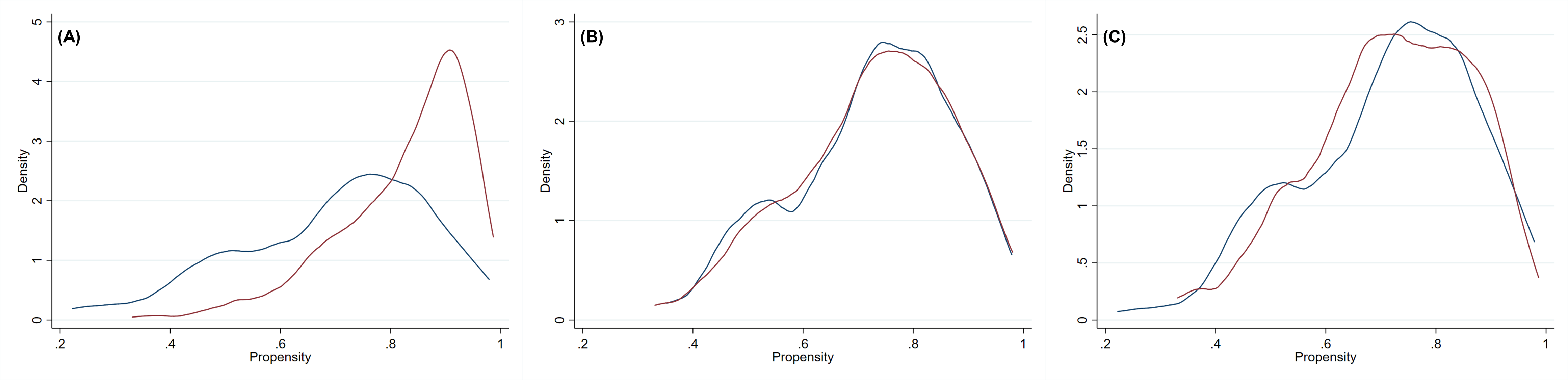


**Supplementary figure 8.** Density plots for distribution of propensity score of non-anterior infarction patients stratified by usage of ACEI/ARB in the original dataset (A), the PSM dataset (B) and PSMW dataset (C). Red line = ACEI/ARB users, blue line = ACEI/ARB non-users. ACEI = angiotensin converting enzyme inhibitors, ARB = angiotensin receptor blockers, PSM = propensity score matching, PSMW = propensity score matching weight.


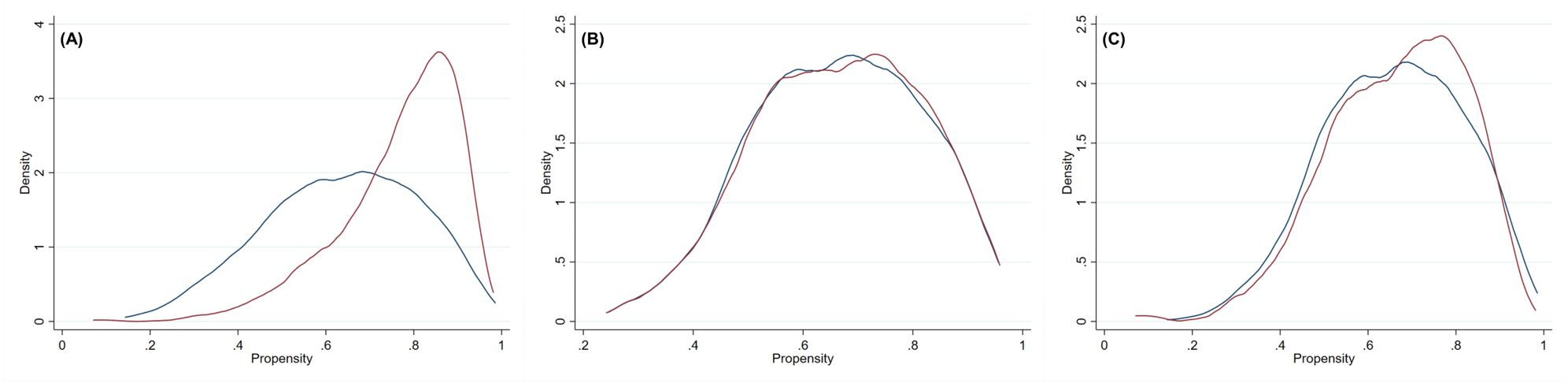


**Supplementary figure 9.** Density plots for distribution of propensity score of patients stratified by usage of ACEI in the original dataset (A), the PSM dataset (B) and PSMW dataset (C). Red line = ACEI users, blue line = ACEI/ARB non-users. ACEI = angiotensin converting enzyme inhibitors, ARB = angiotensin receptor blockers, PSM = propensity score matching, PSMW = propensity score matching weight.


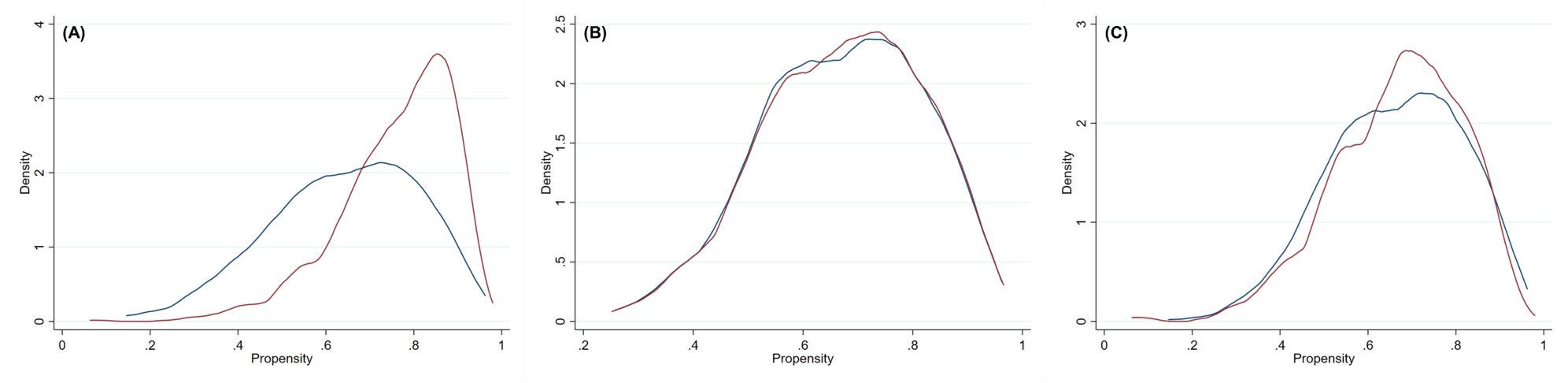


**Supplementary figure 10.** Density plots for distribution of propensity score of patients stratified by usage of ARB in the original dataset (A), the PSM dataset (B) and PSMW dataset (C). Red line = ARB users, blue line = ACEI/ARB non-users. ACEI = angiotensin converting enzyme inhibitors, ARB = angiotensin receptor blockers, PSM = propensity score matching, PSMW = propensity score matching weight.


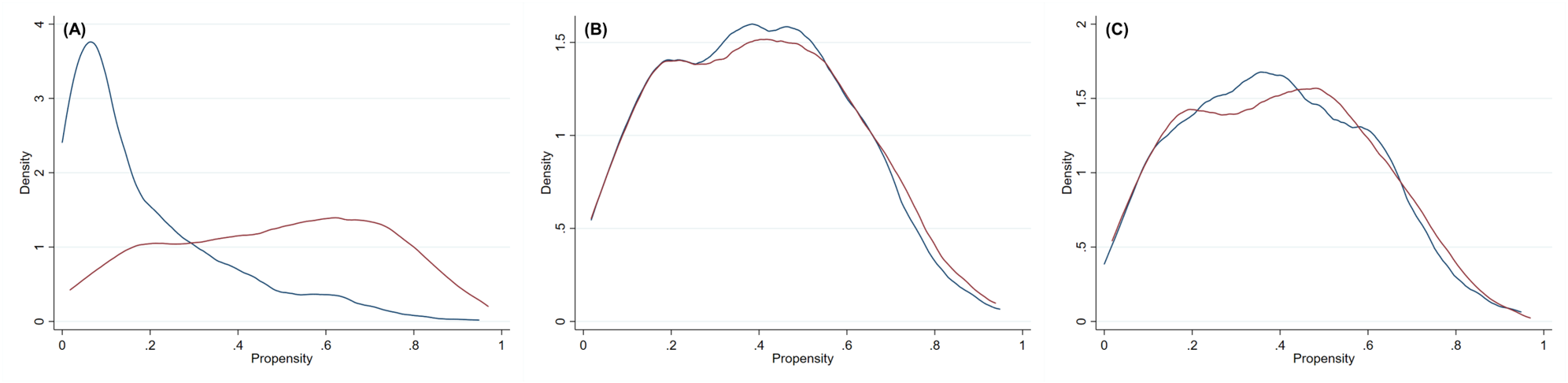

Supplement: Supplementary file 1 [file DataSheet1.docx]
